# Vaccine efficacy against SARS-CoV-2 for Pfizer BioNTech, Moderna, and AstraZeneca vaccines: a systematic review

**DOI:** 10.3389/fpubh.2023.1229716

**Published:** 2023-10-24

**Authors:** Lia Reynolds, Cate Dewey, Ghaid Asfour, Matthew Little

**Affiliations:** ^1^Department of Population Medicine, Ontario Veterinary College, University of Guelph, Guelph, ON, Canada; ^2^School of Public Health and Social Policy, Faculty of Human and Social Development, University of Victoria, Victoria, BC, Canada

**Keywords:** SARS-CoV-2, COVID-19, vaccine efficacy, systematic review, mRNA vaccines, AstraZeneca vaccine

## Abstract

The purpose of this systematic review was to report on the vaccine efficacy (VE) of three SARS-CoV-2 vaccines approved by Health Canada: Pfizer BioNTech, Moderna, and AstraZeneca. Four databases were searched for primary publications on population-level VE. Ninety-two publications matched the inclusion criteria, and the extracted data were separated by vaccine type: mRNA vaccines (Pfizer and Moderna) and the AstraZeneca vaccine. The median VE for PCR-positive patients and various levels of clinical disease was determined for the first and second doses of both vaccine types against multiple SARS-CoV-2 variants. The median VE for PCR-positive infections against unidentified variants from an mRNA vaccine was 64.5 and 89%, respectively, after one or two doses. The median VE for PCR-positive infections against unidentified variants from the AstraZeneca vaccine was 53.4 and 69.6%, respectively, after one or two doses. The median VE for two doses of mRNA for asymptomatic, symptomatic, and severe infection against unidentified variants was 85.5, 93.2, and 92.2%, respectively. The median VE for two doses of AstraZeneca for asymptomatic, symptomatic, and severe infection against unidentified variants was 69.7, 71, and 90.2%, respectively. Vaccine efficacy numerically increased from the first to the second dose, increased from the first 2 weeks to the second 2 weeks post-vaccination for both doses, but decreased after 4 months from the second dose. Vaccine efficacy did not differ by person's age.

## Introduction

As of 14 April 2023, SARS-CoV-2 has infected over 762 million people worldwide, causing approximately 6.8 million deaths (1). Several vaccines have been developed to protect people from illness due to this virus. In December 2020, the first SARS-CoV-2 vaccine, Pfizer BioNTech, was approved by the FDA for emergency use (2). Health Canada has approved the use of three SARS-CoV-2 vaccines: Pfizer, Moderna, and AstraZeneca on 9 December 2020, 23 December 2020, and 26 February 2021, respectively (3) (Table 1). Each company conducted a series of phase 3 randomized controlled trials to determine the VE. In November 2020, Pfizer determined that the VE against infection seven or more days after the second dose was 95% (4) In December 2020, Moderna determined its VE was 94.1% against SARS-CoV-2 infection 14 or more days after the second dose (5). In March 2021, AstraZeneca determined its VE was 79% against symptomatic infection 15 or more days after the second dose (6). All three vaccines required two doses to maximize efficacy. Nevertheless, these efficacy figures may not reflect the efficacy in real-world settings due to factors such as age distributions and other demographics, as well as the increased prevalence of different SARS-CoV-2 variants.

**Table 1:** Names of companies, research names, and vaccine types for vaccines included in this systematic review.

**Company**	**Research name**	**Vaccine type**
Pfizer BioNTech	BNT162b2	mRNA
Moderna	mRNA-1273	mRNA
AstraZeneca	AZD1222 (ChAdOx1)	Non-replicating viral vector

After the completion of initial publications that determined the safety and efficacy of randomized controlled trials on smaller samples of individuals, further research was completed on larger, population-level settings. The purpose of this systematic review was to determine the efficacy of the first and second doses of the mRNA and AstraZeneca vaccines against SARS-CoV-2 infections in real-world, population-level settings, between December 2020 and May 2022. These peer-reviewed studies determined the efficacy of SARS-CoV-2 vaccines based on a positive PCR test and against a range of clinical presentations, including asymptomatic, symptomatic, severe/critical infection, hospitalization, or death. Key variants of concern in this review were the original/China variant, B.1.1.7/Alpha/UK variant, B.1.351/Beta/South African variant, and B.1.617.2/Delta/Indian variant (Table 2) (7).

**Table 2:** Names of the variants of the SARS-CoV-2 virus included in selected articles.

**Country (Area) of Origin**	**WHO label (Variant Name)**	**Pango Lineage**
China	(Original variant)	
United Kingdom	Alpha (UK variant)	B.1.1.7
South Africa	Beta (South African variant)	B.1.351
India	Delta (India variant)	B.1.617.2
Brazil	Gamma (Brazil variant)	P.1, P.1.1, P.1.2, B.1.1.28
South Africa	Omicron	B.1.1.529
United States of America	Epsilon	B.1.427, B.1.429
United States of America	Iota	B.1.526, B.1.526.1, B.1.526.2
Colombia	Mu	B.1.621, B.1.621.1
Brazil	Zeta	P.2, B.1.1.33, B.1.1.28

## Materials and methods

### Search strategy

Four databases, including PubMed, Medline OVID, Web of Science, and CAB Direct, were searched systematically in June 2021, September 2021, and May 2022 using the search string described in Table 3. Searches were completed to identify all available peer-reviewed literature published on the efficacy of SARS-CoV-2 vaccines developed by Pfizer, Moderna, and AstraZeneca. In June 2021, 621 publications were identified, 323 of which were duplicates, leaving 298 distinct publications to be further screened. In September 2021, 1,228 publications were identified, 970 of which were duplicates, leaving an additional 258 distinct publications to be further screened. In May 2022, 4,511 publications were identified, 3,153 of which were duplicates, leaving an additional 1,358 publications to be screened.

**Table 3:** Search string used to identify publications from each of the four databases used for potential inclusion in the study.

**Search string**
“‘vaccin^*^ OR innoculat^*^ OR immunization^*^' AND ‘Pfizer OR AstraZeneca OR Moderna OR pfizer-biontech OR pfizerbiontech OR bnt162b2 OR mrna-1273 OR ChAdOx1-S OR Oxford AstraZeneca' AND ‘efficacy OR effective^*^' AND ‘COVID-19 OR SARS-CoV-2 OR COVID19^*^ OR COVID OR SARSCOV-2 OR SARSCOV2 OR Severe Acute Respiratory Syndrome Coronavirus 2 OR Severe Acute Respiratory Syndrome Corona Virus 2′”.

### Selection criteria and data extraction

To be included in this review, publications were required to meet all inclusion criteria and none of the exclusion criteria (Table 4). Two authors (LR and GA) independently reviewed titles and abstracts (step 1) and full-text publications (step 2) for inclusion in this review. Reviewers met to settle conflicts collaboratively. When publications were reported on both the mRNA vaccine(s) and the AstraZeneca vaccine, these publications were recorded as separate studies, resulting in a discrepancy between the number of publications and studies reported in the results section. Studies with fewer than 100 participants were not included in this review. For each study included, VE was extracted and described.

**Table 4:** Inclusion and exclusion criteria.

Inclusion criteria	• Peer-reviewed, human studies on a population level that included at least 100 participants • Accessible in English • Examining the efficacy of Pfizer, Moderna, or AstraZeneca vaccines against any level of infection or clinical signs and symptoms.
Exclusion criteria	• Systematic reviews, commentary articles, or gray literature, • Reporting on research on vaccines that were not Pfizer, Moderna, or AstraZeneca • Reporting on outcomes limited to antibody levels from blood tests/serology, safety of SARS-CoV-2 vaccines, side effects/adverse effects occurring from the vaccine, use of non-standard doses of vaccines, or VE determined without non-vaccinated controls. • Reporting on mathematical models, simulation, or emulation trials of VE • Studies limited to people with severe illness or conditions leading to reduced immune function

If a study determined VE over a range of days after the first or after the second dose, the VE at 14+ days post-vaccination was used when available, and if that was not available, we used the closest VE reported after 14 days. If that information was unavailable, we reported on VE at the latest time reported prior to 14 days after vaccination. This time interval was chosen as this was the most common reporting time for all studies on SARS-CoV-2 vaccines. The decline in VE over time was determined by comparing the 95% CI of VE of the first reported time interval to the 95% CI of VE at later intervals.

The VE for severe infection included those reported for severe/fatal/critical clinical disease, hospitalization, and death. To report on severe infection, the average VE was calculated for the separate values for various types of severe disease. The VE for mRNA vaccines that had separate results for Pfizer and Moderna was recorded as the average VE of these values. The variant in a study population was recorded as N/R for studies that did not specifically identify the variant infecting participants with a positive test. Studies that reported that variants were non-Delta, non-Alpha, and non-Beta were also recorded as N/R. Studies that reported mild to moderate infections were recorded as symptomatic infections. If a study reported separate VE values for the three symptomatic outcomes (mild, moderate, and symptomatic), these VE values were averaged and reported as one value for the symptomatic outcome. If studies reported a VE for both unidentified variants and variants that were not listed as known variants of concern, these two VEs were averaged and reported as the VE for unidentified variants. Most studies reported VE for all age groups in addition to specific age ranges. However, for those studies that only reported VE for specific age ranges, the average VE across all ages was reported.

### Clinical outcomes

PCR-positive infections were defined as any positive PCR test reported, regardless of symptoms. Asymptomatic COVID-19 infections were defined as a positive PCR test with no reported clinical signs or symptoms of COVID-19. Mild to moderate COVID-19 infections were defined by a positive PCR test and two or more of the following clinical signs and symptoms: fever, new-onset cough, new-onset rapid breathing, myalgia, new-onset shortness of breath with or without exertion, chills, sore throat, loss of taste, loss of smell, headache, nasal congestion, diarrhea, runny nose, tiredness, nausea or vomiting, loss of appetite, and any evidence of significant lower respiratory tract infection (Tachypnea: 20–29 breaths per minute at rest, oxygen saturation of 94% or less on room air, abnormal chest x-ray/CT consistent with pneumonia or lower respiratory tract infection, or adventitious sounds on lung auscultation). Symptomatic COVID-19 infections were defined by a positive PCR test and one or more of the following clinical signs and symptoms: fever (temperature >37.6°C), cough, sore throat, shortness of breath, muscle pain/myalgia, loss of smell/anosmia or loss of taste/ageusia, loss of appetite, nasal congestion, headache, nausea and vomiting, anorexia, dizziness, agitation, weakness, seizures, or findings suggestive of stroke, including trouble with speech or vision, sensory loss, or problems with balance in standing or walking, chills, diarrhea, chest pain, hoarseness, runny nose, sneezing, stomach pain, sinus pain, sweating, swollen glands, watery eyes, malaise/fatigue, or pneumonia. Severe/critical COVID-19 infections were defined by a positive PCR test and one or more of the following clinical signs and symptoms: clinical signs of pneumonia including fever, cough, dyspnea, fast breathing, and respiratory rate of 30 or more breaths per minute; heart rate at or exceeding 125 beats per minute; oxygen saturation at 93% or less, while the participant was breathing ambient air at sea level or a ratio of the partial pressure of oxygen to the fraction of inspired oxygen below 300 mm Hg; respiratory failure; acute respiratory distress syndrome; evidence of shock (systolic blood pressure <90 mm Hg, diastolic blood pressure <60 mm Hg, or a need for vasopressors); clinically significant acute renal, hepatic, or neurologic dysfunction; sepsis, septic shock, acute thrombosis, MIS-C, requiring mechanical ventilation, adventitious sounds on lung auscultation, admission to an intensive care unit, or death.

## Results

### Description of the publications

**Figure 1:**
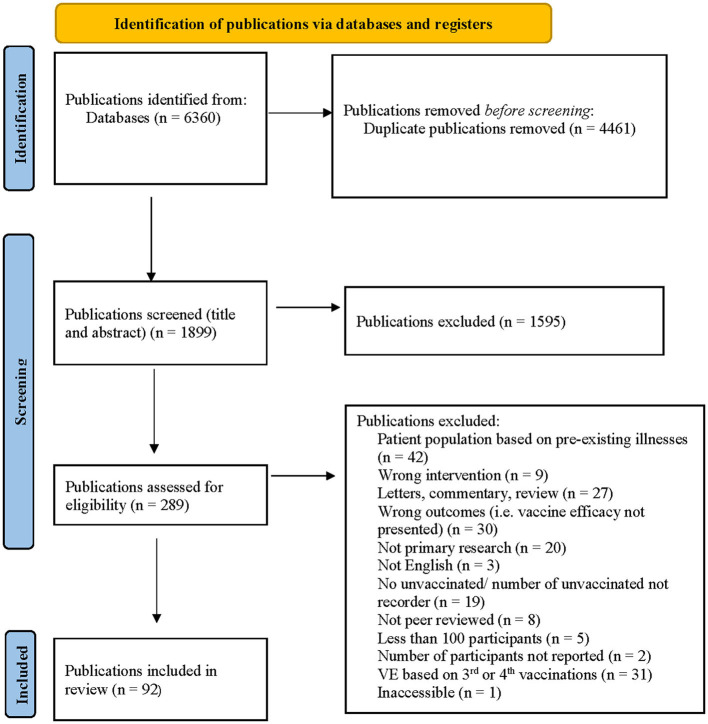
Prisma flow chart for the search and selection of publications.

After applying the inclusion and exclusion criteria to the 298 unique publications identified in June 2021, 263 publications were excluded by title and abstract screening. Fourteen additional publications were excluded after full-text screening, leaving 21 publications for inclusion in the review. After applying the inclusion and exclusion criteria and screening procedures to the 258 unique publications identified in September, an additional 12 publications were included in the review. After applying the inclusion and exclusion criteria and screening procedures to the 1,358 new publications identified in May 2022, an additional 59 publications were included in the review (Figure 1).

In total, 92 publications were included in this systematic review, which included a total of 114 studies (Supplementary Tables 1, 2). Some included data from more than one country (*n* = 4) (67, 82, 87, 95). Most studies were conducted in the UK (*n* = 33) (10, 13, 14, 16, 18, 24, 42, 43, 45, 49, 50, 53, 54, 56, 65, 69, 71, 85, 94, 97) or the USA (*n* = 34) (9, 12, 17, 21, 22, 26, 28, 32, 33, 36, 37, 46, 48, 52, 55, 59, 60, 64, 66, 67, 70, 72, 73, 79–84, 87–89, 91, 95). Of the studies reporting in the UK, 14 were conducted specifically in England (10, 13, 14, 24, 42, 49, 50, 53), 5 in Scotland (54, 85, 97), and 3 in Wales (*n* = 3) (18, 65). Other studies were in Israel (*n* = 9) (15, 29, 30, 39–41, 57, 74, 92), Italy (*n* = 8) (11, 19, 20, 34, 35, 38, 63)), Brazil (*n* = 6) (23, 67, 82, 93, 96), Malaysia (*n* = 4) (44, 76, 77), Spain (*n* = 4) (31, 61, 87), Finland (*n* = 3) (68, 87), South Africa (*n* = 3) (67, 82, 98), Canada (*n* = 3) (27, 75, 90), France (*n* = 2) (62), Argentina (*n* = 2) (67, 82), Germany (*n* = 2) (67, 82), Qatar (*n* = 2) (25, 78), Sweden (*n* = 2) (58), Hungary (*n* = 2) (86), Turkey (*n* = 2) (67, 82), Kuwait (*n* = 2) (8), South Korea (*n* = 1) (47), Poland (*n* = 1) (87), Thailand (*n* = 1) (99), Peru (*n* = 1) (95), Chile (*n* = 1) (95), and Japan (*n* = 1) (51).

Data collection for the studies occurred between May 2020 and February 2022. Most studies investigated the VE of one of the mRNA vaccines (*n* = 85) (8–79, 81–92), whereas 29 (8, 10, 13, 14, 23, 24, 31, 35, 43, 49, 50, 54, 56, 58, 62, 65, 68, 69, 71, 76, 85, 86, 93–99) examined the AstraZeneca vaccine (Supplementary Tables 1, 2). In total, 13 (8, 16, 31, 40, 45, 49, 53, 56, 62, 75, 85) studies determined VE after the first dose only, 36 (11, 15, 24, 28, 32, 35, 36, 44, 48, 52, 54, 55, 57, 58, 60, 63, 65, 70, 72–74, 77, 80, 86–89, 93–95, 99) determined VE after the second dose only, and 65 (8–10, 12–14, 17–23, 25–27, 29–31, 33–35, 37–39, 41–43, 46, 47, 49–51, 59, 61, 62, 64, 66–69, 71, 76, 78, 79, 81–84, 90–92, 96–98) determined VE after both the first and second doses.

### Time post-vaccination

The timing of follow-up post-vaccination differed; some authors (*n* = 30) (15, 19, 25–27, 29–31, 34, 37, 38, 41, 42, 46, 61, 64–67, 71, 79, 80, 82, 86, 87, 90, 92) determined VE beginning 7 days post-vaccination after the first or second dose, whereas others (*n* = 62) (8–10, 16, 17, 20, 21, 23, 24, 28, 32, 33, 35, 36, 39, 43–45, 47, 48, 50–52, 54, 55, 57–60, 62, 63, 68–70, 72, 73, 76–78, 81, 83–85, 88, 91, 94, 96, 98, 99) followed participants beginning 14 or more days after receiving the first or second dose of the vaccine. Twenty-two studies (8, 11–14, 18, 22, 31, 40, 49, 53, 56, 74, 75, 89, 93, 95, 97) used an alternative follow-up period starting from 0 days to 10 months after vaccination. If VE was reported 0–7 days or 0–14 days after vaccination, these results were not recorded in Supplementary Tables 1, 2. Many studies (*n* = 54) (10, 12–14, 18–20, 22, 25–27, 29–31, 34, 37–39, 41–43, 46, 49, 50, 53, 54, 61, 64, 66–69, 71, 79, 80, 82, 85, 86, 90, 92, 96, 97) determined VE at multiple time intervals.

### Type of study

The study designs included randomized controlled trials, observational studies of prospective and retrospective cohorts, and case–control studies. Twelve studies used a blinded, randomized, controlled clinical trial (9, 17, 33, 37, 38, 67, 82, 87, 93–95, 98). Most studies (*n* = 58) used an observational cohort approach that followed vaccinated and unvaccinated people over the same time period. Cohort studies were divided between prospective (*n* = 32) (12, 18, 28, 29, 31, 36, 39–43, 53, 55–57, 62, 68, 69, 71, 72, 85, 86, 90) and retrospective (*n* = 26) (8, 11, 15, 16, 19, 20, 26, 30, 34, 35, 47, 58, 63–65, 73, 74, 76, 77, 79, 92) cohort study designs. In case–control studies (*n* = 44) (10, 13, 14, 21–25, 27, 32, 44–46, 48–52, 54, 59–61, 66, 70, 75, 78, 80, 81, 83, 84, 88, 89, 91, 96, 97, 99), patients with a positive PCR test with or without clinical signs, and symptoms of COVID-19 were matched by age with people with a negative PCR test during the same time period.

### Populations included in the studies

The number of participants in the randomized controlled trials ranged from 1,882 to 46,077, with a median of 9,636. Studies were conducted on populations of different age ranges, including 12+ (*n* = 1) (82), 16+ (*n* = 1) (67), 18–65 (*n* = 1) (98), and 18+ (*n* = 6) (17, 33, 38, 93–95). Three of these studies were limited to youth: 5–11 years (87), 12–15 years (37), and 12–17 years (9), respectively. One study was on individuals aged 12+ (82), one was 16+ (67), one was 18–65 (98), and six were 18+ (17, 33, 38, 93–95). Most studies (*n* = 10) (9, 17, 33, 37, 67, 82, 87, 93, 95, 98) included participants randomly selected from the population, whereas two (38, 94) only included healthcare workers.

The number of participants in the prospective cohort studies ranged from 1,023 to 6,538,911 with a median sample size of 407,994 The source population or source of data for these observational studies included data extracted by a local health provider (*n* = 5) (12, 31, 55, 72), local health database (*n* = 1) (36), national healthcare provider (*n* = 1) (29), national health database (*n* = 8) (39–41, 53, 85, 86), healthcare workers (*n* = 9) (8, 18, 43, 62, 68, 90), a self-reported survey (*n* = 7) (42, 56, 57, 69, 71), and veterans' health administration (*n* = 1) (28).

The number of participants in the retrospective cohort studies ranged from 1,610 to 5,693,624 with a median sample size of 398,593. The source population or source of data for these observational studies included local health databases (*n* = 3) (64, 73, 79), local healthcare providers (*n* = 2) (30, 74), national health databases (*n* = 11) (11, 35, 58, 65, 76, 77, 92), healthcare workers (*n* = 8) (8, 15, 16, 19, 20, 34, 63), prison healthcare records (*n* = 1) (26), and ministry of education health and immunization records (*n* = 1) (47).

The number of participants in the case–control studies ranged from 404 to 6,647,733 with a median sample size of 96,726. The source population or source of data for these observational studies included local healthcare providers (*n* = 1) (78), local health databases (*n* = 8) (21, 27, 59, 61, 75, 80, 84, 88), national healthcare providers (*n* = 3) (24, 45), national health databases (*n* = 7) (23, 25, 54, 96, 97), healthcare workers (*n* = 1) (66), military personnel health records (*n* = 1) (32), hospital records (*n* = 9) (48, 51, 52, 60, 70, 81, 83, 89, 99), national immunization system (*n* = 10) (10, 13, 14, 49, 50), ministry of education health and immunization records (*n* = 1) (44), and veterans' health administration records (*n* = 3) (22, 46, 91). Amirthalingam (2021) reported multiple accounts of VE after the second dose, depending on various time intervals between doses 1 and 2. Supplementary Tables 1, 2 include the authorized interval between doses 1 and 2 as reported by Public Health Canada (100).

### Outcomes of interest

Reported outcomes included asymptomatic infection (*n* = 12) (9, 12, 15, 29, 32, 33, 41, 59, 69, 78, 94), PCR-positive tests (*n* = 77) (10–12, 16, 18–22, 24–36, 38–44, 46, 47, 53, 55–59, 61–66, 68, 69, 71– 75, 77–79, 82, 84, 86–88, 90–92, 94, 95, 98, 99), symptomatic infection (*n* = 43) (8, 9, 12–15, 17, 20, 23, 24, 27, 29, 30, 32–35, 37, 38, 41, 49–51, 55, 59, 67, 69, 78, 91, 93–98), mild/moderate symptomatic infection (*n* = 1) (98), severe/critical disease (*n* = 15) (11, 17, 25, 29, 33, 39, 41, 54, 67, 70, 78, 82, 93, 95), hospitalization (*n* = 47) (11, 14, 21, 23, 27, 29, 30, 32, 36, 39, 41, 45, 48, 52–54, 60, 61, 64, 65, 68, 70, 72–74, 76, 77, 79–81, 83–85, 88, 89, 91, 93, 95–97), and death (*n* = 25) (11, 14, 23, 25, 27, 29, 33, 35, 36, 39–41, 46, 61, 74, 76–78, 86, 91, 96, 97) (Table 4 and Supplementary Table 1). Commonly, severe illness, hospitalization, and death were included together. Fifty-four studies (13, 14, 21, 23, 25, 27–30, 32, 35, 36, 41, 43, 48–50, 55, 59, 62, 65, 68–73, 75, 77–82, 84, 86, 90, 93, 94, 96–99) reported VE against specific variants (Table 4 and Supplementary Table 1) and 99 (8–12, 14–24, 26, 28–41, 43–47, 49, 51–68, 71–77, 79, 81–83, 85–99) used the term ‘unknown' (N/A) if additional laboratory tests were not done or did not identify a known variant.

### Vaccine efficacy

The median VE for all vaccines was numerically highest for the clinical outcomes such as severe illness, hospitalization or death, and symptomatic infection measured at approximately 14 days after a second dose of the vaccine (Table 5). Supplementary Table 3 describes the VE by age. Thirty-six studies (10, 14, 21–23, 27–29, 33, 36, 39, 46, 48, 60, 61, 66, 67, 69, 75, 77–82, 85, 86, 91, 95, 97) reported VE for various age groups. Supplementary Table 4 describes the VE over time. Thirty-four studies (13, 14, 18, 20, 21, 23, 27, 36, 43, 48, 50, 51, 54, 56, 58, 59, 68–70, 75, 77, 79, 80, 82, 84, 85, 89, 97) reported VE over multiple time intervals. This table includes any additional time intervals not reported in Supplementary Tables 1, 2.

**Table 5:** Distribution of vaccine efficacy against SARS-CoV-2 by vaccine type, outcome, and variant.

		**One Dose mRNA**				**Two doses mRNA**			
**Outcome**	**Variant**	**n** ^a^	**Min**	**Median**	**Max**	**n** ^a^	**Min**	**Median**	**Max**
PCR positive	ALPHA	7	30	67	90.1	6	76.4	91.7	100
	BETA	1	-	47.9	-	1	-	96.4	-
	DELTA	6	56	62.3	77	12	50.7	74.7	86.7
	EPLISON	1	-	76.3	-	1	-	97.6	-
	IOTA	-	-	-	-	1	-	95.7	-
	MU	-	-	-	-	1	-	90.4	-
	N/R	36	31	64.5	97.7	51	50.1	89	99.1
	OMICRON	1	-	20.4	-	1	-	13.9	-
	ORIGIN	1	-	53	-	1	-	94.9	-
	P.1/P.1.1/P.1.2	2	61	-	74.2	1	-	95.5	-
Symptomatic	ALPHA	3	47.5	52	73	3	93.7	94.9	97
	DELTA	5	35.6	64.9	68	6	49.2	87	92.7
	N/R	17	45	66	99.2	23	58	93.2	100
	OMICRON	1	-	31.7	-	1	-	70.3	-
Asymptomatic	DELTA	2	47	-	62.5	3	45.9	71.5	74
	N/R	3	29	52	59.5	8	39.2	85.5	93.8
Severe, hospitalization, and death	ALPHA	2	67.7	-	87.2	4	85	92.9	96.7
	DELTA	4	71.2	85.1	100	12	68.5	94.2	100
	N/R	19	41.2	69.3	100	36	73.8	92.2	100
	OMICRON	-	-	-	-	4	54.5	69.3	84.5
		**One Dose AZ**				**Two doses AZ**			
**Outcome**	**Variant**	**n** ^a^	**Min**	**Median**	**Max**	**n** ^a^	**Min**	**Median**	**Max**
PCR positive	ALPHA	2	56	-	63	2	61.7	-	79
	BETA	1	-	30	-	1	-	19.2	-
	DELTA	2	43	-	100	2	67	-	88
	N/R	8	7	53.4	86.2	12	24.9	69.6	95
	ORIGIN	1	-	91	-	-	-	-	-
Symptomatic	ALPHA	3	45.1	48.7	73	4	70.4	78.5	97
	BETA	1	-	36.8	-	1	-	9.6	-
	DELTA	4	30	39.5	42.9	4	67	68.8	82.8
	N/R	6	17.8	48.8	75.4	8	45.5	71	100
	OMICRON	1	-	17.7	-	1	-	48.9	-
	P.1	-	-	-	-	1	-	63.6	-
	P.2	-	-	-	-	1	-	68.7	-
	B.1.1.28	-	-	-	-	1	-	72.6	-
	B.1.1.33	-	-	-	-	1	-	88.2	-
Symptomatic	ALPHA	-	-	-	-	1	-	28.9	-
	DELTA	1	-	50	-	1	-	57	-
	N/R	-	-	-	-	1	-	69.7	-
Severe, hospitalization, and death	ALPHA	1	-	85.5	-	1	-	95.1	-
	DELTA	2	83.8	-	100	2	95.1	-	100
	N/R	6	−15	53.1	73	10	81.4	90.2	100

^a^n represents the number of studies reporting results for this vaccine–outcome–variant combination. mRNA, Pfizer BioNTech or Moderna; AZ, Astra Zeneca.

Vaccines were less efficacious against asymptomatic cases, PCR-positive cases, or mild to moderate symptoms than against severe infections (Table 5). The median VE against unidentified variants for PCR-positive infections, asymptomatic infections, symptomatic infections, and severe infections after two doses of an mRNA vaccine were 89, 85.5, 93.2, and 92.2%, respectively. The median VE against unidentified variants for PCR-positive infections, asymptomatic infections, symptomatic infections, and severe infections after two doses of the AstraZeneca vaccine was 69.6, 69.7, 71, and 90.2%, respectively.

Of the 36 (10, 12, 18–22, 25, 26, 29–31, 34, 35, 38, 39, 41–43, 46, 47, 59, 61, 62, 64, 66, 68, 69, 71, 78, 79, 82, 84, 90–92) studies that reported VE for PCR-positive infection after the first and second doses of mRNA vaccines, 24 found an increased VE after the second dose (Supplementary Tables 1, 2) (10, 19, 21, 22, 25, 26, 29–31, 39, 41, 43, 46, 47, 61, 64, 66, 68, 69, 78, 79, 90–92). Of the 6 (10, 43, 68, 69, 71, 98) studies that reported VE for PCR-positive infection after the first and second doses of AstraZeneca, 3 (10, 68, 69) found increased VE after the second dose (Supplementary Tables 1, 2). The median VE for all vaccines was numerically lower against unidentified variants after the first dose than after the second dose (Table 5). The median VE for PCR-positive infections from an mRNA vaccine was 64.5 and 89% after one or two doses against unidentified variants, respectively. The median VE for PCR-positive infections from the AstraZeneca vaccine was 53.4 and 69.6%, respectively, after one or two doses against unidentified variants. Vaccine efficacy <14 days after the first dose of an mRNA vaccine was lower than VE determined at least 14 days after the first dose (26, 66) (Supplementary Tables 1, 2, 4).

Most studies that identified variants determined VE for either the Alpha or Delta variants (Table 5) (13, 14, 21, 25, 27–30, 32, 35, 36, 41, 42, 48–50, 55, 59, 62, 65, 68–73, 75, 77–81, 84, 86, 90, 94, 97, 99). The median VE after the second dose of an mRNA vaccine was numerically lower for Delta (VE = 74.7%) compared to Alpha (VE = 91.7%) for PCR-positive cases. The median VE after the second dose of an mRNA vaccine was numerically lower for Delta (VE = 87%) compared to Alpha (VE = 94.9%) for symptomatic infections. The median VE after the second dose of an mRNA was numerically higher for Delta (VE = 94.2%) compared to Alpha (VE = 92.9%) for severe infections. The median VE against the Omicron variant after two doses of the mRNA vaccines against severe infections was 69.3% (13, 48, 70, 80, 84). This was numerically lower than the Delta and Alpha efficacy for this outcome, as reported above. After the second dose of the AstraZeneca vaccine, the median VE for positive PCR infections against the Delta variant was 77.5% compared to 70.4% against the Alpha variant. The median VE after the second dose of the AstraZeneca vaccine for severe infection against the Delta variant was 97.6 and 95.1% against the Alpha variant.

Many studies (*n* = 30) reported the VE across various age ranges separated within the study population (10, 14, 21–23, 27–29, 33, 36, 39, 46, 48, 60, 61, 66, 67, 69, 75, 77–82, 85, 86, 91, 95, 97). Most of these studies reported VE 14 days after either dose. The VE did not differ by the age of the person in any of the included studies (Supplementary Table 4).

A few studies (*n* = 6) compared the VE by previous exposure to SARS-CoV-2 as measured by persons who were previously infected or not previously infected (17, 42, 43, 69, 98) (Supplementary Tables 1, 2). The results differed by study. In three studies, VE did not differ due to previous infections (17, 42, 98), whereas three other studies reported that prior infections led to an increased VE for the first and second doses of either vaccine (43, 69).

Vaccine efficacy was highest between 2 and 10 weeks after the second dose of the vaccine (Supplementary Tables 1, 2, 4). Vaccine efficacy decreased over time following this initial period. The median time when VE began to decline for mRNA vaccines was 10–14 weeks (13, 14, 69), 13–18 weeks (21, 36, 43, 58, 68, 77, 79, 82, 84), and 13–16 weeks (14, 77, 80, 89) for symptomatic, PCR-positive, and severe illness, respectively. The median time when VE declined for the AstraZeneca vaccine was 10–14 weeks (13, 14), 8–17 weeks (58), and 10–14 weeks (14, 97) for symptomatic, PCR-positive, and severe illness, respectively.

## Discussion

Studies included in this systematic review indicated that the Pfizer, Moderna, and AstraZeneca vaccines were efficacious against asymptomatic, mild, moderate, and severe COVID-19, especially following two doses. Vaccine efficacy was highest against severe infections, including hospitalization and death. Reported VE was dependent on the variant of concern during the time of the study, the number of doses included during the reporting period (i.e., one vs. two doses), and the specific outcomes measured.

### Vaccine efficacy by the outcome

For all vaccines, VE was higher against severe infections than mild or moderate symptomatic infections and asymptomatic infections. Studies reported on several clinical outcomes, including any PCR-positive infection, asymptomatic infections, symptomatic mild-moderate infections, severe/critical infections, hospitalizations, and deaths. Vaccine efficacy against severe infection in this study included severe infection, hospitalization, and death. Vaccine efficacy differed by clinical outcome. The median VE reported in the current study for two doses of mRNA and AstraZeneca was 85.5 and 69.7%, respectively, for asymptomatic infections, and 92.2 and 90.2% for severe infections, respectively.

### SARS-CoV-2 variants

Vaccine efficacy is affected by the dominant variant of SARS-CoV-2 in the region and time period in which a study is conducted. The Alpha variant is 40–80% more transmissible than the origin strain of SARS-CoV-2, leading to higher infection rates (101). Furthermore, the Alpha variant led to more severe clinical signs and symptoms, including a 55% increase in mortality compared to previous variants (101). The VE for AstraZeneca is lower for the Beta variant than for the origin strain (101). Furthermore, serum from individuals vaccinated with either mRNA vaccine had lower neutralizing antibody titers against the Beta variant than against the origin strains (101). The Beta variant elicited improved entry and binding to target cells in the body over the origin strain, thus resulting in a higher infection rate (102). The P.1 variant recorded similar mutations as the Beta variant, thus resulting in an assumed similar VE (101). Regarding the Delta variant, studies reported a 2.5-fold decrease in neutralization using serum from individuals vaccinated with Pfizer, compared to the original SARS-CoV-2 variant, while neutralization by serum from the AstraZeneca vaccine was 4.3-fold lower compared to the original SARS-CoV-2 variant (103). This reduced neutralization illustrates a possible reason for the reduced VE against the Delta variant and may explain the increased breakthrough infections that have been reported in several countries when the Delta variant became most dominant (103). The VE for PCR-positive infection after two doses of Moderna was reported as 98.4% for Alpha and 86.7% for Delta (21). The VE for symptomatic infection after two doses of Pfizer was reported as 94.9% for Alpha and 83.3% for Delta (14). The VE for symptomatic infection after two doses of AstraZeneca was reported as 82.1% for Alpha and 64.2% for Delta (14). Thus, the results demonstrate how the VE of these vaccines changes depending on the variant circulating in the study population.

### Time after vaccination

Vaccine efficacy is dependent on the time after vaccine administration. In many studies within this review, VE was determined at least 14 days after the first dose (8–10, 16, 17, 19–21, 23, 25–27, 29–31, 33–35, 38, 41, 45–47, 51, 59, 61, 62, 64, 66, 76, 78, 79, 81, 83–85, 90–92, 96–98). However, various studies have suggested that the vaccine effect of one dose can numerically increase for up to 21 days (19, 25, 29–31, 43, 50, 53, 56, 68, 69, 71, 75, 85) or 28 days after the first dose (8, 10, 14, 46, 49, 85, 96). Additionally, many studies reported VE at least 7 days after the second dose (14, 15, 19, 22, 25, 27, 29–31, 34, 37, 38, 41, 42, 46, 61, 64–67, 71, 79, 80, 82, 86, 87, 90, 92). Although various studies suggested that vaccine effects can numerically increase for up to 14 days (8–10, 13, 14, 17, 18, 20, 21, 23–26, 28, 31–33, 35, 36, 39, 41, 43, 44, 47–52, 54, 55, 57–64, 68–70, 72, 73, 76–78, 81, 83, 84, 86, 88, 91, 94, 96–99), or 28 days after second vaccination this is not substantiated by studies measuring antibiodies (86). Antibody studies showed that among individuals who had no previous infections, IgG antibody levels were maximized approximately 4 weeks after the first dose and 2 weeks after the second dose for mRNA vaccines (104–106). Similarly, neutralizing antibodies were maximized approximately 28 days after the first dose and 14 days after the second dose of the AstraZeneca vaccine (107, 108). Therefore, in any future, efficacy research focused on these vaccines, outcomes should be recorded during this period to determine the maximum possible VE.

Over a longer period after the second dose, VE declined. The median time when VE declined after two doses of the mRNA vaccines and AstraZeneca was 13–18 weeks (21, 36, 43, 58, 68, 77, 79, 82, 84) and 8–17 weeks (58) for PCR-positive infections, respectively.

## Limitations

Many studies controlled for several co-variates such as age, sex, socio-economic status, geographical source, and pre-existing illnesses. However, other studies did not control for any co-variates or only controlled for a few co-variates, such as age and sex. These analytical inconsistencies may have reduced the comparability of VE and increased the variability of VE reported across the studies.

Publications were included in this report if they reported population-level data within specific age groups and geographic areas. This review excluded publications that focused on specific pre-existing immunocompromised-related conditions such as multiple sclerosis and cancer. Immunocompromised individuals were included in the broad population, yet vaccine efficacy specific to these cohorts was not determined. Therefore, the vaccine efficacy results cannot be generalized to individuals who are immunocompromised.

Many individuals across Canada have received more than two doses of a SARS-CoV-2 vaccine (109). Research on VE in populations receiving a third or fourth dose of the vaccine was beyond the scope of this systematic review. Additionally, many Canadians were vaccinated with a combination of available vaccines (109). Similarly, the VE has been determined for persons receiving a combination of different vaccines for their first and second SARS-CoV-2 vaccinations. For example, the initial vaccine may have been AstraZeneca, followed by a second dose using an mRNA vaccine. The VE for heterologous vaccination protocols was not included in the current study. Therefore, the findings of the current study may not apply to either third booster doses or VE for heterologous vaccination programs, and further research is required to determine the VE of heterologous and booster vaccines.

The results of this study cannot be generalized to new and emerging variants of SARS-CoV-2. In May 2021, the Delta variant was determined to be the variant of concern within North America, yet by November 2021, the Omicron variant was identified and was still the variant of concern in North America 1 year later (110). The database searches were completed in May 2022, and consequently only included six studies reporting on the efficacy of the Omicron variant. Therefore, we cannot conclude the efficacy of vaccines against Omicron or any future variants. Nonetheless, because of emerging new variants of concern such as Omicron, ongoing research is required to determine the effectiveness of mRNA and AstraZeneca vaccines. More recently, bivalent mRNA vaccines specifically targeted against the Omicron variants have been approved for use in Canada (111, 112). This review does not include VE for these bivalent vaccines.

The results of this study present estimates of the efficacy of several SARS-CoV-2 vaccines, including Pfizer, Moderna, and AstraZeneca vaccines, based on population-level studies. After 14 days, a second dose of any of the vaccines represented in this study provided highly efficacious protection against severe infections, hospitalization, or death due to SARS-CoV-2 infections. VE still provided good protection against other outcomes such as mild-moderate symptomatic infection, asymptomatic infection, and any PCR-positive infection, suggesting that these vaccines can protect against any severity of infection due to SARS-CoV-2. In both mRNA and AstraZeneca vaccines, efficacy was highest after the second dose, against the original strains of SARS-CoV-2, and when the time reporting clinical signs and symptoms was at least 14 days after vaccination. As strains continued to evolve from the origin, VE decreased. Furthermore, these results illustrated that VE wanes over time after the second dose, suggesting that although two doses provide adequate protection, this does not remain indefinitely. Nonetheless, whether reporting on PCR-positive infection, mild, moderate, or asymptomatic infection, all the vaccines included in this systematic review provided good protection against SARS-CoV-2, while these same vaccines provided excellent protection against severe illness, hospitalization, and death.

## Data availability statement

The original contributions presented in the study are included in the article/Supplementary material, further inquiries can be directed to the corresponding author.

## Author contributions

LR conducted the literature search, identified manuscripts for inclusion, and wrote the first draft and edited versions of the manuscript. CD is the senior author who contributed to the concept and design of the study, mentored the first author, and wrote sections of the manuscript. GA was the second reviewer of the manuscripts for inclusion. ML contributed to the design of the manuscript, critically evaluated the analysis, and provided clarity to the final draft. All authors contributed to manuscript revision, read, and approved the submitted version.
