# Supplementary material for: Vaccine efficacy against SARS-CoV-2 for Pfizer BioNTech, Moderna, and AstraZeneca vaccines: a systematic review
Source: Front Public Health. 2023 Oct 24;11:1229716. doi: 10.3389/fpubh.2023.1229716 (PMC10628441; doi:10.3389/fpubh.2023.1229716)
Supplement: Supplementary file 1 [file Table_1.pdf]

Supplementary Table 1. Population-level efficacy of vaccination with either Pfizer-BioNTech (BNT162b2) (Pfizer)/ Moderna (mRNA-1273) vaccines against test positivity, clinical signs, hospitalization, or death due to SARS-CoV-2.

**Study (Publication Year) Country (Time of data completion)**	**Study design**	**Study Population (n)**	**Variant of interest**	**Vaccine**	**Outcome(s) measured**	**Adjusted vaccine efficacy (95% CI)**	
						**After dose 1**<br>(Vaccine) time following vaccination: variant = efficacy (95% confidence interval)	**After dose 2**<br>(Vaccine) time following vaccination: variant = efficacy (95% confidence interval)
W.Q. Alali (2021) *Kuwait* (12/20-06/21)	Retrospective cohort study	Healthcare workers (HCWs) ages 20+. (1,029 vaccinated and 581 unvaccinated)	N/A	Pfizer	Symptomatic infection	14+ d: 91.4% (65.1-97.9)	14+ d: 94.5% (89.4-97.2)
K. Ali (2021) USA (12/20- 02/21)	Phase 2-3 placebo-controlled trial	Individuals 12-17 years old. (2,489 vaccinated and 1,243 placebo)	N/A	Moderna	PCR test-positive infection + 1 Symptom<br><br>PCR test-positive infection + at least 2 symptoms<br><br>Asymptomatic infection	14+ d: 92.7% (67.8-99.2)<br><br>14+ d: 69.8% (49.9-82.1)<br><br>14+ d: 59.5% (28.4-77.3)	14+ d: 93.3% (47.9-99.9)<br><br>14+ d: 55.7% (16.8-76.4)<br><br>14+ d: 39.2% (-24.7-69.7)
G. Amirthalingam (2021) England (10/20-06/21)	**Test-negative, case-control study**	Individuals aged 65-79 in the National health service and national immunisation management system. (225 received 1 dose, 211 received 2 doses, and 103,284 unvaccinated)	N/A	Pfizer	PCR test-positive infection	14-27 d: 43% (37-48)<br>28+ d: 53% (48-58)l	14+ d: [19-29 d between dose]= 77% (66-85)
E. Amodio (2022) Italy (01/21-09/21)	**Retrospective cohort study. Outcomes measured without time following vaccination.**	Individuals aged 18+ in Sicily with records in National health registries. (2,469,320 vaccinated with two doses and 1,497,656 unvaccinated)	N/A	Pfizer and Moderna	PCR test-positive infection<br><br>Severe infection<br><br>Death/ hospitalization	Not reported	Feb-Sept: 57.8% (55.4-60.2)<br><br>Feb-Sept: 90.3% (86.2-94.4)<br><br>Feb-Sept: 83.7% (75.1-92.3)
K.L. Andrejko (2021); USA (02/21-04/21)	**Survey, Prospective cohort study**	Individuals 18+ with records in the California	N/A	Pfizer and Moderna	PCR test-positive infection	8-14 d: 50.7% (-17.5-79.8)<br>15+ d: 66.9% (28.7-84.6)	8-14 d: 79.4% (39-92.9)<br>15+ d: 87.4% (77.4-93.1)<br>(Pfizer) 15+ d: 87% (68.6-94.6)

		Department of Public Health (525 case: 51 received 1 dose, 20 received 2 doses and 454 unvaccinated. 498 controls: 99 received 1 dose, 86 received 2 doses, and 313 unvaccinated))			Symptomatic infection<br><br>Asymptomatic infection	Not reported<br><br>Not reported	(Moderna) 15+ d: 86.2% (68.4-93.9)<br><br>15+ d: 91.3% (79.3-96.3)<br><br>15+ d: 68.3% (27.9-85.7)
N. Andrews[A] (2022) England (11/21-01/22)	**Test-negative case-control**	Individuals 18+ years old with records in the National immunization system (676,720 received Pfizer, 77,203 received Moderna, and 244,716 unvaccinated)	B.1.617.2, B.1.1.529	Pfizer and Moderna	Symptomatic infection	(Pfizer) 0-3 wks: B.1.1.529= 42.8% (40.3-45.1)<br>(Pfizer) 4+ wks: B.1.1.529= 31.5% (29.9-33.1)<br>(Moderna) 0-3 wks: B.1.1.529= 47.9% (43.1-52.3)<br>(Moderna) 4+ wks: B.1.1.529= 31.9% (27.3-36.1)<br>(Pfizer) 0-3 wks: B.1.617.2= 45.2% (43.3-47.1)<br>(Pfizer) 4+ wks: B.1.617.2= 72.3% (69.4-74.9)<br>(Moderna) 0-3 wks: B.1.617.2=60.1% (51.8-66.9)<br>(Moderna) 4+ wks: B.1.617.2= 57.4% (52.6-61.8)	(Pfizer) 2-4 wks: B.1.1.529= 65.5% (63.9-67)<br><br>(Moderna) 2-4 wks: B.1.1.529= 75.1% (70.8-78.7)<br>(Pfizer) 2-4 wks: B.1.617.2= 90.9% (89.6-92)<br>(Moderna) 2-4 wks: B.1.617.2= 94.5% (90.5-96.9)
N. Andrews[B] (2022) England (12/20-10/21)	**Test-negative, case-control study**	Individuals ages 16+ across England with record in the National Immunization system. (2,527,194 vaccinated with Pfizer, 209,110 vaccinated with Moderna, and 855,743 unvaccinated.)	B.1.1.7, B.1.617.2, N/A	Pfizer and Moderna	Symptomatic infection<br><br>Hospitalization<br><br>Death	(Pfizer) 28+ d: B.1.617.2 = 51.2% (50.7-51.7)<br>(Pfizer) 28+ d: B.1.1.7= 45.9% (44.2-47.6)<br>(Moderna) 28+ d: B.1.617.2= 64.9% (64-65.7)<br>(Moderna) 28+ d: B.1.1.7 = 58.1% (11.7-80.1)<br><br>(Pfizer) 28+ d: B.1.617.2= 91.1% (89.7-92.3)<br>(Pfizer) 28+ d: B.1.1.7= 85.8% (82.2-88.7)<br>(Moderna) 28+ d: B.1.617.2= 93.7% (89.9-96)<br><br>(Pfizer) 28+ d: B.1.617.2= 88.6% (78.8-93.9)<br>(Pfizer) 28+ d: B.1.1.7= 88.5% (78.5-93.8)	(Pfizer) 1 wk: B.1.617.2 = 92.3% (92-92.6)<br>(Pfizer) 2-9 wk: B.1.617.2= 89.7% (89.5-89.8)<br>(Pfizer) 1 wk: B.1.1.7= 90.2% (86.9-92.7)<br>(Pfizer) 2-9 wk: B.1.1.7= 94.9% (93.6-95.9)<br>(Moderna) 1 wk: B.1.617.2= 95.6% (95-96.2)<br>(Moderna) 2-9 wk: B.1.617.2= 93.8% (93.4-94.1)<br><br>(Pfizer) 1 wk: B.1.617.2= 99.4% (97.7-99.9)<br>(Pfizer) 2-9 wk: B.1.617.2= 98.7% (98.3-99)<br>(Pfizer) 1 wk: B.1.1.7= 96.4% (74.2-99.5)<br>(Pfizer) 2-9 wk: B.1.1.7= 97.7% (90.8-99.4)<br>(Moderna) 1 wk: B.1.617.2= 97.9% (84.9-99.7)<br>(Moderna) 2-9 wk: B.1.617.2= 100% (N/A)<br><br>(Pfizer) 2-9 wk: B.1.617.2= 98.5% (96.5-99.3)<br>(Pfizer) 2+ wk: B.1.1.7= 95.6% (94.4-96.5)

Y Angel (2021) Israel. (12/20-02/21)	Retrospective cohort study	HCWs (416 received 1 dose, 5,517 received 2 doses and 757 unvaccinated)	N/A	Pfizer	Symptomatic infection<br><br>Asymptomatic infection	Not reported	7+ d: 97% no CI<br><br>7+ d: 86% no CI
T. Azamgarhi (2021) UK (01/21-03/21)	Retrospective cohort study	HCWs aged 16+ (1,409 vaccinated and 826 unvaccinated)	N/A	Pfizer	PCR test-positive infection	14+ d: 70% (6-91)	Not reported
L. Baden (2020) USA (06/20-11/20)	Randomized, stratified, observer-blinded, placebo-controlled trial.	Individuals aged 18+ from 99 US sites. (608 received 1 dose, 14,602 received 2 doses, and 15,210 placebo)	N/A	Moderna	Symptomatic infection<br><br>Severe infection<br><br>Previously infected	14+ d: 95.2% (91.2-97.4)<br><br>Not reported	14+ d: 94.1% (89.3-96.8)<br><br>14+ d: 100% (N/A)<br><br>14+ d: 93.6% (88.6-96.5)
S. Bedston (2022) Wales (12/20-09/21)	Prospective cohort study	HCWs aged 16+. (2,840 vaccinated with 1 dose, 43,175 vaccinated with 2 doses, and 8,569 unvaccinated)	N/A	Pfizer	PCR test-positive infection	3-6 wk: 52% (N/A)	2 wk: 67% (N/A)<br>2-5 wk: 86% (N/A)
F.P. Bianchi[A] (2021) Southern Italy (12/20-01/21)	Retrospective cohort study	HCWs aged 21-70 in GIAVA vaccination registry (1,607 received 2 doses and 447 unvaccinated)	N/A	Pfizer	PCR test-positive infection	14-20 d: 61.9% (19.2-82)<br>21-27 d: 87.9% (51.7-97)	7+ d: 96% (82.2-99.1)
F.P. Bianchi[B] (2021) Italy (12/20-03/21)	Retrospective cohort study	HCWs aged 20-70 at Bari Policlinico University Hospital (5,351 vaccinated and 787 unvaccinated)	N/A	Pfizer	PCR test-positive infection<br><br>Symptomatic infection	14-34 d: 97.7% (95.4-99)<br><br>14-34 d: 99.2% (96.4-99.8)	14-41 d: 94.8% (87-97.8)<br><br>14-41 d: 97.2% (90.3-99.2)
K.J. Bruxvoort (2021) USA (03/21-07/21)	Test-negative case-control study	Individuals age 18+ in the Kaiser Permanente Southern California Healthcare system (2,442 received 1 dose, 13,378 received 2 doses, and 77,750 unvaccinated)	B.1.617.2, B.1.1.7, P.1, epsilon, B.1.526, B.1.621, N/A	Moderna	PCR test-positive infection<br><br>Hospitalization	14+ d: B.1.1.7= 90.1% (82.9-94.2)<br>14+ d: B.1.617.2= 77% (60.7-86.5)<br>14+ d: B.1.427/ B.1.429= 76.3% (48.1-89.1)<br>14+ d: P.1/ P.1.1/ P.1.2 = 74.2% (43.8-88.1)<br>14+ d: other= 84.3% (65.9-92.7)<br>14+ d: unidentified= 67.6% (57.1-75.6)	14+ d: B.1.1.7= 98.4% (96.9-99.1)<br>14+ d: B.1.617.2= 86.7% (84.3-88.7)<br>14+ d: B.1.427/ B.1.429= 97.6% (90.2-99.4)<br>14+ d: P.1/P.1.1/P.1.2= 95.5% (90.9-97.8)<br>14+ d: B.1.526/ B.1.526.1/ B.1.526.2= 95.7% (81.7-99)<br>14+ d: B.1.621/ B.1.621.1= 90.4% (73.9-96.5)<br>14+ d: other= 96.4% (91.2-98.5)<br>14+ d: unidentified= 79.9% (76.9-82.5)<br><br>14+ d: B.1.617.2= 97.5% (92.7-99.2)

							14+ d: 96.6% (89.5-98.9)
A.A. Butt (2021) USA (12/20-03/21)	Test-negative case-control Days post vaccination not indicated	Veterans in the Veterans Affairs healthcare system (Pfizer: 4,622 received 1 dose, 2,366 received 2 doses. Moderna: 5,072 received 1 dose, 1,733 received 2 doses and 81,095 unvaccinated)	N/A	Pfizer and Moderna	PCR test-positive infections	(Pfizer) 84% (82.7-85.1)<br>(Moderna) 85.7% (84.6-86.8)	(Pfizer) 7+ d: 96.2% (95.5-96.8)<br>(Moderna) 7+ d: 98.2% (97.5-98.6)
T. Cerqueira-Silva (2022) Brazil (02/20-11/21)	Test-negative, case-control study	Individuals aged 18+ with data records in multiple national health databases (9,283 vaccinated and 97,856 unvaccinated)	N/A, P.1	Pfizer	Symptomatic infection<br><br>Hospitalization, Death	14+ d: 45% (39.7-49.9)<br><br>14+ d: 61.8% (40.8-75.3)	14+ d: 64.8% (54.9-72.4)<br><br>14+ d: 89.7% (54.3-97.7)
M. Chadeau-Hyam (2022) England (06/21- 09/21)	Test-negative, case-control (cross-sectional survey)	Individuals 18-64 years old in the National Health Service. (19,470 vaccinated with Pfizer, 1,225 vaccinated with Moderna, and 3,990 unvaccinated)	N/A	Pfizer and Moderna	PCR test-positive infection<br><br>Symptomatic	Not reported	(Pfizer) 14+ d: 71.3% (56.6-81)<br>(Moderna) 14+ d: 75.1% (22.7-92)<br><br>(Pfizer) 14+ d: 76.6% (59-86.6)
H. Chemaitelly (2021) Qatar (12/20-05/21)	Test-negative, case-control study	Adult healthcare records in the nationwide health database. (256,037 received at least 1 dose and 181,304 received 2 doses, 87,207 unvaccinated control for first dose and 82,073 unvaccinated control for second dose)	B.1.1.7., B.1.351	Moderna	PCR test-positive infections<br><br>Severe, critical, fatal	14- 20 d: B.1.1.7= 81.6% (73.1-87.8)<br>14-20 d: B.1.351= 47.9% (39.5-55.2)<br>21-27 d: B.1.1.7= 94.4% (89.1-97.5)<br>21-27 d: B.1.351= 73.7% (67.6-78.8)<br><br>14+ d: 81.6% (71-88)<br>14-20 d: 70.3% (48.9-83.5)<br>21-27 d: 92.1% (78.4-97.9)	7-13 d: B.1.1.7= 99.2% (95.3-100)<br>7-13 d: B.1.351= 96.4% (94.3-97.9)<br>14+ d: B.1.1.7= 100% (91.8-100)<br>14+ d: B.1.351= 96.4% (91.9-98.7)<br><br>7-13 d: 100% (86.9-100)<br>14+ d: 95.7% (73.4-99.9)

E.T. Chin (2022) USA (12/20-03/21)	Retrospective cohort	Individuals 18+ years old in California State prisons with healthcare records (29,947 vaccinated and 30,760 unvaccinated)	N/A	Pfizer and Moderna	PCR test-positive infection	7-13 d: 44% (20-61)<br>14+ d: 74% (64-82)	14+ d: 97% (88-99)
H. Chung (2021) Canada (12/20-04/21)	Test-negative, case-control study	Individuals 16+ years old in Ontario with records in various local health databases. (16,378 received 1 dose, 4,894 received 2 doses, and 302,761 unvaccinated)	Original, B.1.1.7., B.1.351, P.1.	Pfizer and Moderna	PCR test-positive infection<br><br>Symptomatic infection<br><br>Hospitalization/ death.	Not reported<br><br>14+ d: 60% (57-64)<br><br>14+ d: 70% (60-77)	7+ d: 91% (89-93)<br><br>Not reported<br><br>7+ d: 98% (88-100)
B.A Cohn (2021) USA (02/21-10/21)	Prospective cohort study	Individuals ages 18+ with records in the Veterans Health Administration. (230,762 received Moderna, 231,724 received Pfizer, and 282,077 unvaccinated)	N/A, B.1.617.2	Pfizer and Moderna	PCR test-positive infection	Not reported	(Pfizer) 14+ d: non-B.1.617.2= 86.9% (86.5-87.3)<br>(Pfizer) 14+ d: B.1.617.2= 43.3% (41.9-44.6)<br>(Moderna) 14+ d: non-B.1.617.2= 89.2% (88.8-89.6)<br>(Moderna) 14+ d: B.1.617.2= 58% (56.9-59.1)
N. Dagan (2021)[A] Israel (12/20-02/21)	Prospective cohort study	Individuals aged 16+ within an Israeli Healthcare provider (Clait). (596,618 vaccinated with at least 1 dose, 572,753 received 2 doses, and 596,618 unvaccinated controls with matched demographics)	B.1.1.7, N/A	Pfizer	PCR test-positive infection<br><br>Symptomatic infection<br><br>Hospitalization<br><br>Severe disease<br><br>Death<br><br>Asymptomatic	14-20 d: 46% (40-51)<br>21-27 d: 60% (53-66)<br><br>14-20 d: 57% (50-63)<br>21-27 d: 66% (57-73)<br><br>14-20 d: 74% (56-86)<br>21-27 d: 78% (61-91)<br><br>14-20 d: 62% (39-80)<br>21-27 d: 80% (59-94)<br><br>14-20 d: 72% (19-100)<br>21-27 d: 84% (44-100)<br><br>14-20 d: 29% (17-39)<br>21-27 d: 52% (41-60)	7+ d: 92% (88-95)<br><br>7+ d: 94% (87-98)<br><br>7+ d: 87% (55-100)<br><br>7+ d: 92% (75-100)<br><br>Not reported<br><br>7+ d: 90% (83-94)

N. Dagan (2021)[B] Israel (12/20- 06/21)	Retrospective cohort study	Pregnant women aged 16+ within an Israeli healthcare provider (Clait).<br>(10,861 vaccinated and 10,861 unvaccinated)	B.1.1.7, N/A	Pfizer	PCR test-positive infection<br><br>Symptomatic infection<br><br>Hospitalization	14-20 d: 67% (40-84)<br>21-27 d: 71% (33-94)<br><br>14-20 d: 66% (32-86)<br>21-27 d: 76% (30-100)<br><br>Not reported	7-56 d: 96% (89-100)<br><br>7-56 d: 97% (91-100)<br><br>7-56 d: 89% (43-100)
A. del Cura-Bilbao (2022) Spain (01/21-05/21)	Prospective cohort	Individuals aged 16+ in Aragon within the local health service.<br>(242,142 vaccinated with Pfizer, 32,522 vaccinated with Moderna, and 592,102 unvaccinated)	N/A	Pfizer and Moderna	PCR test-positive infection	(Pfizer) 12+ d: 20.8% (11.6-29)<br>(Moderna) 14+ d: 52.8% (30.7-67.8)	(Pfizer) 7+ d: 70% (65.3-74.1)<br>(Moderna) 14+ d: 70.3% (52.2-81.5)
A.A. Eick-Cost (2022) USA (01/21-09/21)	Case-control study	USA military personnel with laboratory records in the military health system.<br>(37,302 received Moderna, 79,624 received Pfizer, and 315,114 unvaccinated)	B.1.617.2, N/A	Pfizer	PCR test-positive infection<br><br>Asymptomatic infection<br><br>Symptomatic infection<br><br>Hospitalization	Not reported	(Moderna) 14+ d: non-B.1.617.2= 93.5% (91.9-94.7)<br>(Moderna) 14+ d: B.1.617.2= 79.4% (78.3-80.4)<br>(Pfizer) 14+ d: non-B.1.617.2= 87.6% (86.2-88.9)<br>(Pfizer) 14+ d: B.1.617.2= 69.3% (68.2-70.3)<br><br>(Moderna) 14+ d: non-B.1.617.2= 94.7% (91.9-96.6)<br>(Moderna) 14+ d: B.1.617.2= 77% (75.1-78.8)<br>(Pfizer) 14+ d: non-B.1.617.2= 80.3% (76.5-83.5)<br>(Pfizer) 14+ d: B.1.617.2= 66% (64-67.8)<br><br>(Moderna) 14+ d: non-B.1.617.2= 93.1% (91.2-94.6)<br>(Moderna) 14+ d: B.1.617.2= 80.6% (79.4-81.8)<br>(Pfizer) 14+ d: non-B.1.617.2= 89.9% (88.4-91.2)<br>(Pfizer) 14+ d: B.1.617.2= 71% (69.7-72.1)<br><br>(Moderna) 14+ d: non-B.1.617.2= 89.6% (57.5-97.4)<br>(Moderna) 14+ d: B.1.617.2= 88.1% (75.7-94.2)

							(Pfizer) 14+ d: non-B.1.617.2= 88% (75.4-94.1)<br>(Pfizer) 14+ d: B.1.617.2= 88.4% (82.1-92.5)
H.M. El Sahly (2021) USA (07/20-10/20)	Phase 3, observer blinded, placebo-controlled clinical trial.	Individuals aged 18+ across the USA.<br>(14,287 received vaccine and 14,164 received placebo)	N/A	Moderna	PCR test-positive infection<br><br>Asymptomatic infection<br><br>Symptomatic infection<br><br>Severe infection<br><br>Death	Not reported<br><br>Not reported<br><br>14+ d: 93.3% (91.1-94.9)<br><br>Not reported<br><br>Not reported	14+ d: 82% (79.5-84.2)<br><br>14+ d: 63% (56.6-68.5)<br><br>14+ d: 93.2% (91-94.8)<br><br>14+ d: 98.2% (92.8-99.6)<br><br>14+ d: 100% (N/A)
M. Fabiani (2021) Italy (12/20-03/21)	Retrospective cohort study	HCWs<br>(148 received 1 dose, 5,183 received 2 doses, and 1,092 non-vaccinated)	N/A	Pfizer	PCR test-positive infection<br><br>Symptomatic infection	14-21 d: 84.1% (39.7-95.8)<br>21+ d: 85.4% (-35.3-98.4)<br><br>14-21 d: 83.3% (14.8-96.7)<br>15-28 d: 86% (171-95.7)	7+ d: 95.1% (62.4-99.4)<br><br>7+ d: 93.7% (50.8-99.2)
ME. Flacco (2021); Italy (01/21-05/21)	Retrospective cohort study. 38 d follow up after vaccination	Individual's aged 18+ Province of Pescara with data recorded in the National health system.<br>(47,654 received 1 dose Pfizer, 4,888 received 1 dose Moderna<br>30,817 received 2 dose Pfizer and 2,020 received 2 dose Moderna and 388,058 unvaccinated)	B.1.1.7, N/A	Pfizer and Moderna	PCR test-positive infection<br><br>Symptomatic infection<br><br>Death	(Pfizer) 14+ d: 55% (N/A)<br><br>Not reported<br><br>Not reported	(Pfizer) 14+ d: 98% (N/A)<br>(Moderna) 14+ d: 100% (N/A)<br><br>(Pfizer) 14+ d: 99% (N/A)<br>(Moderna) 14+ d: 100% (N/A)<br><br>(Pfizer) 14+ d: 98% (N/A)<br>(Moderna) 14+ d: 100% (N/A)
A. Florea (2022) USA (06/21-09/21)	Prospective cohort	Individuals aged 18+ in Southern California with data in the electronic health record system<br>(927,004 vaccinated and 927,004 unvaccinated)	B.1.617.2, N/A	Moderna	PCR test-positive infection<br><br>Hospitalization<br><br>Death	Not reported	14+ d: 82.8% (82.2-83.3)<br>14+ d: B.1.617.2= 86.5% (84.8-88)<br><br>14+ d: 96.1% (95.5-96.6)<br><br>14+ d: 97.2% (94.8-98.4)

R.W Frenck Jr. (2021): USA (10/20-03/21)	Randomized, placebo-controlled, observer-blinded.	12–15-year-old with or without previous infection (1,119 received at least 1 dose, 1,097 received 2 doses and 1,110 received placebo)	N/A	Pfizer	Symptomatic infection	11+ d: 75% (7.6-95.5)	7+ d: 100% (78.1-100)
C. Giansante (2021) Italy (12/20-04/21)	Randomized controlled trial	HCWs aged 18+ in the Bologna Health trust. (7,897 received 2 doses, 296 received 1 dose and 1,646 unvaccinated)	N/A	Pfizer and Moderna	PCR test-positive infection<br><br>Symptomatic infection	14+ d: 85.5% (75.9-91.3)<br><br>14+ d: 81.7% (62.7-91)	7+ d: 84.8% (73.2-91.4)<br><br>7+ d: 87.1% (69.3-94.6)
Y. Goldberg (2022) Israel (12/20-03/21)	Prospective cohort study	Individuals aged 16+ with health records in the Israeli Ministry of Health database. (288.4 million person-days of follow-up for vaccinated individuals and 288.5 million person-days of follow-up for unvaccinated persons)	N/A	Pfizer	PCR test-positive infection<br><br>Hospitalization<br><br>Severe infection<br><br>Death	15+ d: 65.9% (65.4-66.4)<br><br>15+ d: 74.9% (73.5-76.3)<br><br>15+ d: 72.1% (69.9-74.1)<br><br>15+ d: 69.1% (65.5-72.3)	14+ d: 94.5% (94.3-94.7)<br><br>14+ d: 95.8% (95.2-96.2)<br><br>14+ d: 96.3% (95.7-96.9)<br><br>14+ d: 96% (94.9-96.9)
S. Goldin (2022) Israel (12/20-05/21)	Prospective cohort study	Long-term care residents aged 65+ from 454 different centres with data reported to the Ministry of Health. (39,482 vaccinated and 4,114 unvaccinated)	N/A	Pfizer	PCR test-positive infection<br><br>Death	10 d: 61.8% (58.2-65.1)<br><br>10 d: 72.3% (66.9-76.8)	Not reported
E.J. Haas (2021) Israel (01/21-04/21)	Prospective cohort	National surveillance of Individuals aged 16+ with data recorded to the Ministry of Health. (**4,714,932 received 2 doses and 1,823,979 unvaccinated)**	B.1.1.7, N/A	Pfizer	PCR test-positive infection<br><br>Symptomatic infection<br><br>Asymptomatic<br><br>Hospitalization	14-21 d: 57.7% (54.9-60.3)<br><br>14-21 d: 62.5% (59.3-65.4)<br><br>14-21 d: 52% (48.9-55)<br><br>14-21 d: 75.7% (72-79)	7+ d: 95.3% (94.9-95.7)<br>14+ d: 96.5% (96.3-96.8)<br><br>7+ d: 97% (96.7-97.2)<br>14+ d: 97.7% (97.5-97.9)<br><br>7+ d: 91.5% (90.7-92.2)<br>14+ d: 93.8% (93.3-94.2)<br><br>7+ d: 97.2% (96.8-97.5)

					Severe/critical infection<br><br>Death	14-21 d: 75.6% (71.9-78.9)<br><br>14-21 d: 77% (69.7-82.6)	14+ d: 98% (97.7-98.3)<br><br>7+ d: 97.5% (97.1-97.8)<br>14+ d: 98.4% (98.1-98.6)<br><br>7+ d: 96.7% (96-97.3)<br>14+ d: 98.1% (97.6-98.5)
V.J. Hall[A] (2021) England (12/20-02/21)	Prospective, cohort study	Hospital staff age 18+ in England with data reported via questionnaires and in the national immunization system. (17,952 received 1 dose and 1,561 received 2 doses 2,566 unvaccinated.)	B.1.1.7.	Pfizer	PCR test-positive infections.	21+ d: 70% (55-85)	7+ d: 85% (74-96)
V. Hall[B] (2022) UK (12/20-09/21)	Prospective cohort	HCWs aged 18+. (28,078 vaccinated with a long interval between doses and 891 unvaccinated)	N/A	Pfizer	PCR test-positive infection	21-27 d: 59% (42-71)<br><br>Reinfection within a year of previous infection<br>21-27 d: 92% (86-95)<br>Over a year from previous infection<br>21-17 d: 94% (62-99)	14-73 d: 85% (72-92)<br><br><br>14-73 d: 84% (67-92)<br><br>14-73 d: 94% (75-99)<br>74-133 days: 97% (93-98)
M. Husin (2022) Malaysia (09/21-12/21)	Test-negative, case-control study	Individuals 12-17 years old across Malaysia with data collected via Ministry of education registry (28,703 vaccinated and 65,292 unvaccinated)	N/A	Pfizer	PCR test-positive infection	Not reported	14+ d: 63.4% (64.4-66.9)
C Hyams (2021) UK (12/21-02/21)	Test-negative case-control study	Adults 80+ admitted to hospitals with records in the National Health Service. (108 vaccinated and 296 unvaccinated)	N/A	Pfizer	Hospitalization	14+ d: 71.4% (43.1-86.2)	Not reported
G.N. Ioannou (2022) USA (12/20- 06/21)	Case-control design	Individuals 18+ in Veterans Affairs healthcare system (2,099,871 received at least 1 dose and	N/A	Pfizer and Moderna	PCR test-positive infection	(Pfizer) 14-20 d: 31% (26-35)<br>(Pfizer) 21-27 d: 46% (41-50)<br>(Moderna) 14-27 d: 31% (26-35)<br>(Moderna) 28-35 d: 46% (41-50)	7+ d: 65% (63-68) reported in March<br>7+ d: 69% (67-70) reported in June

		1,635,948 unvaccinated)			Death	(Pfizer) 14-20 d: 55% (42-64)<br>(Pfizer) 21-27 d: 55% (39-67)<br>(Moderna) 14-27 d: 55% (42-64)<br>(Moderna) 28-35 d: 55% (39-67)	7+ d: 89% (84-92) reported in March 2021<br>7+ d: 86% (82-89) reported in June 2021
Y. June Choe (2022) South Korea (07/21-08/21)	Retrospective cohort study	Individuals 16-18 years old in high school with data in the Ministry of education and immunization registry. (444,313 received 1 dose, 442,025 received 2 doses, and 863,341 unvaccinated)	N/A	Pfizer	PCR test-positive infection	14+ d: 91.1% (89.6-92.5)	14+ d: 99.1% (98.5-99.5)
A.S Lauring (2022) USA (03/21-01/22)	Prospective, case-control cohort study	Individuals aged 18+ admitted to 21 hospitals across the USA with electronic medical records. (3,440 vaccinated and 2,054 unvaccinated)	B.1.1.7, B.1.617.2, B.1.1.529	Pfizer and Moderna	Hospitalization	Not reported	14+ d: B.1.1.7= 85% (82-88)<br>14+ d: B.1.617.2= 85% (83-87)<br>14+ d: B.1.1.529= 65% (51-75)
J. Lopez Bernal (2021)[A] England (12/20-02/21)	Test-negative case-control study	General Population aged 70+ in National Immunization System. (62,484 vaccinated and 18,061 unvaccinated)	B**.1.1.7, N/A**	Pfizer	Symptomatic infection	10-13 d: 70% (59-78)<br>28-34 d: 61% (51-69)	14+d: 89% (85-93)
Lopez Bernal (2021)[B] England (10/20-05/21)	Test-negative case-control design.	Health records of Individuals 16+ across the UK with records in the National Immunization system. (9,228 received 1 dose, 15,920 received 2 doses and 107,727 Unvaccinated)	B.1.617.2, B.1.1.7.	Pfizer	Symptomatic infection	21+ d: B.1.1.7= 47.5% (41.6-52.8)<br>21+ d: B.1.617.2= 35.6% (22.7-46.4)	14+ d: B.1.1.7= 93.7% (91.6-95.3)<br>14+ d: B.1.617.2= 88% (85.3-90.1)
H. Maeda (2022) Japan (07/21-09/21)	Test-negative, case-control study	Individuals aged 16+ admitted into hospitals across Japan (676 received Pfizer, 140 received Moderna, and 813 unvaccinated)	N/A	Pfizer and Moderna	Symptomatic infection	14+ d: ages 16-64= 54.3% (8.4-77.2)<br>(Moderna) 14+ d: ages 16-64= 80.8% (28.5-94.9)<br>(Pfizer) 14+ d: ages 16-64= 67.5% (-4.6-89.9)	14+ d: ages 16-64= 88.7% (78.8-93.9)<br>(Moderna) 14+ d: ages 16-64= 96.6% (72.8-99.6)<br>(Pfizer) 14+ d: ages 16-64= 86.7% (73.5-93.3)<br>14+ d: ages 65+= 90.3% (73.6-96.4)<br>(Pfizer) 14+ d: ages 65+= 85.8% (59.4-95)

C. Mallow (2022) USA (01/21-08/21)	Retrospective, test-negative study	Individuals aged 18+ admitted to University of Miami Hospital Emergency Department with electronic health records.<br>(3.242 vaccinated and 9,961 unvaccinated)	N/A	Pfizer and Moderna	Emergency department admission	Not reported	14+ d: 73.8% (66.2-79.7)<br>(Pfizer) 14+ d: 73.9% (66.3-79.8)<br>(Moderna) 14+ d: 78% (68.1-84.9)
T.F.D. Mason (2021) England (12/20-02/21)	Prospective cohort study	Health records of individuals 80-83 years old with records in the National health service (131,236 vaccinated and 131,236 unvaccinated)	N/A	Pfizer	PCR test-positive infection<br><br>Hospitalization<br><br>Emergency hospital attendance	21-27 d: 55.2% (40.8-66.8)<br>35-41 d: 70.1% (55.1-80.1)<br><br>21-27 d: 50.1% (19.9-69.5)<br>35-41 d: 75.6% (52.8-87.6)<br><br>21-27 d: 57.8% (30.8-74.5)<br>35-41 d: 78.9% (60-89.9)	Not reported
P.M. McKeigue (2022) Scotland (12/20-09/21)	Test-negative, case-control study	Individuals across Scotland with records in the public health electronic database (36,363 vaccinated and 185,826 unvaccinated)	N/A	Pfizer and Moderna	Severe infection<br><br>Hospitalization	Not reported	14+ d: 92% (88-95)<br><br>14+ d: 90% (88-92)
H.Q McLean (2022) USA (11/20-12/21)	Prospective, cohort	Individuals 12+ years old in Wisconsin with records in the Marshfield clinic health system health record.<br>(329 received Moderna, 608 received Pfizer, and 329 unvaccinated)	B.1.617.2, N/A	Pfizer and Moderna	PCR test-positive infection<br><br>Symptomatic infection	Not reported	14+ d: 56% (31-71)<br>(Moderna) 14+ d: 65% (37-81)<br>(Pfizer) 14+ d: 50% (21-69)<br>14+ d: B.1.617.2= 54% (27-71)<br>(Moderna) 14+ d: B.1.617.2= 59% (24-78)<br>(Pfizer) 14+ d: B.1.617.2= 52% (20-71)<br><br>14+ d: 58% (35-73)<br>(Moderna) 14+ d: 65% (38-81)<br>(Pfizer) 14+ d: 54% (26-71)
C. Menni (2021) UK (12/20-03/21)	Prospective, cohort study	Individuals aged 16+ reporting data via the study app.<br>(67,293 vaccinated and unvaccinated 464,356)	N/A	Pfizer	PCR test-positive infection	21-44 d: 69% (66-72)	Not reported
K. Muhsen (2021) Israel (01/21-04/21)	Prospective, cohort study	HCWs aged 16-65.<br>(6,960 vaccinated and 2,202 unvaccinated)	N/A	Pfizer	PCR test-positive infection	Not reported	14+ d: 89% (83-93)

P. Nordstrom (2022) Sweden (12/20-10/21)	Retrospective cohort study	Individuals across Sweden with data in the Swedish nationwide registers. (1,274,214 vaccinated with Pfizer, 153,760 vaccinated with Moderna, and 713,987 unvaccinated)	N/A	Pfizer and Moderna	PCR test-positive infection	Not reported	(Pfizer) 14+ d: 85% (84-85)<br>(Moderna) 14+ d: 89% (88-90)
C.R. Oliveira (2022) USA (06/21-08/21)	Case-control, study	Individuals 12-18 years old in Connecticut with available in local health system records. (134 vaccinated and 408 unvaccinated)	B.1.617.2, N/A	Pfizer	PCR test-positive infection<br><br>Asymptomatic infection<br><br>Symptomatic infection	14+ d: 74% (18-92)<br><br>Not reported<br><br>Not reported	14+ d: 90% (79-95)<br><br>14+ d: 85% (57-95)<br><br>14+ d: 93% (81-97)
S.M. Olson (2022) USA (07/21-10/21)	Test-negative, case-control study	Individuals 12-18 years old admitted to 31 hospitals across 23 states with available health records (299 vaccinated and 868 unvaccinated)	N/A	Pfizer	Hospitalization<br><br>ICU admission<br><br>Life support	Not reported	14+ d: 95% (91-97)<br><br>14+ d: 98% (94-100)<br><br>14+ d: 99% (93-100)
J. Pardo-Seco (2022) Spain (12/20- 03/21)	Test-negative, case-control study	Individuals 18+ years old in Northwest Spain with records in the Galician public health system. (169,104 vaccinated and 766,410 unvaccinated)	N/A	Pfizer	PCR test-positive infection<br><br>Hospitalization<br><br>ICU admission<br><br>Death	7-13 d: 64.3% (61.2-67.2)<br>14-20 d: 67.7% (64.6-70.6)<br><br>14+ d: 62% (54.2-68.2)<br><br>14+ d: 88% (74.6-95.8)<br><br>14+ d: 38% (15.9-55.4)	7-13 d: 75.4% (70.1-80.1)<br>14+ d: 90.8% (88.6-92.7)
C. Paris (2021) France (01/21-05/21)	Prospective cohort surveillance study	HCWs (239 received 1 dose and 472 received 2 doses of Pfizer. 201 received 1 dose and 423 received 2 doses of Moderna, and 1,380 unvaccinated.)	Origin, B.1.1.7, N/A	Pfizer and Moderna	PCR test-positive infection	(Moderna) 14+ d: 38.2% (6.3-59.2)<br>(Pfizer) 14+ d: 49.2% (19.1-68.1)<br>(Moderna) 14+ d: Origin= 57% (22-76)<br>(Pfizer) 14+ d: Origin= 49% (12-71)<br>(Pfizer) 14+ d: B.1.1.7= 55% (28-118)<br>(Moderna) 14+ d: B.1.1.7= 5% (0-152)	(Moderna) 14+ d: 100% (N/A)<br>(Pfizer) 14+ d: 94.6% (63-99.3)<br>(Pfizer) 14+ d: Origin= 94.9% (12-71)<br>(Pfizer) 14+ d: B.1.1.7= 100% (N/A)

D.Pascucci (2021) Italy (12/20-03/21)	Retrospective cohort	HCWs aged 20-76 from Fondazione Policlinico Universitario Agostino Gemelli IRCCS.<br>(5,152 vaccinated and 1,418 unvaccinated)	N/A	Pfizer	PCR test-positive infection	Not reported	14+ d: 91.5% (84.7-95.3)
C. Pawlowski (2021) USA (12/20- 04/21)	Retrospective cohort study	Individuals aged 18+ in Mayo Clinic health system<br>(51,795 received at least 1 dose of Pfizer, 16,471 received at least 1 dose of Moderna, and 68,266 unvaccinated)	N/A	Pfizer and Moderna	PCR test-positive infection<br><br><br><br>Hospitalization<br><br><br><br><br>ICU admission	(Pfizer) 7+ d: 45.5% (37.1-52.9)<br>(Pfizer) 14+ d: 61% (50.8-69.2)<br>(Moderna) 7+ d: 51.7% (37.3-63)<br>(Moderna) 14+ d: 66.6% (51.9-77.3)	(Pfizer) 7+ d: 86.1% (82.4-89.1)<br>(Pfizer) 14+ d: 88% (84.2-91)<br>(Moderna) 7+ d: 93.3% (85.7-97.4)<br>(Moderna) 14+ d: 92.3% (82.4-97.3)<br><br>(Pfizer) 7+ d: 88.8% (75.5-95.7)<br>(Pfizer) 14+ d: 88.3% (72.6-95.9)<br>(Moderna) 7+ d: 86% (71.6-93.9)<br>(Moderna) 14+ d: 90.6% (76.5-97.1)<br><br>(Moderna) 7+ d: 100% (43.3-100)<br>(Pfizer) 7+ d: 100% (51.4-100)
M. Perry (2022) Wales (12/20-07/21)	Retrospective cohort	Individuals aged 50+ with records in multiple national health databases.<br>(331,064 vaccinated and 93,439 unvaccinated)	B.1.1.7, B.1.617.2, N/A	Pfizer	PCR test-positive infection<br><br>Hospitalization	Not reported	7+ d: 50.1% (44-55.5)<br><br><br>7+ d: 88.2% (80.6-92.8)
T. Pilishvili (2021) USA (12/20- 05/21)	Test-negative, case-control study	HCWs aged 18+ from 33 sites across the USA.<br>(2,550 received Pfizer, 640 received Moderna, and 1,702 unvaccinated)	N/A	Pfizer and Moderna	PCR test-positive infection	10-13 d: 36.8% (14.8-53.1)<br>14+ d: 79.7% (74.1-84.1)<br>(Pfizer) 14+ d: 77.6% (70.9-82.7)<br>(Moderna) 14+ d: 88.9% (78.7-94.2)	7+ d: 90.4% (87-92.9)<br>(Pfizer) 7+ d: 88.8% (84.6-91.8)<br>(Moderna) 7+ d: 96.3% (91.3-98.4)
F.P. Polack (2021) USA, Argentina, Brazil, South Africa, Germany, Turkey (06/20-11/21)	Placebo-controlled, observer-blinded efficacy trial.	Individuals 16 years and older.<br>(21,720 vaccinated and 21,728 placebo)	N/A	Pfizer	Symptomatic Infection<br><br>Severe infection	**12+ d: 52.4% (29.5-68.4)**<br><br><br>**100% (-51.5-100)**	7+ d: 94.8 (89.8-97.6)<br><br><br>7+ d: 75% (-152.6-99.5)
E. Poukka (2022) Finland (12/20-10/21)	Prospective register-based cohort study	HCWs ages 16-69 years old	B.1.617.2, non-B.1.617.2	Pfizer and Moderna	PCR test-positive infection	**21-41 d: 48% (39-55)**<br>**21-41 d: non-B.1.617.2= 40% (25-52)**<br>**21-41 d: B.1.617.2= 56% (46-64)**	14-90 d: 82% (79-85)<br>14-90 d: **non-B.1.617.2= 77**% (71-82)<br>14-90 d: **B.1.617.2= 85**% (81-88)

		(338,020 vaccinated= 22,607 one dose, 315,413 two doses and 43,449 unvaccinated.)	(pre-B.1.617.2)		Hospitalization	(Pfizer) **21-41 d: 47% (38-55)**<br>**(Moderna) 21-41 d: 72% (47-85)**<br><br>**21-41 d: 95% (67-99)**<br>**21-41 d: non-B.1.617.2= 89% (15-98)**<br>**21-41 d: B.1.617.2= 100% (N/A)**<br>(Pfizer) **21-41 d: 95% (63-99)**<br>**(Moderna) 21-41 d: 100% (N/A)**	(Pfizer) 14-90 d: 83% (80-85)<br>**(Moderna)** 14-90 d: 84% (68-92)<br><br>14-90 d: 99% (97-100)<br>14-90 d: **non-B.1.617.2= 95**% (64-99)<br>14-90 d: **B.1.617.2= 100**% (97-100)<br>(Pfizer) 14-90 d: 99% (97-100)<br>**(Moderna)** 14-90 d: 100% (N/A)
K.B. Pouwels (2021) UK (12/20-08/21)	Prospective cohort study, self-reported symptoms via household survey	Individuals aged 18+ (for B.1.1.7 results only) and aged 18-64 (for B.1.617.2 results) with records in the National immunisation system. B.1.1.7(21,943 received 1 dose, 10,921 received 2 doses, 265,334 unvaccinated) B.1.617.2 (18,799 received 1 dose Pfizer, 95,350 received 2 doses Pfizer, 2,761 received 1 dose Moderna and 19,412 unvaccinated)	B.1.1.7, B.1.617.2	Pfizer	PCR test-positive infection<br><br>Symptomatic infection<br><br>Asymptomatic infection	**(Pfizer) 21+ d: B.1.1.7= 59% (52-65)**<br>**(Pfizer) 21+ d: B.1.617.2= 58% (51-63)**<br>**(Pfizer) 21+ d: B.1.617.2= prior infection= 91% (84-95)**<br>**(Pfizer) 21+ d: B.1.617.2= no prior= 57% (50-63)**<br>**(Moderna) 21+ d: B.1.617.2= 75% (64-83)**<br>**(Moderna) 21+ d: B.1.617.2= no prior= 76% (64-83)**<br>**(Moderna) 21+ d: B.1.617.2= prior= 86% (44-97)**<br><br>**(Pfizer) 21+ d: B.1.1.7= 73% (68-76)**<br>**(Pfizer) 21+ d: B.1.617.2= 59% (52-64)**<br>**(Pfizer) 21+ d: B.1.617.2= prior infection= 94% (87-98)**<br>**(Pfizer) 21+ d: B.1.617.2= no prior= 58% (50-65)**<br>**(Moderna) 21+ d: B.1.617.2= 77% (66-84)**<br>**(Moderna) 21+ d: B.1.617.2= no prior= 76% (62-85)**<br>**(Moderna) 21+ d: B.1.617.2= prior= 100% (100-100)**<br><br>**(Pfizer) 21+ d: B.1.617.2= 55% (48-61)**<br>**(Moderna) 21+ d: B.1.617.2= 70% (57-79)**	**(Pfizer)** 14+ d: B.1.1.7= 78% (68-84)<br>**(Pfizer)** 14+ d: B.1.617.2= 82% (79-85)<br>**(Pfizer)** 14+ d: B.1.617.2= prior infection=93% (87-96)<br>**(Pfizer)** 14+ d: B.1.617.2= no prior =85% (79-90)<br><br>**(Pfizer)** 14+ d: B.1.1.7= 97% (96-98)<br>**(Pfizer)** 14+ d: B.1.617.2= 86% (83-88)<br>**(Pfizer)** 14+ d: B.1.617.2= prior infection=99% (96-100)<br>**(Pfizer)** 14+ d: B.1.617.2= no prior =93% (89-97)<br><br>**(Pfizer)** 14+ d: B.1.617.2= 74% (69-78)
A.M. Price (2022) USA (07/21-02/22)	Test-negative, case-control study	Individuals 5-18 years old in hospitalized across USA with electronic medical records. (734 vaccinated and 2,078 unvaccinated)	B.1.617.2, B.1.1.529	Pfizer	Hospitalization<br><br>Critical Infection	Not reported	14+ d: ages 12-15= 83% (77-88)<br>14+ d: ages 16-18= 82% (74-88)<br>14+ d: ages 5-11, B.1.1.529= 68% (42-82)<br><br>14+ d: B.1.617.2= 96% (90-98)<br>14+ d: B.1.1.529= 79% (51-91)

E. Pritchard (2021) UK (12/20-05/21)	Prospective cohort Infection Survey.	Individuals 16+ with data in the Office for National Statistics. (81,171 received 1 dose, 57,646 received 2 doses, and 329,419 unvaccinated)	B.1.1.7., N/A	Pfizer	PCR test-positive infections	21+ d: 66% (60-71)	7-30 d: 80% (59-90)
M. Risk (2022) USA (01/21-10/21)	Prospective cohort study	Individuals 18+ years old with records in the Michigan Medicine healthcare system. (73,666 received Pfizer, 34,474 received Moderna, 45,119 unvaccinated)	B.1.617.2, N/A	Pfizer and Moderna	PCR test-positive infection<br><br><br><br>Hospitalization	Not reported	(Pfizer) 14+ d: 67% (63-70)<br>**(Moderna)** 14+ d: 79% (75-82)<br>(Pfizer) 14+ d: non-B.1.617.2= 87% (N/A)<br>(Pfizer) 14+ d: B.1.617.2= 52% (N/A)<br>**(Moderna)** 14+ d: non-B.1.617.2= 92% (N/A)<br>**(Moderna)** 14+ d: B.1.617.2= 70% (N/A)<br><br>(Pfizer) 14+ d: 87% (81-92)<br>**(Moderna)** 14+ d: 93% (86-97)<br>(Pfizer) 14+ d: non-B.1.617.2= 95% (N/A)<br>(Pfizer) 14+ d: B.1.617.2= 82% (N/A)
E.S. Rosenberg (2022) USA (05/21-08/21)	Retrospective cohort study.	Individuals aged 18+ in New York State vaccinated in April 2021 with records in multiple local healthcare databases in May and August. (1,558,943 received Pfizer, 1,081,998 received Moderna, and 3,052,683 unvaccinated)	N/A, B.1.617.2	Pfizer and Moderna	PCR test-positive infection	Not reported	(Pfizer) 14+ d: May, ages 18-49= 95.5% (94.4-96.6)<br>(Pfizer) 14+ d: August, B.1.617.2, ages 18-49= 68.6% (66.5-70.7)<br>(Pfizer) 14+ d: May, ages 50-64= 97% (96-97.9)<br>(Pfizer) 14+ d: August, B.1.617.2, ages 50-64= 76.1% (74-78.2)<br>(Pfizer) 14+ d: May, ages 65+= 94.9% (93.4-96.4)<br>(Pfizer) 14+ d: August, B.1.617.2, ages 65+= 77.5% (74.9-80.1)<br>(Moderna) 14+ d: May, ages 18-49= 97.5% (96.4-98.6)<br>(Moderna) 14+ d: August, B.1.617.2, ages 18-49= 82% (79.9-84.1)<br>(Moderna) 14+ d: May, ages 50-64= 98% (97.1-99)<br>(Moderna) 14+ d: August, B.1.617.2, ages 50-64= 86.8% (84.9-88.6)<br>(Moderna) 14+ d: May, ages 65+= 95.7% (94.4-97.1)<br>(Moderna) 14+ d: August, B.1.617.2, ages 65+= 84.8% (82.7-86.8)

					Hospitalization		(Pfizer) 14+ d: May, ages 18-49= 94.8% (91.7-96.9)<br>(Pfizer) 14+ d: August, B.1.617.2, ages 18-49= 96.4% (94.5-97.8)<br>(Pfizer) 14+ d: May, ages 50-64= 96.8% (95.3-97.9)<br>(Pfizer) 14+ d: August, B.1.617.2, ages 50-64= 94.8% (93.5-95.9)<br>(Pfizer) 14+ d: May, ages 65+= 95% (93.8-96.1)<br>(Pfizer) 14+ d: August, B.1.617.2, ages 65+= 89.8% (88.3-91.2)<br>(Moderna) 14+ d: May, ages 18-49= 95.7% (91.5-98.1)<br>(Moderna) 14+ d: August, B.1.617.2, ages 18-49= 97.1% (94.6-98.7)<br>(Moderna) 14+ d: May, ages 50-64= 95.9% (93.6-97.5)<br>(Moderna) 14+ d: August, B.1.617.2, ages 50-64= 96.3% (94.8-97.4)<br>(Moderna) 14+ d: May, ages 65+= 96.8% (95.8-97.6)<br>(Moderna) 14+ d: August, B.1.617.2, ages 65+= 93.9% (892.8-95)
Y. Saciuk (2022) Israel (01/21-04/21)	Retrospective cohort study	Individuals 16 years and older with records in an Israeli healthcare service. (Maccabi) (1,347,976 vaccinated and 1,075,626 unvaccinated)	N/A	Pfizer	PCR test-positive infection<br><br>Hospitalization<br><br>Death	Not reported	8+ d: 93% (92.6-93.4)<br><br>8+ d: 93.4% (91.9-94.7)<br><br>8+ d: 91.1% (87-94)
D.M. Skowronski (2021) Canada (04/21-05/21)	Test-negative case-control study	Community individuals 70+ years old in British Columbia with records in the local Public Health database (10,569 received Pfizer, 1,882 received Moderna, and 4,542 unvaccinated)	B.1.1.7, P.1, N/A	Pfizer and Moderna	PCR test-positive infection	21+ d: 65% (58-71)<br>21+ d: B.1.1.7= 67% (57-75)<br>21+ d: P.1= 61% (45-72)<br>(Pfizer) 21+ d: 64% (57-71)<br>(Moderna) 21+ d: 71% (56-81)	Not reported
J.L. Suah[A] (2021)	Retrospective cohort study	Individuals aged 18+ in Malaysia with records in the Ministry of health	N/A	Pfizer	ICU admission<br><br>Death	14+ d: 34.3% (30.2-38.1)<br><br>14+ d: 48.1% (44.5-51.4)	14+ d: 90.3% (88.8-91.6)<br><br>14+ d: 92.7% (91.7-93.6)

Malaysia (04/21-09/21)		(92,677 received 1 dose, 83,923 received 2 doses, and 749,524 unvaccinated)					
J.L. Suah[B] (2022) Malaysia (09/21)	Retrospective cohort study	Individuals aged 15+ in Malaysia with records in the Ministry of health (4,357,798 vaccinated and 81,987 unvaccinated)	N/A, B.1.617.2	Pfizer	PCR test-positive infection<br><br>ICU Admission<br><br>Death	Not reported	14 d-2 mo: 90.8% (89.4-92.1)<br><br>14 d-2 mo: 86% (82.8-88.6)<br><br>14 d-2 mo: 91.5% (89.8-92.9)
P. Tang (2021) Qatar (03/21-09/21)	Test-negative, case-control study	Individuals within the Hamad Medical Corporation healthcare provider. (34,462 controls and 8,498 cases for the first dose. 45,618 controls and 10,943 cases for the second dose)	B.1.617.2 and B.1.1.7	Pfizer and Moderna	PCR test-positive infection<br><br>Symptomatic infection<br><br>Asymptomatic infection<br><br>Severe/critical/ fatal	(Pfizer) 14+ d: B.1.617.2= 45.3% (22-61.6)<br>(Moderna) 14+ d: B.1.617.2= 73.7% (58.1-83.5)<br>14+ d: B.1.617.2= 58% (44.4-68.2)<br>(Pfizer) 14+ d: B.1.1.7 = 18.9% (-1.8-35.4)<br>(Moderna) 14+ d: B.1.1.7 = 66.3% (55.8-74.2)<br>14+ d: B.1.1.7= 44.9% (34.7-53.5)<br><br>(Pfizer) 14+ d: B.1.617.2= 56.2% (30.6-72.4)<br>(Moderna) 14+ d: B.1.617.2= 82.5% (65.2-91.2)<br>14+ d: B.1.617.2= 67.3% (52.4-77.6)<br><br>(Pfizer) 14+ d: B.1.617.2= 6.7% (-56.2-81.8)<br>(Moderna) 14+ d: B.1.617.2= 61.8% (-9.6-86.7)<br>14+ d: B.1.617.2= 47% (-13.8-75.3)<br><br>(Pfizer) 14+ d: B.1.1.7 = 74.8% (-7.6-94.1)<br>(Moderna) 14+ d: B.1.1.7 = 72.5% (7.7-91.8)<br>14+ d: B.1.1.7= 67.7% (28.6-85.4)	(Pfizer) 14+ d: B.1.617.2= 51.9% (47-56.4)<br>(Moderna) 14+ d: B.1.617.2= 73.1% (67.5-77.8)<br>14+ d: B.1.617.2= 55.5% (51.2-59.4)<br>(Pfizer) 14+ d: B.1.1.7 = 74.3% (70.3-77.7)<br>(Moderna) 14+ d: B.1.1.7 = 80.8% (69-88.2)<br>14+ d: B.1.1.7= 76.4% (72.9-79.4)<br><br>(Pfizer) 14+ d: B.1.617.2= 44.4% (37-50.9)<br>(Moderna) 14+ d: B.1.617.2= 73.9% (65.9-79.9)<br>14+ d: B.1.617.2= 49.2% (42.8-54.9)<br><br>(Pfizer) 14+ d: B.1.617.2= 46% (32.3-56.9)<br>(Moderna) 14+ d: B.1.617.2= 53.6% (33.4-67.6)<br>14+ d: B.1.617.2= 45.9% (33.3-56.1)<br><br>(Pfizer) 14+ d: B.1.617.2= 93.4% (85.4-97)<br>14+ d: B.1.617.2= 93.6% (85.9-97.1)<br>(Pfizer) 14+ d: B.1.1.7 = 92.7% (81.5-97.1)<br>(Moderna) 14+ d: B.1.1.7 = 100% (N/A)<br>14+ d: B.1.1.7= 93.4% (83.5-97.4)
S.Y. Tartof [A] (2021) USA (12/20-08/21)	Retrospective cohort study	Individuals 12+ year old members of healthcare organization Kaiser Permanente Southern California (103,479 received 1 dose, 1,043,289 received 2 doses, and 2,290,189 unvaccinated)	B.1.617.2, N/A	Pfizer	PCR test-positive infection<br><br>Hospitalization	14+ d: 58% (54-61)<br>14+ d: B.1.617.2= 75% (58-86)<br>14+ d: non-B.1.617.2= 74% (64-81)<br><br>14+ d: 54% (43-63)<br>14+ d: B.1.617.2= 79% (-54-97)<br>14+ d: non-B.1.617.2= 75% (21-92)	7+ d: 73% (72-74)<br>7+ d: B.1.617.2= 75% (71-78)<br>7+ d: non-B.1.617.2= 91% (88-92)<br><br>7+ d: 90% (89-92)<br>7+ d: B.1.617.2= 93% (84-96)<br>7+ d: non-B.1.617.2= 95% (90-98)

S.Y Tartof [B] (2022) USA (12/21/02/22)	Test-negative case-control	Individuals 18+ in the Kaiser Permanente Southern California healthcare system, admitted to hospitals across the USA (3,981 vaccinated and 5,165 unvaccinated)	B.1.617.2, B.1.1.529	Pfizer	Hospitalization<br><br>Emergency department admission	Not reported	7+ d: B.1.617.2= 76% (69-82)<br>7+ d: B.1.1.529= 62% (53-69)<br><br>7+ d: B.1.617.2= 61% (55-66)<br>7+ d: B.1.1.529= 47% (40-54)
M.W. Tenforde (2021) USA (03/21-05/21)	Test-negative, case-control study	Individuals ages 18+ admitted to 18 hospitals across the USA with health records. (408 vaccinated and 729 unvaccinated)	B.1.1.7, N/A	Pfizer and Moderna	Hospitalization	14+ d: 76.1% (64-84.2)	14+ d: 87.1% (80.7-91.3)<br>14+ d: B.1.1.7= 92.4% (83.6-96.5)<br>(Pfizer) 14+ d: 84.4% (74.9-90.4)<br>(Moderna) 14+ d: 90.1% (82.3-94.5)
S.J. Thomas (2021) USA, Argentina, Brazil, Germany, South Africa, Turkey (07/20-01/21)	Randomized, placebo-controlled, observed-blinded trial	Individuals 12+ years and older. (23,040 vaccinated and 23,037 placebo)	B.1.351, N/A	Pfizer	PCR test-positive infection<br><br>Severe disease	11- 20 d: 91.7% (79.6-97.4)<br><br>11-20d: 96.7% (80.3-99.9)	7d-6mo: 91.1% (88.8-93)<br><br>7d – 6mo: 95.7% (73.9-99.9)
M. G. Thompson (2021) USA (01/21- 06/21)	Test-negative design based on patients accessing health care for SARS-CoV-2 clinical signs	ICU/ ambulatory patients age 50+, in 187 hospitals, 167 emergency departments, 54 urgent care units with health records. (Hospital/urgent care: 5,532 received 1 dose, 14,874 received 2 does, and 20,406 unvaccinated. Emergency care: 3,645 received 1 dose, 6,065 received 2 doses, and 11,812 unvaccinated)	N/A	Pfizer and Moderna	Hospitalization<br><br>Infection leading to ICU<br><br>Severe infection leading to urgent care or emergency visits	**14+ d: 54% (47-61)**<br><br>**Not reported**<br><br>**14+ d: 68% (61-74)**	14+ d: 89% (87-91)<br><br>14+ d: 90% (86-90)<br><br>14+ d: 91% (89-93)
H.F. Tseng (2022) USA (12/21)	Test-negative, case-control study	Individuals aged 18+ across Southern California with records	B.1.617.2, B.1.1.529	Moderna	PCR test-positive infection	**14+ d: B.1.617.2= 56.7% (40.7-68.4)**<br>**14+ d: B.1.1.529= 20.4% (9.5-30)**	14+ d: B.1.617.2= 63.6% (59.9-66.9)<br>14+ d: B.1.1.529= 13.9% (10.5-17.1)

		in the Kaiser Permanente Southern California healthcare system.<br>(1,892 received 1 dose, 53,649 received 2 doses, and 43,024 unvaccinated)			Hospitalization	**14+ d: B.1.617.2= 71.2% (-68.7-97.4)**	**14+ d: B.1.617.2= 99% (93.3-99.9)**<br>**14+ d: B.1.1.529= 84.5% (23-96.9)**
E. Vasileiou, (2021) Scotland (12/20-02/21)	Prospective, cohort study.	Individuals aged 18+ with records in multiple national healthcare databases<br>(359,434 vaccinated aged 18-64,<br>315,620 vaccinated individuals aged 65-79,<br>36,785 vaccinated individuals aged 80+ and<br>734,031 unvaccinated)	N/A	Pfizer	Hospital admissions	Total population:<br>14-20 d: 69% (62-75)<br>21-27 d:78% (71-83)<br>28-34 d: 91% (85-94)	Not reported
Z. Voko (2022) Hungary (01/21-06/21)	Prospective cohort study	Individuals 16+ years old in Hungary with records in the National Public Health Centre (1,497,011 received Pfizer, 222,892 received Moderna, 12,855 unvaccinated per 100,000 person days),	N/A, B.1.1.7	Pfizer and Moderna	PCR test-positive infection<br><br><br><br><br><br><br>Death	Not reported	(Pfizer) 7+ d: 83.3% (82.6-83.9)<br>(Moderna) 7+ d: 88.7% (86.6-90.4)<br>(Pfizer) 14+ d: 84% (83.3-84.7)<br>(Moderna) 14+ d: 88.2% (85.8-90.3)<br>(Pfizer) 28+ d: 82.5% (81.7-83.3)<br>(Moderna) 28+ d: 87% (83.2-90)<br><br>(Pfizer) 7+ d: 90.6% (89.4-91.7)<br>(Moderna) 7+ d: 93.6% (90.5-95.7)<br>(Pfizer) 14+ d: 90.3% (88.9-91.5)<br>(Moderna) 14+ d: 93.8% (90.3-96.1)<br>(Pfizer) 28+ d: 91.2% (89.6-92.5)<br>(Moderna) 28+ d: 93.5% (88.5-96.3)
E.B. Walter (2022) USA, Spain, Finland, Poland (06/21)	Phase 2-3 randomized controlled trial	Individuals aged 5-11 (1,518 vaccinated and 750 placebo)	N/A	Pfizer	PCR test-positive infection	Not reported	7+ d: 90.7% (67.4-98.3)
T.N.A. Winkelman (2022) USA (08/21-10/21)	Test-negative, case-control study	Individuals 19+ across Multiple Minnesota health databases (151,315 vaccinated with Pfizer, 98,853	N/A	Pfizer and Moderna	PCR test-positive infection<br><br>Hospitalization	Not reported	(Pfizer) 14+ d: 53% (52-54)<br>(Moderna) 14+ d: 66% (65-67)<br><br>(Pfizer) 14+ d: 81% (79-82)<br>(Moderna) 14+ d: 81% (79-82)

		vaccinated with Moderna, and 328,835 unvaccinated)					
B.J. Wright (2022) USA (04/21-10/21)	Test-negative, case-control study	Individuals ages 18+ admitted to 42 hospitals across 6 States.<br>(10,489 received Moderna, 10,516 received Pfizer, and 25,033 unvaccinated)	N/A	Pfizer and Moderna	Hospitalization	Not reported	(Moderna) 50-100 d: 97.3% (96-98.2)<br>(Pfizer) 50-100 d: 94.9% (93.2-96.2)
A. Yassi (2021) Canada (03/20-05/21)	Prospective surveillance cohort study.	HCWs from Vancouver (7,328 received 2 doses, 14,790 received 1 dose, and 3,440 unvaccinated)	B.1.1.7, P.1, N/A	Pfizer	PCR test-positive infection	14+ d: 37.2% (16.6-52.7)	7+ d: 79.2% (64.6-87.8)
Y. Young-Xu (2021) USA (12/20-03/21)	Test-negative, case-control	Individuals aged 18+ registered in the Veterans Health Administration.<br>(1,363,180 vaccinated with at least 1 dose, and 5,284,553 unvaccinated)	N/A	Pfizer and Moderna	PCR test-positive infection<br><br>Symptomatic infection<br><br>Hospitalization<br><br>Death	14+ d: 64% (59-68)<br><br>14+ d: 52% (44-60)<br><br>14+ d: 48% (32-60)<br><br>14+ d: 63% (24-81)	14+ d: 95% (93-96)<br><br>14+ d: 93% (89-95)<br><br>14+ d: 91% (83-95)<br><br>14+ d: 100 (N/A)
G Zacay (2021) Israel (01/21- 02/21)	Retrospective cohort study	Individuals aged 16+ with records in Meuhedet’s Health Maintenance Organization<br>(1,445 received 1 dose, 2,941 received 2 doses, and 1,900 unvaccinated)	N/A	Pfizer	PCR test-positive infection	14+ d: 61% (49-71)	7+ d: 89% (82-94)

Supplementary Table 2. Population-level efficacy of vaccination with AstraZeneca (ChAdOx1 nCoV-19 (AZD1222)) vaccine against test positivity, clinical signs, hospitalization, or death due to SARS-CoV-2.

Study; Country	Study design	Study Population (n)	Variant of interest	Outcome(s) measured	Adjusted vaccine efficacy (95% CI)	
					**After dose 1**	**After dose 2**
W.Q. Alali (2021) Kuwait (12/20-06/21)	Retrospective cohort study	Healthcare workers (HCWs) ages 20+.<br>(1,636 vaccinated and 581 unvaccinated)	N/A	Symptomatic infection	28+ d: 75.4% (67.2-81.6)	Not reported
G. Amirthalingam (2021) England (10/20-06/21)	**Test-negative, case-control study**	Individuals aged 50-79 with records in the national immunisation system. the National Health service.<br>(111 received 1 dose, 232 received 2 doses, and 274,093 unvaccinated)	N/A	PCR test-positive infection	Age 65-79<br>14-27 d: 33% (27-39)<br>28+ d: 52% (46-56)	Age 50-64<br>14+ d: (45-64 d between dose)= 70% (66-74)<br>Age 65-79<br>14+ d: (45-64 d between dose)= 61% (47-70)
N. Andrews[A] (2022) England (1/21-01/22)	**Test-negative case-control**	Individuals 18+ years old with records in the National immunization system.<br>(452,622 vaccinated, and 244,716 unvaccinated)	B.1.617.2, B.1.1.529	Symptomatic infection	4+ wks: B.1.1.529= 17.7% (14.3-21)<br>4+ wks: B.1.617.2= 42.9% (39.8-45.9)	2-4 wks: B.1.1.529= 48.9% (39.2-57.1)<br>2-4 wks: B.1.617.2= 82.8% (74.5-88.4)
N. Andrews[B] (2022) England (12/20-10/21)	**Test-negative, case-control study**	Individuals ages 16+ across England with records in the National immunization system.<br>(2,452,457 vaccinated and 855,743 unvaccinated.)	B.1.1.7, B.1.617.2, N/A	Symptomatic infection<br><br><br><br><br>Hospitalization<br><br><br><br><br>Death	28+ d: B.1.617.2= 46.6% (45.8-47.5)<br>28+ d: B.1.1.7= 45.1% (43.4-46.7)<br><br><br><br>28+ d: B.1.617.2= 80.7% (78-83)<br>28+ d: B.1.1.7= 84% (80.2-87.1)<br><br><br><br>28+ d: B.1.617.2= 86.9% (77.5-92.4)<br>28+ d: B.1.1.7= 86.9% (77.5-92.4)	1 wk: B.1.617.2= 64.8% (63.8-65.8)<br>2-9 wk: B.1.617.2= 67.6% (67.3-67.9)<br>1 wk: B.1.1.7= 71.8% (66.2-76.5)<br>2-9 wk: B.1.1.7= 82.4% (79.6-84.7)<br><br>1 wk: B.1.617.2= 94% (91.3-95.8)<br>2-9 wk: B.1.617.2= 95.2% (94.7-95.7)<br>1 wk: B.1.1.7= 89.6% (67.4-96.7)<br>2-9 wk: B.1.1.7= 95.1% (86.7-98.2)<br><br>2-9 wk: B.1.617.2= 95% (93.1-96.4)
T. Cerqueira-Silva (2022) Brazil (02/20-11/21)	Test-negative, case-control study	Individuals aged 18+ with data records in multiple national health databases.<br>(26,579 vaccinated and 97,856 unvaccinated)	N/A, P.1	Symptomatic infection<br><br>Hospitalization	14+ d: 34.2% (30.1-38.1)<br><br>14+ d: 56.9% (45.2-66.1)	14+ d: 56% (51.4-60.2)<br><br>14+ d: 89.9% (83.5-93.8)
M. Chadeau-Hyam (2022)	Test-negative case-control	Individuals 18-64 years old in the National Health Service.	N/A	PCR test-positive infection<br><br>Symptomatic infection	Not reported	14+ d: 44.8% (22.5-60.7)<br><br>14+ d: 45.5% (15.5-64.9)

England (06/21-09/21)		(56,581 vaccinated and 3,990 unvaccinated)				
S.A.C. Clemens (2021) Brazil (06/20-02/21)	Phase 3 randomized controlled trial	Individuals aged 18+ (4,772 vaccinated and 4,661 placebo)	P.2, B.1.1.28, P.1, B.1.1.33, N/A	Symptomatic infection<br><br><br><br><br>Hospitalization<br><br>Severe infection		15+ d: 56.6% (28.2-73.8)<br>15+ d: B.1.1.33= 88.2% (5.4-98.5)<br>15+ d: B.1.1.28= 72.6% (46.4-86)<br>15+ d: P.2= 68.7% (54.9-78.3)<br>15+ d: P.1= 63.6% (-2.1-87)<br><br>15+ d: 95% (61-99)<br><br>15+ d: 100% (N/A)
A. del Cura-Bilbao (2022) Spain (01/21-05/21)	Prospective cohort	Individuals aged 16+ in Aragon within the local health service. (97,492 vaccinated and 592,102 unvaccinated)	N/A	PCR test-positive infection	21+ d: 40.3% (31.8-47.7)	Not reported
K.R.W. Emary (2021) UK (10/20-01/21)	Randomised, controlled trial.	HCWs aged 18+. (1,471 received 2 doses, 4,290 control)	B.1.1.7. (UK), N/A	PCR test-positive infection<br><br><br>Symptomatic infection<br><br><br>Asymptomatic infection	Not reported	14+ d: B.1.1.7= 61.7% (36.7-76.9)<br>14+ d: non-B.1.1.7= 77.3% (65.4-85)<br><br>14+ d: B.1.1.7. = 70.4% (43.6-84.5)<br>14+ d: non-B.1.1.7. = 81.5% (67.9-89.4)<br><br>14+ d: B.1.1.7= 28.9% (-77.1-71.4)<br>14+ d: non-B.1.1.7= 69.7% (33-86.3)
A.R. Falsey (2021) USA, Chile, Peru (08/20-01/21)	Randomised, placebo-controlled trial	Individuals 18+ years of age from 88 sites across USA, Chile, and Peru. (21,635 vaccinated and 10,816 unvaccinated)	N/A	PCR test-positive infection<br><br>Symptomatic infection<br><br>Severe/critical infection<br><br>Infection leading to emergency hospitalization<br><br>Hospitalization<br><br>ICU admission	Not reported	15+ d: 64.3% (56.1-71)<br><br>15+ d: 74% (65.3-80.5)<br><br>15+ d: 100% (71.6- N/A)<br><br>15+ d: 94.8% (59-99.3)<br><br><br>15+ d: 94.2% (53.3-99.3)<br><br>15+ d: 100% (-1781.6-N/A)
ME. Flacco (2021); Italy (01/21-05/21)	Retrospective cohort study	Individual's aged 18+ in the Province of Pescara with data recorded in the National health system. (175,687 unvaccinated and 16,997 vaccinated)	B.1.1.7, N/A	PCR test-positive infection<br><br>Symptomatic infection	Not reported	14+ d: 95% (N/A)<br><br><br>14+ d: 100% (N/A)

V.F.F.P.H. Hall[B] (2022) UK (12/20-09/21)	Prospective cohort	HCWs aged 18+.<br>(2,803 vaccinated and 891 unvaccinated)	N/A	PCR test-positive infection	21-27 d: 63% (-80-92)	14-73 d: 58% (23-77)
M.D.T. Hitchings (2021) Brazil (01/21-07/21)	Test-negative, case-control study	Individuals 60+ in Sao Paulo with records in multiple national health databases (11,277 vaccinated and 50,083 unvaccinated)	P.1, N/A	Symptomatic infection<br><br>Hospitalization<br><br>ICU admission<br><br>Death	14-27 d: 17.8% (8-26.5)<br>28+ d: 33.4% (26.4-39.7)<br><br>14-27 d: 33.6% (19.9-45)<br>28+ d: 55.1% (46.6-62.2)<br><br>14-27 d: 39.6% (15.4-56.8)<br>28+ d: 50.9% (33.6-63.8)<br><br>14-27 d: 37.5% (15.2-54)<br>28+ d: 61.8% (48.9-71.4)	14+ d: 77.9% (69.2-84.2)<br><br>14+ d: 87.6% (78.2-92.9)<br><br>14+ d: 89.9% (70.9-96.5)<br><br>14+ d: 93.6% (81.9-97.7)
S.V. Katikireddi (2022) Scotland (05/21-10/21)	Retrospective cohort, test-negative case-control	Individuals aged 18+ in Scotland with data in national health databases.<br>(58,375 received 1 dose, 1,972,454 received 2 doses and 503,455 unvaccinated)	B.1.617.2, N/A	Symptomatic infection<br><br>Hospitalization/ Death	2+ wk: 37.6% (34.6-40.5)<br><br>2+ wk: 49.3% (43.3-54.6)	2-3 wk: 67.9% (65.9-69.8)<br>4-5 wk: 67.3% (65.3-69.1)<br><br>2-3 wk: 83.7% (79.7-87)<br>4-5 wk: 86.6% (83.6-89)
J. Lopez Bernal (2021)[A] UK (12/20-02/21)	Case-control, test-negative study	General Population aged 70+ in National Immunization System.<br>(18,061 unvaccinated and 76,385 vaccinated)	B.1.1.7, N/A	Symptomatic infection	28-34 d: 60% (41-73)<br>35+ d: 73% (27-90)	Not reported
Lopez Bernal (2021)[B] England (10/20-05/21)	Test-negative case-control design.	Health records of Individuals 16+ across the UK with records in the national immunization system.<br>(45,961 received 1 dose, 8,556 received 2 doses, and 107,727 unvaccinated)	B.1.617.2, B.1.1.7.	Symptomatic infection	21+ d: B.1.1.7= 48.7% (45.2-51.9)<br>21+ d: B.1.617.2= 30% (24.3-35.3)	14+ d: B.1.1.7= 74.5% (68.4-79.4)<br>14+ d: B.1.617.2= 67% (61.3-71.8)
S.A. Madhi (2021) South Africa (05/20-11/20)	Multicentre, double-blind, randomized, controlled trial.	General Public 18–65-year-old. (944 vaccinated: 804 seronegative and 135 seropositive at baseline.<br>938 controls:<br>776 seronegative and 153 seropositive) Total cohort included seronegative and seropositive participants.	B.1.351, N/A	Mild-to-moderate<br><br>Moderate<br><br>PCR test-positive infection	14+ d: B.1.351= 33.5% (-13.4-61.7)<br>14+ d: B.1.351= in seronegative=37.5%<br>14+ d: non-B.1.351= 75.4% (8.7-95.5)<br>14+ d: non-B.1.351. seronegative= 72.8%<br><br>14+ d: B.1.351= 42.6% (-58-80.9)<br>14+ d: non-B.1.351= 75.4% (-148.5-99.5)<br>14+ d: non-B.1.351. seronegative= 76.2%<br><br>14+ d: non-B.1.351= 46.7% (-8.9-75.1)	14+ d: seronegative= 21.9%<br>14+ d: B.1.351= 10.6% (-66.4-52.2)<br><br>14+ d: B.1.351= 2.5% (-264.6-73.9)<br><br>14+ d: B.1.351= 19.2% (-36-52.4)

				Symptomatic infection	14+ d: non-B.1.351 in seronegative= 42.9% (95% C)<br>14+ d: B.1.351= 30% (-5.8-54)<br>14+ d: B.1.351= in seronegative= 33.4%<br><br>14+ d: B.1.351= 34.4% (-15.9-63.6)<br>Seronegative= 35.4%<br>14+ d: non-B.1.351= 82.1%<br>Seronegative= 68.3%	14+ d: in seronegative= 26.1%<br><br><br><br>Seronegative= 75.8%<br>14+ d: B.1.351= 15.8% (-63-56.9)<br>Seronegative= 23.5%
P.M. McKeigue (2022) Scotland (12/20-09/21)	Test-negative, case-control study	Individuals across Scotland with records in the public health electronic database. (59,475 vaccinated and 185,826 unvaccinated)	N/A	Severe infection<br><br>Hospitalization	Not reported	14+ d: 91% (87-94)<br><br>14+ d: 86% (83-88)
C. Menni (2021) UK (12/20-03/21)	Prospective, cohort study.	General Public in England that is 16+ years old reporting data via the study app. (36,329 received 1 dose and 464,356 unvaccinated)	N/A	PCR test-positive infection	21-44 d: 60% (49-68%)	Not reported
P. Nordstrom (2022) Sweden (12/20-10/21)	Retrospective cohort study	Individuals across Sweden with data in the Swedish nationwide register. (1,274,214 vaccinated with Pfizer, 153,760 vaccinated with Moderna, and 713,987 unvaccinated)	N/A	PCR test-positive infection	Not reported	14+ d: 44% (36-52)
C Paris (2021) France (01/21-05/21)	Prospective cohort surveillance study	HCWs (825 vaccinated and 1,380 unvaccinated)	N/A	PCR test-positive infection	14+ d: 86.2% (82-96)<br>14+d: Origin= 91% (12-71)<br>14+ d: B.1.1.7= 56% (29-117)	Not reported
M. Perry (2022) Wales (12/20-07/21)	Retrospective cohort	Individuals aged 50+ with records in multiple national health databases. (810,771 vaccinated and 93,439 unvaccinated)	B.1.1.7, B.1.617.2, N/A	PCR test-positive infection<br><br>Hospitalization	Not reported	7+ d: 24.9% (15.4-33.3)<br><br>7+ d: 81.4% (71.5-87.9)
E. Poukka (2022) Finland (12/20-10/21)	Prospective cohort study	HCWs ages 16-69 years old (15,613 vaccinated = 853 one dose, 14,760 two doses and 43,449 unvaccinated)	B.1.617.2, non-B.1.617.2, N/A	PCR test-positive infection<br><br><br><br>Hospitalization	**21-41 d: 7% (-24-30)**<br>**21-41 d: non-B.1.617.2= 6% (-26-29)**<br>**21-41 d: B.1.617.2= 100% (N/A)**<br><br>**21-41 d: -17% (-156-47)**<br>**21-41 d: non-B.1.617.2= -13% (-149-49)**<br>**21-41 d: B.1.617.2= 100% (N/A)**	14-90 d: 89% (73-95)<br>14-90 d: **non-B.1.617.2= 100**% (N/A)<br>14-90 d: **B.1.617.2= 88**% (71-95)<br><br>14-90 d: 100% (N/A)<br>14-90 d: **non-B.1.617.2= 100**% (N/A)<br>14-90 d: **B.1.617.2= 100**% (N/A)

K.B. Pouwels (2021) UK (12/20-08/21)	Prospective cohort study, self-reported symptoms via household survey	Individuals aged 18+ (for B.1.1.7 results only) and aged 18-64 (for B.1.617.2 results) with records in the National immunisation system.<br>B.1.1.7(36,918 received 1 dose, 4,370 received 2 doses, 265,334 unvaccinated)<br>B.1.617.2 (31,230 received 1 dose, 146,259 received 2 doses, and 19,412 unvaccinated)	B.1.1.7, B.1.617.2	PCR test-positive infection<br><br>Symptomatic infection<br><br>Asymptomatic infection	**21+ d: B.1.1.7= 63% (55-69)**<br>**21+ d: B.1.617.2= 43% (31-52)**<br>**21+ d: B.1.617.2= no prior= 43% (30-54)**<br>**21+ d: B.1.617.2= prior= 53% (24-71)**<br><br>**21+ d: B.1.1.7= 73% (67-77)**<br>**21+ d: B.1.617.2= 36% (23-47)**<br>**21+ d: B.1.617.2= no prior= 34% (16-48)**<br>**21+ d: B.1.617.2= prior= 83% (55-94)**<br><br>**21+ d: B.1.617.2= 50% (40-58)**	14+ d: B.1.1.7= 79% (56-90)<br>14+ d: B.1.617.2= 67% (62-71)<br>**21+ d: B.1.617.2= no prior= 68% (61-73)**<br>**21+ d: B.1.617.2= prior= 88% (83-92)**<br><br>14+ d: B.1.1.7= 97% (93-98)<br>14+ d: B.1.617.2= 70% (66-74)<br>**21+ d: B.1.617.2= no prior= 72% (64-78)**<br>**21+ d: B.1.617.2= prior= 94% (89-97)**<br><br>14+ d: B.1.617.2= 57% (51-63)
E. Pritchard (2021) UK (12/20- 05/21)	Prospective cohort Infection Survey.	Community individuals 16 years and above with data in the Office for National Statistics.<br>(41,018 received 2 doses, 144,859 received 1 dose and 329,419 non vaccinated)	B.1.1.7, N/A	PCR test-positive infections	21+ d: 61% (54-68)	7-30 d: 79% (65-88)
P. Sritipsukho (2022) Thailand (07/21-10/21)	Test-negative case-control study	Individuals 18+ years old admitted to hospitals with data records in an electronic health system<br>(750 vaccinated with 1 dose, 147 vaccinated with 2 doses, and 974 unvaccinated)	B.1.617.2, N/A	PCR test-positive infection	Not reported	14+ d: 83% (70-90)
J.L. Suah [A](2021) Malaysia (04/21-09/21)	Retrospective cohort study	Individuals aged 18+ in Malaysia with records in the Ministry of health<br>(45,736 received 1 dose, 4,687 received 2 doses, and 749,524 unvaccinated)	N/A	ICU admission<br><br>Death	14+ d: 60% (55.6-64)<br><br>14+ d: 70.7% (67.3-73.7)	14+ d: 95.6% (88.3-98.4)<br><br>14+ d: 95.3% (91.3-97.4)
E. Vasileiou (2021) Scotland (12/20- 02/21)	Prospective, cohort study	Individuals 18 years and older with records in multiple national healthcare databases.<br>(620 154 vaccinated; 111,526 aged 18-64, 336,304 aged 65-79, 172,324 >80.<br>743,142 unvaccinated: 626,849 aged 18-64,<br>81,523 aged 65-79,<br>35,266 aged 80+)	N/A	Hospital admissions	Total population:<br>14-20 d: 73% (66-79)<br>21-27 d:81% (72-87)	Not reported

Z. Voko (2022) Hungary (01/21-06/21)	Prospective cohort study	Individuals 16+ years old in Hungary with records in the National Public Health Centre. (304,138 vaccinated and 3,665 unvaccinated per 100,000 person days	N/A, B.1.1.7	PCR test-positive infection<br><br>Death	Not reported	7+ d: 71.5% (69.2-73.6)<br>14+ d: 73.7% (71.1-76)<br>28+ d: 73.4% (69.5-76.7)<br><br>7+ d: 88.3% (78.7-93.5)<br>14+ d: 85.8% (73.5-92.4)<br>28+ d: 77% (55.6-88)

Supplementary Table 3. Vaccine Efficacy reported within study by age (years) of the participant

Study (Publication Year)	Vaccine	Outcome(s) Measured	Adjusted vaccine Efficacy (95% CI)	
			After dose 1	After dose 2
G. Amirthalingam (2021)	Pfizer, AstraZeneca	Test positive infection	(AstraZeneca) 14-27 d: ages 65-79= 33% (27-39)<br>(AstraZeneca) 28+ d: ages 65-79= 52% (46-56)	(AstraZeneca) 14+ d: ages 65-79 (45-64 d between dose)= 61% (47-70)<br>(AstraZeneca) 14+ d: ages 50-64 (45-64 d between dose)= 70% (66-74)
N. Andrews[B] (2022)	Pfizer, Moderna and Astra Zeneca	Symptomatic infection	(Pfizer) 2-9 wk: B.1.617.2, ages 16-39= 52.5% (52.1-53)<br>(Pfizer) 2-9 wk: B.1.617.2, ages 40-64= 43.9% (41.9-45.8)<br>(Pfizer) 2-9 wk: B.1.617.2, ages 65+= 53.8% (43.8-62)<br>(Pfizer) 2-9 wk: B.1.1.7, ages 40-64= 49.4% (45.6-53)<br>(Pfizer) 2-9 wk: B.1.1.7, ages 65+= 54.8% (50.5-58.7)<br>(Pfizer) 2-9 wk: B.1.1.7, ages 80+= 56% (47.5-63.1)<br>(Moderna) 2-9 wk: B.1.617.2, ages 16-39= 66.2% (65.3-67)	(Pfizer) 2-9 wk: B.1.617.2, ages 65+= 79.6% (77-81.8)<br>(Pfizer) 10-14 wk: B.1.617.2, ages 65+= 69.4% (66.7-71.8)<br>(Pfizer) 15-19 wk: B.1.617.2, ages 65+= 63.1% (60.2-65.9)<br>(Pfizer) 20+ wk: B.1.617.2, ages 65+= 54.9% (51.1-58.5)<br>(Pfizer) 25+ wk: B.1.617.2, ages 65+= 51.8% (45.4-57.4)<br>(Pfizer) 2-9 wk: B.1.617.2, ages 40-64= 84.3% (83.7-84.8)<br>(Pfizer) 10-14 wk: B.1.617.2, ages 40-64= 77.3% (76.6-78)

		Hospitalization	(Moderna) 2-9 wk: B.1.617.2, ages 40-64= 53.3% (49.9-56.5)<br>(Moderna) 2-9 wk: B.1.1.7, ages 40-64= 67.5% (12.7-87.9)<br>(AstraZeneca) 2-9 wk: B.1.617.2, ages 16-39= 49.3% (45.7-52.7)<br>(AstraZeneca) 2-9 wk: B.1.617.2, ages 40-64= 33.4% (31.8-34.9)<br>(AstraZeneca) 2-9 wk: B.1.617.2, ages 65+= 40.7% (31.4-48.8)<br>(AstraZeneca) 2-9 wk: B.1.1.7, ages 40-64= 49.9% (47.2-52.5)<br>(AstraZeneca) 2-9 wk: B.1.1.7, ages 65+= 54.8% (50.2-58.9)	(Pfizer) 15-19 wk: B.1.617.2, ages 40-64= 72.1% (71.2-72.9)<br>(Pfizer) 20+ wk: B.1.617.2, ages 40-64= 69.2% (67.7-70.6)<br>(Pfizer) 2-9 wk: B.1.617.2, ages 16-39= 89.5% (89.3-89.7)<br>(Pfizer) 10-14 wk: B.1.617.2, ages 16-39= 72.9% (71.5-74.1)<br>(Pfizer) 15-19 wk: B.1.617.2, ages 16-39= 69.8% (52.4-80.8)<br>(Pfizer) 2-9 wk: B.1.1.7, ages 80+= 81.7% (76.1-86)<br>(Pfizer) 10+ wk: B.1.1.7, ages 80+= 67.1% (31.1-84.3)<br>(Pfizer) 2-9 wk: B.1.1.7, ages 65+= 94.2% (90.3-96.6)<br>(Pfizer) 2-9 wk: B.1.1.7, ages 40-64= 91.9% (86.5-95.2)<br>(Moderna) 2-9 wk: B.1.617.2, ages 40-64= 92.1% (91.3-92.8)<br>(Moderna) 10-14 wk: B.1.617.2, ages 40-64= 87.3% (85.5-88.8)<br>(Moderna) 2-9 wk: B.1.617.2, ages 16-39= 93.5% (93-94)<br>(Moderna) 10-14 wk: B.1.617.2, ages 16-39= 82.1% (75.2-87.1)<br>(AstraZeneca) 2-9 wk: B.1.617.2, ages 65+= 59.1% (55.4-62.6)<br>(AstraZeneca) 10-14 wk: B.1.617.2, ages 65+= 50.1% (46.1-53.8)<br>(AstraZeneca) 15-19 wk: B.1.617.2, ages 65+= 43.8% (39.3-47.9)<br>(AstraZeneca) 20+ wk: B.1.617.2, ages 65+= 38% (32.7-42.8)

		Death	(Pfizer) 2-9 wk: B.1.617.2, ages 16-39= 90.7% (89-92.1)<br>(Pfizer) 2-9 wk: B.1.617.2, ages 40-64= 93% (89.5-95.4)<br>(Pfizer) 2-9 wk: B.1.617.2, ages 65+= 95.4% (85.4-98.6)<br>(Pfizer) 2-9 wk: B.1.1.7, ages 40-64= 91.8% (83.3-96)<br>(Pfizer) 2-9 wk: B.1.1.7, ages 65+= 79.2% (71.2-84.9)<br>(Pfizer) 2-9 wk: B.1.1.7, ages 80+= 76.1% (61.9-85)<br>(Moderna) 2-9 wk: B.1.617.2, ages 16-39= 93.9% (89.5-96.5)<br>(Moderna) 2-9 wk: B.1.617.2, ages 40-64= 93.6% (82.9-97.6)<br>(AstraZeneca) 2-9 wk: B.1.617.2, ages 16-39= 86.1% (66.5-94.2)<br>(AstraZeneca) 2-9 wk: B.1.617.2, ages 40-64= 83.2% (80.3-85.7)<br>(AstraZeneca) 2-9 wk: B.1.617.2, ages 65+= 81.6% (69-89)<br>(AstraZeneca) 2-9 wk: B.1.1.7, ages 40-64= 79.8% (71.2-885.8)<br>(AstraZeneca) 2-9 wk: B.1.1.7, ages 65+= 79.8% (70.9-86)	(AstraZeneca) 25+ wk: B.1.617.2, ages 65+= 27.8% (16.3-37.8)<br>(AstraZeneca) 2-9 wk: B.1.617.2, ages 40-64= 62% (61.3-62.6)<br>(AstraZeneca) 10-14 wk: B.1.617.2, ages 40-64= 57.4% (56.6-58.2)<br>(AstraZeneca) 15-19 wk: B.1.617.2, ages 40-64= 55.4% (54.4-56.3)<br>(AstraZeneca) 20+ wk: B.1.617.2, ages 40-64= 56.7% (55-58.4)<br>(AstraZeneca) 2-9 wk: B.1.1.7, ages 65+= 88.2% (82.2-92.1)<br>(AstraZeneca) 2-9 wk: B.1.1.7, ages 40-64= 81.7% (76-86.1)<br><br>(Pfizer) 2-9 wk: B.1.617.2, ages 65+= 98% (95.9-99.1)<br>(Pfizer) 10-14 wk: B.1.617.2, ages 65+= 95.8% (94.4-96.9)<br>(Pfizer) 15-19 wk: B.1.617.2, ages 65+= 93.4% (91.6-94.7)<br>(Pfizer) 20+ wk: B.1.617.2, ages 65+= 90.5% (87.6-92.7)<br>(Pfizer) 2-9 wk: B.1.617.2, ages 40-64= 98.6% (97.9-99.1)<br>(Pfizer) 10-14 wk: B.1.617.2, ages 40-64= 97.7% (96.9-98.3)<br>(Pfizer) 15-19 wk: B.1.617.2, ages 40-64= 96.5% (95.3-97.4)<br>(Pfizer) 20+ wk: B.1.617.2, ages 40-64= 93.8% (87.5-96.9)

			(Pfizer) 2-9 wk: B.1.617.2, ages 65+= 81.5% (55.5-92.3)<br>(Pfizer) 2-9 wk: B.1.1.7, ages 65+= 80.4% (73.8-85.3)<br>(AstraZeneca) 2-9 wk: B.1.617.2, ages 65+= 82.5% (57.9-92.7)<br>(AstraZeneca) 2-9 wk: B.1.1.7, ages 65+= 82.9% (73.1-89.1)	(Pfizer) 2-9 wk: B.1.617.2, ages 16-39= 99.2% (98.3-99.6)<br>(Pfizer) 10-14 wk: B.1.617.2, ages 16-39= 100% (N/R)<br>(Pfizer) 2-9 wk: B.1.1.7, ages 80+= 92.6% (81.6-97)<br>(Pfizer) 10+ wk: B.1.1.7, ages 80+= 87.8% (-15.4-98.7)<br>(Pfizer) 2-9 wk: B.1.1.7, ages 65+= 100% (N/R)<br>(Pfizer) 2-9 wk: B.1.1.7, ages 40-64= 82.1% (21.6-95.9)<br>(Pfizer) 10+ wk: B.1.1.7, ages 40-64= 100% (N/R)<br>(AstraZeneca) 2-9 wk: B.1.617.2, ages 65+= 91.7% (88.8-93.9)<br>(AstraZeneca) 10-14 wk: B.1.617.2, ages 65+= 90.1% (87.7-92)<br>(AstraZeneca) 15-19 wk: B.1.617.2, ages 65+= 85.8% (82.7-88.4)<br>(AstraZeneca) 20+ wk: B.1.617.2, ages 65+= 81.8% (76.7-85.9)<br>(AstraZeneca) 2-9 wk: B.1.617.2, ages 40-64= 96.2% (95.7-96.6)<br>(AstraZeneca) 10-14 wk: B.1.617.2, ages 40-64= 93.2% (92.4-94)<br>(AstraZeneca) 15-19 wk: B.1.617.2, ages 40-64= 89.9% (88.1-91.4)<br>(AstraZeneca) 20+ wk: B.1.617.2, ages 40-64= 79.1% (70.3-85.3)<br>(AstraZeneca) 2-9 wk: B.1.1.7, ages 65+= 96.8% (71.8-99.6)<br>(AstraZeneca) 10+ wk: B.1.1.7, ages 65+= 100% (N/R)<br>(AstraZeneca) 2-9 wk: B.1.1.7, ages 40-64= 83% (40.5-95.2)<br>(AstraZeneca) 10+ wk: B.1.1.7, ages 40-64= 100% (N/R)

				(Pfizer) 2-9 wk: B.1.617.2, ages 65+= 97.1% (91.7-99)<br>(Pfizer) 10-14 wk: B.1.617.2, ages 65+= 95.1% (92.1-96.9)<br>(Pfizer) 15-19 wk: B.1.617.2, ages 65+= 93.2% (90.1-95.4)<br>(Pfizer) 20+ wk: B.1.617.2, ages 65+= 90.2% (85.3-93.5)<br>(Pfizer) 2+ wk: B.1.1.7, ages 65+= 96.6% (90.2-98.8)<br>(AstraZeneca) 2-9 wk: B.1.617.2, ages 65+= 94.1% (89.6-96.7)<br>(AstraZeneca) 10-14 wk: B.1.617.2, ages 65+= 92.9% (89.5-95.2)<br>(AstraZeneca) 15-19 wk: B.1.617.2, ages 65+= 87.9% (82.6-91.5)<br>(AstraZeneca) 20+ wk: B.1.617.2, ages 65+= 82.1% (70.1-89.3)
K.J. Bruxvoort (2021)	Moderna	PCR test positive infection	Not reported	14+ d: B.1.617.2, Age 18-64= 87.9% (85.5-89.9)<br>14-60 d: B.1.617.2, age 18-64= 95.1% (91.8-97.1)<br>151-180 d: B.1.617.2, age 18-64= 79.4% (68.8-86.3)<br>14+ d: B.1.617.2, age 65+= 75.2% (59.6-84.8)
A.A. Butt (2021)	Pfizer and Moderna	PCR test positive	Ages <70: 86.8% (85.6-87.9)<br>Ages 70+: 84.3% (83.2-85.1)	7+ d: ages <70= 96.4% (95.3-97.2)<br>7+ d: ages 70+= 97.5% (96.9-97.9)
T. Cerqueira-Silva (2022	Pfizer and AstraZeneca	Symptomatic infection	Not reported	(Pfizer) 14+ d: age 18-49= 64.6% (53-73.3)<br>(Pfizer) 14+ d: age 50+= 66.9% (46-79.7)<br>(AstraZeneca) 14+ d: age 18-49= 54.8% (49.4-59.7)<br>(AstraZeneca) 14+ d: age 50+= 59.9% (50.8-67.3)<br><br>(Pfizer) 14+ d: age 18-49= 88.6% (6.3-98.6)

		Hospitalization		(Pfizer) 14+ d: age 50+= 89.8% (17-98.7)<br>(AstraZeneca) 14+ d: age 18-49= 88.7% (73.9-95.1)<br>(AstraZeneca) 14+ d: age 50+= 89.9% (81.4-94.5)
C. Chodick (2021)	Pfizer	PCR test positive	Not reported	7-27 d: ages 16-44= 92% (83-96)<br>7-27 d: ages 45-64= 90% (80-95)<br>7-27 d: ages 65-74= 82% (63-92)<br>7-27 d: ages 75+= 82% (61-91)
H. Chung (2021)	Pfizer and Moderna	Symptomatic	28-34 d: age 70+= 64% (46-76)<br>42-48 d: age 70+= 85% (38-97)	Not reported
B.A Cohn (2021)	Pfizer and Moderna	Death	Not reported	(Pfizer) 14+ d: B.1.617.2, age <65= 84.3% (76.3-89.7)<br>(Pfizer) 14+ d: B.1.617.2, age 65+= 70.1% (66.1-73.6)<br>(Moderna) 14+ d: B.1.617.2, age <65= 81.5% (70.7-88.4)<br>(Moderna) 14+ d: B.1.617.2, age 65+= 75.5% (71.8-78.7)
N. Dagan (2021)[A]	Pfizer	PCR test positive infection	14-20 d: age 16-39= 49% (41-57)<br>21-27 d: age 16-39= 64% (54-72)<br>14-20 d: age 40-69= 47% (40-55)<br>21-27 d: age 40-69= 58% (49-67)<br>14-20 d: age 70+= 22% (-9-44)<br>21-27 d: age 70+= 50% (19-72)	7+ d: age 16-39= 94% (87-97)<br>7+ d: age 40-69= 90% (82-95)<br>7+ d: age 70+= 95% (87-100)
		Symptomatic infection	14-20 d: age 16-39= 57% (46-68)<br>21-27 d: age 16-39= 67% (52-78)<br>14-20 d: age 40-69= 59% (50-67)<br>21-27 d: age 40-69= 65% (53-74)<br>14-20 d: age 70+= 44% (19-64)<br>21-27 d: age 70+= 64% (37-83)	7+ d: age 16-39= 99% (96-100)<br>7+ d: age 40-69= 90% (75-98)<br>7+ d: age 70+= 98% (90-100)
H.M. El Sahly (2021)	Moderna	Symptomatic infection	Not reported	14+ d: ages 18-65= 93.4% (91.1-95.1)<br>14+ d: ages 65+= 91.5% (83.2-95.7)<br>14+ d: ages 65-75= 89.7% (79.6-94.9)

				14+ d: ages 75+= 100% (N/R)
A.R. Falsey (2021)	AstraZeneca	Symptomatic infection	Not reported	15+ d: ages 18-64= 72.8% (63.4-79.9)<br>15+ d: ages 65+= 83.5% (54.2-94.1)
A. Florea (2022)	Pfizer	PCR positive infection<br><br><br><br><br><br><br><br><br><br><br><br><br>Hospitalization	Not reported	14+ d: Age 18-44= 82.6% (81.7-83.5)<br>14+ d: Age 45-64= 84.6% (83.8-85.4)<br>14+ d: age 65-74= 81.6% (79.9-83.1)<br>14+ d: Age 75+= 77.9% (75.1-80.3)<br>0-2 mo: age 18-64= 88.4% (87.1-89.6)<br>2-4 mo: age 18-64= 84.6% (83.9-85.4)<br>4-6 mo: age 18-64= 77.6% (76-79)<br>6-8 mo: age 18-64= 71.6% (54.7-82.2)<br>0-2 mo: age 65+= 86.5% (83.2-89.2)<br>2-4 mo: age 65+= 84.2% (81.7-86.3)<br>4-6 mo: age 65+= 76.8% (74.5-78.8)<br>6-8 mo: age 65+= 77.7% (72.3-82.1)<br><br>0-2 mo: age 18-64= 96.2% (93-98)<br>2-4 mo: age 18-64= 98.1% (97.3-98.7)<br>4-6 mo: age 18-64= 96.6% (95-97.8)<br>6-8 mo: age 18-64= 98.7% (78.6-99.9)<br>0-2 mo: age 65+= 95.2% (90.6-97.5)<br>2-4 mo: age 65+= 95% (92.4-96.7)<br>4-6 mo: age 65+= 93.2% (91.4-94.6)<br>6-8 mo: age 65+= 93.8% (89.8-96.3)
Y. Goldberg (2022)	Pfizer	PCR test positive infection by million-person year	15+ d: age 16-39= 73.9% (73.2-74.5)<br>15+ d: age 40-49= 64.5% (63.3-65.6)<br>15+ d: age 50-59= 60.5% (59.2-61.8)<br>15+ d: age 60-69= 58.9% (57.3-60.3)<br>15+ d: age 70-79= 64.7% (62.9-66.4)<br>15+ d: age 80+= 45.6% (42.4-48.5)	14+ d: age 16-39= 95.7% (95.3-96)<br>14+ d: age 40-49= 93.7% (93.2-94.2)<br>14+ d: age 50-59= 94.7% (94.2-95.1)<br>14+ d: age 60-69= 94.9% (94.4-95.3)<br>14+ d: age 70-79= 94.2% (93.6-94.7)<br>14+ d: age 80+= 90.3% (89.2-91.3)

		Hospitalization<br><br><br><br><br><br><br>Severe disease<br><br><br><br><br>Death	15+ d: age 16-39= 86.7% (82.6-89.8)<br>15+ d: age 40-49= 86.1% (82-89.4)<br>15+ d: age 50-59= 84.1% (80.8-86.9)<br>15+ d: age 60-69= 76.7% (73.4-79.5)<br>15+ d: age 70-79= 76% (73.2-78.5)<br>15+ d: age 80+= 61.1% (57.2-64.6)<br><br>15+ d: age 60-69= 78.1% (74.3-81.3)<br>15+ d: age 70-79= 77.9% (74.8-80.6)<br>15+ d: age 80+= 62.4% (58.1-66.2)<br><br>15+ d: age 60-69= 70.9% (61.4-78.1)<br>15+ d: age 70-79= 77.3% (72-81.6)<br>15+ d: age 80+= 63.4% (57.7-68.3)	14+ d: age 16-39= 95.4% (91.7-97.5)<br>14+ d: age 40-49= 94.3% (91.1-96.3)<br>14+ d: age 50-59= 95.8% (93.9-97.1)<br>14+ d: age 60-69= 97.2% (96.1-97.9)<br>14+ d: age 70-79= 96.2% (95.2-96.9)<br>14+ d: age 80+= 94.5% (93.3-95.5)<br><br>14+ d: age 60-69= 97.1% (95.8-98)<br>14+ d: age 70-79= 96.8% (95.8-97.6)<br>14+ d: age 80+= 95.4% (94.1-96.4)<br><br>14+ d: age 60-69= 95.3% (91.6-97.4)<br>14+ d: age 70-79= 96.9% (95.1-98)<br>14+ d: age 80+= 95.8% (94.1-97)
G.N. Ioannou (2022)	Pfizer and Moderna	PCR positive<br><br><br><br>Death	Not reported	7+ d: ages 18-64= 75% (72-77)<br>7+ d: ages 65-74= 72% (70-74)<br>7+ d: ages 74+= 61% (58-64)<br><br>7+ d: ages 18-64= 89% (62-97)<br>7+ d: ages 65-74= 84% (77-89)<br>7+ d: ages 75+= 87% (82-90)
S.V. Katikireddi (2022)	AstraZeneca	Hospitalization/Death	2+ wk: age 18-64= 57% (51.2-62.1)<br>2+ wk: age 65-79= 34.9% (12.5-51.6)<br>2+ wk: age 80+= -26.5% (-85.1-13.5)	2-3 wk: age 18-64= 87% (83.4-89.9)<br>4-5 wk: age 18-64= 89.5% (86.8-91.7)<br>6-7 wk: age 18-64= 90.5% (88-92.4)<br>8-9 wk: age 18-64= 84.6% (81.4-87.2)<br>10-11 wk: age 18-64= 83.3% (80-86.1)<br>12-13 wk: age 18-64= 83.2% (80-85.9)<br>14-15 wk: age 18-64= 79.4% (75.9-82.4)<br>16-17 wk: age 18-64= 74.6% (70.4-78.3)<br>18-19 wk: age 18-64= 65.9% (60-70.9)<br>20-21 wk: age 18-64= 51.1% (41.9-58.9)

				2-3 wk: age 65-79= 58.8% (24.7-77.5)<br>4-5 wk: age 65-79= 81.8% (68-89.6)<br>6-7 wk: age 65-79= 78.1% (66.7-85.6)<br>8-9 wk: age 65-79= 64.9% (52.4-74.1)<br>10-11 wk: age 65-79= 71.6% (62-78.8)<br>12-13 wk: age 65-79= 64.3% (53-78.8)<br>14-15 wk: age 65-79= 64.1% (52.8-72.7)<br>16-17 wk: age 65-79= 67.5% (58.4-74.6)<br>18-19 wk: age 65-79= 64.9% (56.8-71.4)<br>20-21 wk: age 65-79= 65.5% (57.9-71.8)<br><br>2-3 wk: age 80+= 33.3% (-179.7-84.1)<br>4-5 wk: age 80+= -7.6% (-123.8-48.3)<br>6-7 wk: age 80+= 33.5% (-22.5-64)<br>8-9 wk: age 80+= 30.2% (-11.2-56.2)<br>10-11 wk: age 80+= 35% (4.1-55.9)<br>12-13 wk: age 80+= 30.9% (1.9-51.3)<br>14-15 wk: age 80+= 47.2% (24.7-62.9)<br>16-17 wk: age 80+= 32.1% (5.3-51.3)<br>18-19 wk: age 80+= 36.9% (14.5-53.4)<br>20-21 wk: age 80+= 20.6% (-3.8-39.2)
A.S Lauring (2022)	Pfizer and Moderna	Hospitalization	Not reported	14+ d: B.1.1.7 ages 18-64= 87% (82-91)<br>14+ d: B.1.1.7 ages 65+= 85% (80-89)<br>14+ d: B.1.617.2 ages 18-64=88% (86-89)<br>14+ d: B.1.617.2 ages 65+= 81% (77-84)
S.M. Olson (2022)	Pfizer	Hospitalization	Not reported	14+ d: ages 12-15= 95% (89-98)<br>14+ d: ages 16-18= 96% (90-98)
J. Pardo-Seco (2022)	Pfizer	PCR test positive infection	7-13 d: ages 18-64= 63.8% (59.7-67.7)<br>14-20 d: ages 18-64= 74.7% (71-78)<br>7-13 d: ages 65-79= 62.9% (53.2-71.2)	7-13 d: ages 18-64= 79% (71.7-84.9)<br>14+ d: ages 18-64= 92.9% (90.2-95.1)<br>7-13 d: ages 65-79= 83% (71.2-90.9)

		Hospitalization	14-20 d: ages 65-79= 57.4% (46.1-66.9)<br>7-13 d: ages 80+= 57.9% (50.8-64.1)<br>14-20 d: ages 80+= 51.4% (43.5-58.4)<br><br>14+ d: ages 18-64= 46% (21.7-64.8)<br>14+ d: ages 65-79= 53% (30.3-70.3)<br>14+ d: ages 80+= 60% (49.4-68.3)	14-20 d: ages 65-79= 85.8% (77.3-91.9)<br>7-13 d: ages 80+= 78.8% (71.7-85.6)<br>14-20 d: ages 80+= 91.4% (87.9-94.1)
T. Pilishvili (2021)	Pfizer and Moderna	PCR positive infection	14+ d: ages <50= 80.3% (74.2-85)<br>14+ d: ages >50= 77% (62.7-85.8)	7+ d: ages <50= 90.3% (86.5-93)<br>7+ d: ages >50= 90.7% (84.2-94.6)
F.P. Polack (2021)	Pfizer	Symptomatic infection	Not reported	7+ d: ages 16-64= 94.2% (84.4-98.5)<br>7+ d: ages ≥65= 100% (29-100)
K.B. Pouwels (2021)	Pfizer and AstraZeneca	PCR positive<br><br>Symptomatic infection	(Pfizer) 21+ d: B.1.617.2, age 18-34= 64% (57-70)<br>(Pfizer) 21+ d: B.1.617.2, age 35-64= 36% (14-53)<br>(AstraZeneca) 21+ d: B.1.617.2, age 18-34= 43% (11-64)<br>(AstraZeneca) 21+ d: B.1.617.2, age 35-64= 18% (-9-39)<br>(Moderna) 21+ d: B.1.617.2, age 18-34= 82% (70-89)<br>(Moderna) 21+ d: B.1.617.2, age 35-64= 56% (23-75)<br><br>(Pfizer) 21+ d: B.1.617.2, age 18-34= 66% (58-72)<br>(Pfizer) 21+ d: B.1.617.2, age 35-64= 32% (0-53)<br>(AstraZeneca) 21+ d: B.1.617.2, age 18-34= 37% (-7-63)	(Pfizer) 14+ d: B.1.617.2, age 18-34=90% (85-93)<br>(Pfizer) 14+ d: B.1.617.2, age 35-64= 77% (65-85)<br>(AstraZeneca) 14+ d: B.1.617.2, age 18-34=73% (65-80)<br>(AstraZeneca) 14+ d: B.1.617.2, age 35-64= 54% (40-65)<br><br>(Pfizer) 14+ d: B.1.617.2, age 18-34=96% (93-98)<br>(Pfizer) 14+ d: B.1.617.2, age 35-64= 88% (78-94)<br>(AstraZeneca) 14+ d: B.1.617.2, age 18-34=76% (67-83)<br>(AstraZeneca) 14+ d: B.1.617.2, age 35-64= 57% (39-70)

			(AstraZeneca) 21+ d: B.1.617.2, age 35-64= 0% (-43-31)<br>(Moderna) 21+ d: B.1.617.2, age 18-34= 85% (72-92)<br>(Moderna) 21+ d: B.1.617.2, age 35-64= 48% (-1-73)	
D.M. Skowronski (2021)	Pfizer and Moderna	PCR positive	14-20 d: age 70-79= 46% (32-57)<br>21-27 d: age 70-79= 61% (47-71)<br>28-34 d: age 70-79= 77% (64-85)<br>35-41 d: age 70-79= 73% (51-85)<br>42+ d: age 70-79= 63% (38-78)<br>21+ d: age 70-79= 67% (59-74)<br>14-20 d: age 80-89= 39% (9-59)<br>21-27 d: age 80-89= 54% (32-70)<br>28-34 d: age 80-89= 64% (44-77)<br>35-41 d: age 80-89= 75% (55-86)<br>42+ d: age 80-89= 66% (39-81)<br>21+ d: age 80-89= 65% (52-75)<br>14-20 d: age 80+= 36% (6-56)<br>21-27 d: age 80+= 50% (27-66)<br>28-34 d: age 80+= 59% (39-73)<br>35-41 d: age 80+= 76% (59-86)<br>42+ d: age 80+= 64% (39-78)<br>21+ d: age 80+= 62% (49-72)	Not reported

J.L. Suah (2022)	Pfizer	PCR test positive infection<br><br><br><br><br><br><br><br>ICU admission<br><br><br><br><br><br><br><br>Death	Not reported	14 d-2 mo: ages 15-39= 91.9% (90.2-93.3)<br>3-5 mo: ages 15-39= 86.1% (83.2-88.5)<br>14 d-2 mo: ages 40-59= 88.9% (86.5-90.9)<br>3-5 mo: ages 40-59= 77.3% (72.3-81.3)<br>14 d-2 mo: ages 60+= 90.9% (89.1-92.4)<br>3-5 mo: ages 60+= 69.8% (63.7-74.9)<br><br>14 d-2 mo: ages 18-39= 94.9% (88.3-97.7)<br>3-5 mo: ages 18-39= 92.3% (83.6-96.6)<br>14 d-2 mo: ages 40-59= 90.2% (86.2-93)<br>3-5 mo: ages 40-59= 89.9% (83.3-93.9)<br>14 d-2 mo: ages 60+= 83.8% (78.5-87.8)<br>3-5 mo: ages 60+= 57.1% (43.2-67.6)<br><br>14 d-2 mo: ages 18-39= 94.3% (84.3-97.9)<br>3-5 mo: ages 18-39= 94.4% (80.3-98.4)<br>14 d-2 mo: ages 40-59= 94.5% (91.7-96.3)<br>3-5 mo: ages 40-59= 94.6% (89-97.4)<br>14 d-2 mo: ages 60+= 90.4% (88.2-92.3)<br>3-5 mo: ages 60+= 89.4% (86-92)
P. Tang (2021)	Pfizer and Moderna	PCR test infection<br><br><br><br><br><br>Severe, critical, fatal infection	(Pfizer) 14+ d: age <50= 49.8% (28.2-64.9)<br>(Moderna) 14+ d: age <50= 81% (68.7-88.5)<br>14+ d: age <50= 65.7% (54-74.4)	(Pfizer) 14+ d: age <50= 55.1% (50.7-59.1)<br>(Moderna) 14+ d: age <50= 77.5% (72.5-81.6)<br>14+ d: age <50= 57.8% (54-61.4)<br>Pfizer) 14+ d: age >50= 16.5% (-21.4-42.6)<br>14+ d: age >50= 29.5% (-0.1-50.4)<br><br>(Pfizer) 14+ d: age <50= 96.2% (84.2-99.1)<br>14+ d: age <50= 94.8% (83.2-98.4)

S.Y Tartof (2022)	Pfizer	PCR positive infection	14+ d: age 12-15= 67% (50-78)<br>14+ d: age 16-44= 62% (57-66)<br>14+ d: age 45-64= 55% (49-61)<br>14+ d: age 65+= 46% (33-57)<br>14+ d: age 16+= 57% (53-61)	7+ d: age 12-15= 91% (88-93)<br>7+ d: age 16-44= 73% (71-74)<br>7+ d: age 45-64= 73% (71-74)<br>7+ d: age 65+= 61% (57-65)<br>7+ d: age 16+= 72% (71-73)<br>7-36 d: age 12-15= 91% (86-94)<br>37-66 d: age 12-15= 92% (88-94)<br>67-96 d: age 12-15= 88% (68-96)<br>97-126 d: age 12-15= 84% (-14-98)<br>127-156 d: age 12-15= 100% (100-100)<br>157+ d: age 12-15= 100% (100-100)<br>7-36 d: age 16-44= 89% (87-91)<br>37-66 d: age 16-44= 84% (81-86)<br>67-96 d: age 16-44= 78% (75-80)<br>97-126 d: age 16-44= 68% (65-71)<br>127-156 d: age 16-44= 57% (51-62)<br>157+ d: age 16-44= 39% (32-45)<br>7-36 d: age 45-64= 87% (84-90)<br>37-66 d: age 45-64= 85% (81-88)<br>67-96 d: age 45-64= 78% (74-81)<br>97-126 d: age 45-64= 67% (63-70)<br>127-156 d: age 45-64= 61% (55-66)<br>157+ d: age 45-64= 50% (43-57)<br>7-36 d: age 65+= 80% (73-85)<br>37-66 d: age 65+= 79% (70-85)<br>67-96 d: age 65+= 75% (65-83)<br>97-126 d: age 65+= 56% (45-65)<br>127-156 d: age 65+= 49% (41-57)<br>157+ d: age 65+= 43% (30-54)<br>7-36 d: age 16+= 87% (85-89)<br>37-66 d: age 16+= 84% (82-86)

				67-96 d: age 16+= 77% (76-79)<br>97-126 d: age 16+= 68% (65-70)<br>127-156 d: age 16+= 61% (58-64)<br>157+ d: age 16+= 47% (43-51)
		Hospitalization	14+ d: age 12-15= 100% (100-100)<br>14+ d: age 16-44= 74% (52-86)<br>14+ d: age 45-64= 78% (61-88)<br>14+ d: age 65+= 25% (1-43)<br>14+ d: age 16+= 54% (42-63)	7+ d: age 12-15= 81% (-55-98)<br>7+ d: age 16-44= 92% (88-95)<br>7+ d: age 45-64= 91% (88-93)<br>7+ d: age 65+= 86% (82-88)<br>7+ d: age 16+= 90% (89-92)<br>7-36 d: age 12-15= 100% (100-100)<br>37-66 d: age 12-15= 69% (-145-96)<br>67-96 d: age 12-15= 100% (100-100)<br>97-126 d: age 12-15= 100% (100-100)<br>127-156 d: age 12-15= 100% (100-100)<br>157+ d: age 12-15= 100% (100-100)<br>7-36 d: age 16-44= 88% (75-94)<br>37-66 d: age 16-44= 91% (80-96)<br>67-96 d: age 16-44= 98% (90-99)<br>97-126 d: age 16-44= 94% (85-98)<br>127-156 d: age 16-44= 88% (67-95)<br>157+ d: age 16-44= 90% (69-97)<br>7-36 d: age 45-64= 91% (82-96)<br>37-66 d: age 45-64= 86% (75-92)<br>67-96 d: age 45-64= 91% (83-95)<br>97-126 d: age 45-64= 94% (88-97)<br>127-156 d: age 45-64= 95% (84-98)<br>157+ d: age 45-64= 90% (75-96)<br>7-36 d: age 65+= 84% (74-90)<br>37-66 d: age 65+= 88% (78-93)

				67-96 d: age 65+= 89% (78-94)<br>97-126 d: age 65+= 86% (77-92)<br>127-156 d: age 65+= 85% (77-90)<br>157+ d: age 65+= 83% (69-90)<br>7-36 d: age 16+= 87% (82-91)<br>37-66 d: age 16+= 89% (84-92)<br>67-96 d: age 16+= 92% (89-95)<br>97-126 d: age 16+= 93% (89-95)<br>127-156 d: age 16+= 91% (87-93)<br>157+ d: age 16+= 88% (82-92)
S.Y Tartof (2022)	Pfizer	Hospitalization	Not reported	7+ d: B.1.617.2, age 18-64= 78% (68-85)<br>7+ d: B.1.1.529, age 18-64= 61% (48-71)<br>7 d-3 mo: B.1.617.2, age 18-64= 81% (55-92)<br>7 d-3 mo: B.1.1.52, 9, age 18-64= 60% (27-78)<br>3-6 mo: B.1.617.2, age 18-64= 90% (78-95)<br>3-6 mo: B.1.1.529, age 18-64= 62% (40-76)<br>6-9 mo: B.1.617.2, age 18-64= 77% (64-86)<br>6-9 mo: B.1.1.529, age 18-64= 69% (55-79)<br>9+ mo: B.1.617.2, age 18-64= 47% (-3-73)<br>9+ mo: B.1.1.529, age 18-64= 27% (-25-58)<br>7+ d: B.1.617.2, age 65+= 78% (65-86)<br>7+ d: B.1.1.529, age 65+= 64% (52-73)<br>7 d-3 mo: B.1.617.2, age 65+= 69% (2-90)<br>7 d-3 mo: B.1.1.529, age 65+= 81% (53-92)<br>3-6 mo: B.1.617.2, age 65+= 80% (44-93)<br>3-6 mo: B.1.1.529, age 65+= 76% (54-88)<br>6-9 mo: B.1.617.2, age 65+= 67% (39-82)<br>6-9 mo: B.1.1.529, age 65+= 76% (62-85)<br>9+ mo: B.1.617.2, age 65+= 86% (72-93)<br>9+ mo: B.1.1.529, age 65+= 50% (30-64)

		Emergency department admission		7+ d: B.1.617.2, age 18-64= 58% (51-64)<br>7+ d: B.1.1.529, age 18-64= 45% (36-52)<br>7 d-3 mo: B.1.617.2, age 18-64= 76% (65-84)<br>7 d-3 mo: B.1.1.529, age 18-64= 60% (45-71)<br>3-6 mo: B.1.617.2, age 18-64= 56% (43-65)<br>3-6 mo: B.1.1.529, age 18-64= 44% (30-55)<br>6-9 mo: B.1.617.2, age 18-64= 57% (47-64)<br>6-9 mo: B.1.1.529, age 18-64= 48% (38-56)<br>9+ mo: B.1.617.2, age 18-64= 53% (39-64)<br>9+ mo: B.1.1.529, age 18-64= 26% (6-42)<br>7+ d: B.1.617.2, age 65+= 75% (62-84)<br>7+ d: B.1.1.529, age 65+= 59% (44-70)<br>7 d-3 mo: B.1.617.2, age 65+= 92% (62-98)<br>7 d-3 mo: B.1.1.529, age 65+= 81% (52-92)<br>3-6 mo: B.1.617.2, age 65+= 80% (50-92)<br>3-6 mo: B.1.1.529, age 65+= 62% (30-79)<br>6-9 mo: B.1.617.2, age 65+= 74% (54-86)<br>6-9 mo: B.1.1.529, age 65+= 68% (51-79)<br>9+ mo: B.1.617.2, age 65+= 72% (54-83)<br>9+ mo: B.1.1.529, age 65+= 49% (27-65)
M.W. Tenforde (2021)	Pfizer and Moderna	Hospitalization	Not reported	14+ d: ages 18-49= 97.4% (79.3-99.7)<br>14+ d: ages 50-64= 75.2% (48.3-88.1)<br>14+ d: ages 65+= 87.3% (77.8-92.7)
S. J. Thomas (2021)	Pfizer	PCR test positive	19-23d: ages 16-55= 86.8% (83.6-89.5)<br>19-23d: ages 55+= 89.6% (85-92.9)<br>19-23d: ages 65+= 91.6% (84.8-95.7)<br>19-23d: ages 75+= 92.7% (70.7-99.2)	7+ d: ages 12+= 91.3% (89-93.2)
E. Vasileiou, (2021)	Pfizer and Astra Zeneca	Hospital Admission	(Pfizer) 14-20 d: ages 18-64= 42% (25-56)<br>(Pfizer) 21-27 d: ages 18-64= 63% (47-74)<br>(Pfizer) 28-34 d: ages 18-64= 92% (82-97)	Not reported

			(Pfizer) 35-41 d: ages 18-64= 63% (41-76)<br>(Pfizer) 42+ d: ages 18-64= 68% (53-78)<br>(Pfizer) 14-20 d: ages 65-79= 80% (66-88)<br>(Pfizer) 21-27 d: ages 65-79= 91% (73-97)<br>(Pfizer) 28-34 d: ages 65-79= 93% (73-98)<br>(Pfizer) 35-41 d: ages 65-79= 86% (57-95)<br>(Pfizer) 42+ d: ages 65-79= 76% (50-88)<br>(Pfizer) 14-20 d: ages 80+= 85% (76-90)<br>(Pfizer) 21-27 d: ages 80+= 85% (75-91)<br>(Pfizer) 28-34 d: ages 80+= 88% (76-94)<br>(Pfizer) 35-41 d: ages 80+= 87% (70-94)<br>(Pfizer) 42+ d: ages 80+= 85% (66-93)<br><br>(AstraZeneca) 14-20 d: ages 18-64= 75% (-6-94)<br>(AstraZeneca) 21-27 d: ages 18-64= 100% (NA-100)<br>(AstraZeneca) 14-20 d: ages 65-79= 79% (63-88)<br>(AstraZeneca) 21-27 d: ages 65-79= 68% (31-85)<br>(AstraZeneca) 28-34 d: ages 65-79= 100% (NA-100)<br>(AstraZeneca) 14-20 d: ages 80+= 63% (50-72)<br>(AstraZeneca) 21-27 d: ages 80+= 77% (63-86)<br>(AstraZeneca) 28-34 d: ages 80+= 81% (60-91)<br>(AstraZeneca) 35-41 d: ages 80+= 95% (44-100)	
Z. Voko (2022)	Pfizer, Moderna and Astra Zeneca	PCR positive infection		(Pfizer) 7+ d: ages 16-24= 82.3% (78.1-85.7)<br>(Pfizer) 7+ d: ages 25-34= 83.2% (80.8-85.2)<br>(Pfizer) 7+ d: ages 35-44= 84.2% (82.4-85.8)<br>(Pfizer) 7+ d: ages 45-54= 85.6% (84.3-86.9)<br>(Pfizer) 7+ d: ages 55-64= 85% (83.4-86.5)<br>(Pfizer) 7+ d: ages 65-74= 85.3% (83.5-86.9)<br>(Pfizer) 7+ d: ages 75-84= 82.1% (80.4-83.6)

				(Pfizer) 7+ d: ages 85+= 74.3% (71.4-76.8)<br>(Pfizer) 14+ d: ages 16-24= 82.6% (78.3-86.1)<br>(Pfizer) 14+ d: ages 25-34= 83.5% (81.1-85.6)<br>(Pfizer) 14+ d: ages 35-44= 84.1% (82.2-85.8)<br>(Pfizer) 14+ d: ages 45-54= 85.7% (84.3-87)<br>(Pfizer) 14+ d: ages 55-64= 85.8% (84.1-87.2)<br>(Pfizer) 14+ d: ages 65-74= 86.7% (84.9-88.4)<br>(Pfizer) 14+ d: ages 75-84= 83.3% (81.5-84.9)<br>(Pfizer) 14+ d: ages 85+= 75.2% (72.1-77.9)<br>(Pfizer) 28+ d: ages 16-24= 81.6% (76.6-85.5)<br>(Pfizer) 28+ d: ages 25-34= 82.7% (80-85)<br>(Pfizer) 28+ d: ages 35-44= 82.2% (80-84.1)<br>(Pfizer) 28+ d: ages 45-54= 84.9% (83.4-86.3)<br>(Pfizer) 28+ d: ages 55-64= 84.9% (83-86.5)<br>(Pfizer) 28+ d: ages 65-74= 86% (83.7-88)<br>(Pfizer) 28+ d: ages 75-84= 79.8% (77.3-82)<br>(Pfizer) 28+ d: ages 85+= 69.4% (65.1-73.2)<br>(Moderna) 7+ d: ages 16-24= 80.5% (39.4-93.7)<br>(Moderna) 7+ d: ages 25-34= 97% (78.6-99.6)<br>(Moderna) 7+ d: ages 35-44= 90.6% (79.1-95.8)<br>(Moderna) 7+ d: ages 45-54= 93.6% (86.7-97)<br>(Moderna) 7+ d: ages 55-64= 84.5% (75.7-90.1)<br>(Moderna) 7+ d: ages 65-74= 93.2% (88.8-95.8)<br>(Moderna) 7+ d: ages 75-84= 88.9% (84.5-92)<br>(Moderna) 7+ d: ages 85+= 84.1% (79-87.9)<br>(Moderna) 14+ d: ages 16-24= 70.8% (9.5-90.6)<br>(Moderna) 14+ d: ages 25-34= 100% (N/R)<br>(Moderna) 14+ d: ages 35-44= 87.9% (71-95)<br>(Moderna) 14+ d: ages 45-54= 91.8% (81.7-96.3)<br>(Moderna) 14+ d: ages 55-64= 85.4% (74.3-91.7)<br>(Moderna) 14+ d: ages 65-74= 93.1% (87.6-96.2)

				(Moderna) 14+ d: ages 75-84= 88.8% (83.5-92.4)<br>(Moderna) 14+ d: ages 85+= 84.4% (78.8-88.5)<br>(Moderna) 28+ d: ages 16-24= 78% (-56.3-96.9)<br>(Moderna) 28+ d: ages 25-34= 100% (N/R)<br>(Moderna) 28+ d: ages 35-44= 88.6% (54.5-97.2)<br>(Moderna) 28+ d: ages 45-54= 96.9% (73.8-99.6)<br>(Moderna) 28+ d: ages 55-64= 86.4% (67.3-94.4)<br>(Moderna) 28+ d: ages 65-74= 91.8% (81.7-96.3)<br>(Moderna) 28+ d: ages 75-84= 85.8% (77.1-91.2)<br>(Moderna) 28+ d: ages 85+= 82.9% (74.8-88.4)<br>(AstraZeneca) 7+ d: ages 16-24= 68.5% (29.9-85.9)<br>(AstraZeneca) 7+ d: ages 25-34= 77.2% (62.8-86.1)<br>(AstraZeneca) 7+ d: ages 35-44= 68.6% (60.8-74.9)<br>(AstraZeneca) 7+ d: ages 45-54= 73.5% (70.3-76.5)<br>(AstraZeneca) 7+ d: ages 55-64= 68.3% (64.1-72)<br>(AstraZeneca) 7+ d: ages 65-74= 72.2% (33.2-88.5)<br>(AstraZeneca) 7+ d: ages 75-84= 64.8% (-9.2-88.7)<br>(AstraZeneca) 7+ d: ages 85+= 38.7% (0-91.4)<br>(AstraZeneca) 14+ d: ages 16-24= 78.1% (32-92.9)<br>(AstraZeneca) 14+ d: ages 25-34= 82.4% (66.1-90.8)<br>(AstraZeneca) 14+ d: ages 35-44= 75.2% (66.7-81.5)<br>(AstraZeneca) 14+ d: ages 45-54= 75.9% (72.2-79)<br>(AstraZeneca) 14+ d: ages 55-64= 69.8% (65-73.9)<br>(AstraZeneca) 14+ d: ages 65-74= 52.2% (0-80.2)<br>(AstraZeneca) 14+ d: ages 75-84= 40.7% (0-80.9)<br>(AstraZeneca) 14+ d: ages 85+= 100% (N/R)<br>(AstraZeneca) 28+ d: ages 16-24= 65.8% (-36.7-91.5)<br>(AstraZeneca) 28+ d: ages 25-34= 87% (59.7-95.8)<br>(AstraZeneca) 28+ d: ages 35-44= 77% (64.2-85.2)<br>(AstraZeneca) 28+ d: ages 45-54= 77.9% (72.7-82.1)<br>(AstraZeneca) 28+ d: ages 55-64= 64.9% (57.3-71.1)

				(AstraZeneca) 28+ d: ages 65-74= 10.7% (-257.5-77.7)<br>(AstraZeneca) 28+ d: ages 75-84= 100% (N/R)<br>(AstraZeneca) 28+ d: ages 85+= 100% (N/R)
		Death		(Pfizer) 7+ d: ages 16-24= 100% (N/R)<br>(Pfizer) 7+ d: ages 25-34= 100% (N/R)<br>(Pfizer) 7+ d: ages 35-44= 100% (N/R)<br>(Pfizer) 7+ d: ages 45-54= 84.2% (66.8-92.5)<br>(Pfizer) 7+ d: ages 55-64= 92.7% (86.5-96.1)<br>(Pfizer) 7+ d: ages 65-74= 94.3% (91.6-96.1)<br>(Pfizer) 7+ d: ages 75-84= 91.3% (89.6-92.8)<br>(Pfizer) 7+ d: ages 85+= 87.1% (84.5-89.3)<br>(Pfizer) 14+ d: ages 16-24= 100% (N/R)<br>(Pfizer) 14+ d: ages 25-34= 100% (N/R)<br>(Pfizer) 14+ d: ages 35-44= 100% (N/R)<br>(Pfizer) 14+ d: ages 45-54= 87.7% (70.2-94.9)<br>(Pfizer) 14+ d: ages 55-64= 93.4% (86.8-96.7)<br>(Pfizer) 14+ d: ages 65-74= 93.8% (90.6-95.9)<br>(Pfizer) 14+ d: ages 75-84= 90.8% (88.8-92.4)<br>(Pfizer) 14+ d: ages 85+= 87% (84.2-89.4)<br>(Pfizer) 28+ d: ages 16-24= 100% (N/R)<br>(Pfizer) 28+ d: ages 25-34= 100% (N/R)<br>(Pfizer) 28+ d: ages 35-44= 100% (N/R)<br>(Pfizer) 28+ d: ages 45-54= 88.2% (68.5-95.6)<br>(Pfizer) 28+ d: ages 55-64= 94.8% (87.4-97.8)<br>(Pfizer) 28+ d: ages 65-74= 94.7% (90.9-96.9)<br>(Pfizer) 28+ d: ages 75-84= 91.7% (89.3-93.5)<br>(Pfizer) 28+ d: ages 85+= 88% (84.6-90.7)<br>(Moderna) 7+ d: ages 16-24= 100% (N/R)<br>(Moderna) 7+ d: ages 25-34= 100% (N/R)

				(Moderna) 7+ d: ages 35-44= 100% (N/R)<br>(Moderna) 7+ d: ages 45-54= 100% (N/R)<br>(Moderna) 7+ d: ages 55-64= 80.3% (20.9-95.1)<br>(Moderna) 7+ d: ages 65-74= 91.1% (78.7-96.3)<br>(Moderna) 7+ d: ages 75-84= 97% (92-98.9)<br>(Moderna) 7+ d: ages 85+= 92.5% (87-95.6)<br>(Moderna) 14+ d: ages 16-24= 100% (N/R)<br>(Moderna) 14+ d: ages 25-34= 100% (N/R)<br>(Moderna) 14+ d: ages 35-44= 100% (N/R)<br>(Moderna) 14+ d: ages 45-54= 100% (N/R)<br>(Moderna) 14+ d: ages 55-64= 72.4% (-10.8-93.1)<br>(Moderna) 14+ d: ages 65-74= 92.9% (78-97.7)<br>(Moderna) 14+ d: ages 75-84= 96.2% (90-98.6)<br>(Moderna) 14+ d: ages 85+= 93.3% (87.4-96.4)<br>(Moderna) 28+ d: ages 16-24= 100% (N/R)<br>(Moderna) 28+ d: ages 25-34= 100% (N/R)<br>(Moderna) 28+ d: ages 35-44= 100% (N/R)<br>(Moderna) 28+ d: ages 45-54= 100% (N/R)<br>(Moderna) 28+ d: ages 55-64= 70.5% (-110-95.9)<br>(Moderna) 28+ d: ages 65-74= 90.8% (63.1-97.7)<br>(Moderna) 28+ d: ages 75-84= 93.3% (82.2-97.5)<br>(Moderna) 28+ d: ages 85+= 94.8% (87.5-97.9)<br>(AstraZeneca) 7+ d: ages 16-24= 100% (N/R)<br>(AstraZeneca) 7+ d: ages 25-34= 100% (N/R)<br>(AstraZeneca) 7+ d: ages 35-44= 100% (N/R)<br>(AstraZeneca) 7+ d: ages 45-54= 74.3% (38-89.3)<br>(AstraZeneca) 7+ d: ages 55-64= 90.8% (77.8-96.2)<br>(AstraZeneca) 7+ d: ages 65-74= 100% (N/R)<br>(AstraZeneca) 7+ d: ages 75-84= 100% (N/R)<br>(AstraZeneca) 7+ d: ages 85+= 38.3% (-340-91.4)<br>(AstraZeneca) 14+ d: ages 16-24= 100% (N/R)

				(AstraZeneca) 14+ d: ages 25-34= 100% (N/R)<br>(AstraZeneca) 14+ d: ages 35-44= 100% (N/R)<br>(AstraZeneca) 14+ d: ages 45-54= 74.1% (30.6-90.3)<br>(AstraZeneca) 14+ d: ages 55-64= 88.7% (72.9-95.3)<br>(AstraZeneca) 14+ d: ages 65-74= 100% (N/R)<br>(AstraZeneca) 14+ d: ages 75-84= 100% (N/R)<br>(AstraZeneca) 14+ d: ages 85+= -2.6% (-631.8-85.6)<br>(AstraZeneca) 28+ d: ages 16-24= 100% (N/R)<br>(AstraZeneca) 28+ d: ages 25-34= 100% (N/R)<br>(AstraZeneca) 28+ d: ages 35-44= 100% (N/R)<br>(AstraZeneca) 28+ d: ages 45-54= 55.2% (-20.5-83.3)<br>(AstraZeneca) 28+ d: ages 55-64= 81.6% (55.6-92.4)<br>(AstraZeneca) 28+ d: ages 65-74= 100% (N/R)<br>(AstraZeneca) 28+ d: ages 75-84= 100% (N/R)<br>(AstraZeneca) 28+ d: ages 85+= 100% (N/R)
Y. Young-Xu (2021)	Pfizer and Moderna	PCR test positive infection<br><br><br><br>Symptomatic infection	14+ d: ages 18-64= 64% (50-74)<br>14+ d: ages 65+= 58% (52-64)<br>14+ d: ages 18-79= 58% (52-64)<br>14+ d: ages 80+= 57% (44-67)<br><br>14+ d: ages 18-64= 59% (37-74)<br>14+ d: ages 65+= 47% (37-56)<br>14+ d: ages 18-79= 50% (40-58)<br>14+ d: ages 80+= 50% (30-64)	14+ d: ages 18-64= 89% (79-95)<br>14+ d: ages 65+= 93% (90-95)<br>14+ d: ages 18-79= 62% (56-67)<br>14+ d: ages 80+= 63% (52-71)<br><br>14+ d: ages 18-64= 88% (63-96)<br>14+ d: ages 65+= 90% (84-94)<br>14+ d: ages 18-79= 90% (84-93)<br>14+ d: ages 80+= 91% (77-96)

Supplementary Table 4. Vaccine Efficacy reported within studies overtime (* indicates the time at which the VE decreased based on 95% CI)

Study (Publication year)	Vaccine	Outcome(s) Measured	Adjusted Vaccine Efficacy (95% CI)	
			After dose 1	After dose 2
N. Andrews[A] (2022)	Pfizer and Moderna, AstraZeneca	Symptomatic infection	Not reported	Pfizer) 2-4 wks: B.1.1.529= 65.5% (63.9-67)<br>(Pfizer) 5-9 wks: B.1.1.529=48.7 (47.1-50.2)*<br>(Pfizer) 10-14 wks: B.1.1.529= 30.1% (28.7-31.5)<br>(Pfizer) 15-19 wks: B.1.1.529= 15.4% (14.2-16.6)<br>(Pfizer) 20-24 wks: B.1.1.529= 11.5% (10.1-12.9)<br>(Pfizer) 25+ wks: B.1.1.529= 8.8% (7-10.5)<br>(Moderna) 2-4 wks: B.1.1.529= 75.1% (70.8-78.7)<br>(Moderna) 5-9 wks: B.1.1.529=52.8% (48.2-57.1)*<br>(Moderna) 10-14 wk: B.1.1.529= 35.6% (32.7-38.4)<br>(Moderna) 15-19 wk: B.1.1.529= 25.3% (23.2-27.4)<br>(Moderna) 20-24 wk: B.1.1.529= 15% (11.6-18.2)<br>(Moderna) 25+ wks: B.1.1.529= 14.9% (3.9-24.7)<br>(Pfizer) 2-4 wks: B.1.617.2= 90.9% (89.6-92)<br>(Pfizer) 5-9 wks: B.1.617.2=85.5% (84.5-86.5)*<br>(Pfizer) 10-14 wks: B.1.617.2= 78.7% (78-79.4)<br>(Pfizer) 15-19 wks: B.1.617.2= 74.4% (73.8-74.9)

				(Pfizer) 20-24 wks: B.1.617.2= 67.4% (66.5-68.2)<br>(Pfizer) 25+ wks: B.1.617.2= 62.7% (61.6-63.7)<br>(Moderna) 2-4 wks: B.1.617.2= 94.5% (90.5-96.9)<br>(Moderna) 5-9 wks: B.1.617.2=91.8% (89.6-93.6)<br>(Moderna) 10-14 wk: B.1.617.2= 84.1% (82.7-85.3)*<br>(Moderna) 15-19 wk: B.1.617.2= 82.8% (81.8-83.7)<br>(Moderna) 20-24 wk: B.1.617.2= 76.2% (74.7-77.7)<br>(Moderna) 25+ wks: B.1.617.2= 80.4% (67.3-88.2)<br>(AstraZeneca) 2-4 wks: B.1.1.529= 48.9% (39.2-57.1)<br>(AstraZeneca) 5-9 wks: B.1.1.529= 33.7% (25-41.5)<br>(AstraZeneca) 10-14 wk: B.1.1.529= 28.6% (20.9-35.6)*<br>(AstraZeneca) 15-19 wk: B.1.1.529= 17.8% (13.4-21.9)<br>(AstraZeneca) 20-24 wk: B.1.1.529= 4% (1.9-6.1)<br>(AstraZeneca) 25+ wks: B.1.1.529= -2.7% (-4.2--1.2)<br>(AstraZeneca) 2-4 wks: B.1.617.2= 82.8% (74.5-88.4)<br>(AstraZeneca) 5-9 wks: B.1.617.2= 76.5% (70.3-81.5)

				(AstraZeneca) 10-14 wks: B.1.617.2= 69.2% (64.7-73.1)*<br>(AstraZeneca) 15-19 wks: B.1.617.2= 53.6% (51.6-55.5)<br>(AstraZeneca) 20-24 wks: B.1.617.2= 47.4% (46.2-48.5)<br>(AstraZeneca) 25+ wks: B.1.617.2= 43.5% (42.4-44.5)
N. Andrews[B] (2022)	Pfizer and Moderna	Symptomatic infection<br><br><br><br><br><br><br><br><br><br><br><br><br><br><br><br><br><br><br><br><br>Hospitalization	Not reported	(Pfizer) 1 wk: B.1.617.2 = 92.3% (92-92.6)<br>(Pfizer) 2-9 wk: B.1.617.2= 89.7% (89.5-89.8)<br>(Pfizer) 10-14 wk: B.1.617.2= 80.7% (80.3-81)*<br>(Pfizer) 15-19 wk: B.1.617.2= 72.8% (72.4-73.2)<br>(Pfizer) 20+ wk: B.1.617.2= 66.3% (65.7-66.9)<br>(Pfizer) 1 wk: B.1.1.7= 90.2% (86.9-92.7)<br>(Pfizer) 2-9 wk: B.1.1.7= 94.9% (93.6-95.9)<br>(Pfizer) 10+ wk: B.1.1.7= 94.8% (88.4-97.7)*<br>(Moderna) 1 wk: B.1.617.2= 95.6% (95-96.2)<br>(Moderna) 2-9 wk: B.1.617.2= 93.8% (93.4-94.1)<br>(Moderna) 10-14 wk: B.1.617.2= 85.6% (83.8-87.2)*<br>(AstraZeneca) 1 wk: B.1.617.2= 64.8% (63.8-65.8)<br>(AstraZeneca) 2-9 wk: B.1.617.2= 67.6% (67.3-67.9)<br>(AstraZeneca) 10-14 wk: B.1.617.2= 57.9% (57.5-58.4)*<br>(AstraZeneca) 15-19 wk: B.1.617.2= 50.5% (49.8-51.1)<br>(AstraZeneca) 20+ wk: B.1.617.2= 44.3% (43.2-45.4)

		Death		(AstraZeneca) 1 wk: B.1.1.7= 71.8% (66.2-76.5)<br>(AstraZeneca) 2-9 wk: B.1.1.7= 82.4% (79.6-84.7)<br>(AstraZeneca) 10+ wk: B.1.1.7= 76.2% (49.8-88.7)<br><br>(Pfizer) 1 wk: B.1.617.2= 99.4% (97.7-99.9)<br>(Pfizer) 2-9 wk: B.1.617.2= 98.7% (98.3-99)<br>(Pfizer) 10-14 wk: B.1.617.2= 96.8% (96.3-97.3)*<br>(Pfizer) 15-19 wk: B.1.617.2= 94.9% (94.1-95.5)<br>(Pfizer) 20+ wk: B.1.617.2= 91.7% (90.2-93)<br>(AstraZeneca) 1 wk: B.1.617.2= 94% (91.3-95.8)<br>(AstraZeneca) 2-9 wk: B.1.617.2= 95.2% (94.7-95.7)<br>(AstraZeneca) 10-14 wk: B.1.617.2= 92.1% (91.3-92.7)*<br>(AstraZeneca) 15-19 wk: B.1.617.2= 87.4% (86.1-88.6)<br>(AstraZeneca) 20+ wk: B.1.617.2= 80% (76.8-82.7)<br><br>(Pfizer) 2-9 wk: B.1.617.2= 98.5% (96.5-99.3)<br>(Pfizer) 10-14 wk: B.1.617.2= 96% (94.2-97.2)<br>(Pfizer) 15-19 wk: B.1.617.2= 94.5% (92.5-96)<br>(Pfizer) 20+ wk: B.1.617.2= 91.9% (88.5-94.3)*<br>(AstraZeneca) 2-9 wk: B.1.617.2= 95% (93.1-96.4)<br>(AstraZeneca) 10-14 wk: B.1.617.2= 93.7% (91.8-95.2)

				(AstraZeneca) 15-19 wk: B.1.617.2= 90.1% (86.9-92.6)*<br>(AstraZeneca) 20+ wk: B.1.617.2= 84.8% (76.2-90.3)
S. Bedston (2022)	Pfizer	PCR test positive infection	Not reported	2 wk: 67% (N/R)<br>2-5 wk: 86% (N/R)<br>6-13 wk: 77% (N/R)<br>14-25 wk: 53% (N/R)<br>26 wk: 45% (N/R)
F.P. Bianchi[B] (2021)	Pfizer	PCR test positive infection<br><br><br><br><br>Symptomatic infection	Not reported	14-41 d: 94.8% (87-97.8)<br>42-69 d: 83% (65-92)<br>69+ d: 81% (42-94)<br><br>14-41 d: 97.2% (90.3-99.2)<br>42-69 d: 85% (63-94.2)<br>69+ d: 88% (42-97.6)
K.J. Bruxvoort (2021)	Moderna	PCR test positive infection	Not reported	14-60 d: B.1.617.2= 94.1% (90.5-96.3)<br>61-90 d: B.1.617.2= 88.7% (85-91.5)<br>91-120 d: B.1.617.2= 85.9% (81.1-89.5)*<br>121-150 d: B.1.617.2= 77% (69.1-82.9)<br>151-180 d: B.1.617.2= 80% (70.2-86.6)<br>14-60 d: non-B.1.617.2= 98.6% (97.3-99.3)<br>61-90 d: non-B.1.617.2= 96.9% (93.9-98.5)<br>91-120 d: non-B.1.617.2= 91.4% (83.9-95.4)*<br>121-150 d: non-B.1.617.2= 88.7% (73.2-95.2)<br>14-60 d: unidentified= 83.6% (79.5-86.9)<br>61-90 d: unidentified= 82.2% (77-86.2)<br>91-120 d: unidentified= 77.7% (70.7-83)<br>121-150 d: unidentified= 66.4% (53.6-75.6)*<br>151-180 d: unidentified= 68.5% (51.3-79.6)

T. Cerqueira-Silva (2022)	Pfizer, AstraZeneca	Symptomatic infection<br><br><br><br><br>Hospitalization, Death	Not reported	(Pfizer) 14-90 d: 64.2% (54.2-72)<br>(Pfizer) 90+ d: 100% (N/R)<br>(AstraZeneca) 14-90 d: 55.5% (50.5-60.1)<br>(AstraZeneca) 90+ d: 56.8% (46.6-65.1)<br><br>(Pfizer) 14-90 d: 88.8% (50-97.5)<br>(Pfizer) 90+ d: 100% (N/R)<br>(AstraZeneca) 14-90 d: 86.6% (77.6-92)<br>(AstraZeneca) 90+ d: 95.1% (84.8-98.4)
H. Chung (2021)	Pfizer and Moderna	Symptomatic infection	14-20 d: 48% (41-54)<br>35-41 d: 71% (63-78)	
A. Florea (2022)	Moderna	PCR positive infection<br><br><br><br><br>Hospitalization	Not reported	0-2 mo: 88% (86.8-89.1)<br>2-4 mo: 84.5% (83.8-85.2)*<br>4-6 mo: 77% (75.7-78.2)<br>6-8 mo: 75.5% (70.4-79.7)<br><br>0-2 mo: 95.9% (93.5-97.4)<br>2-4 mo: 97.4% (96.6-98)<br>4-6 mo: 94.8% (93.6-95.7)<br>6-8 mo: 94.5% (90.9-96.7)
V. Hall[B] (2022)	Pfizer and AstraZeneca	PCR test positive infection	(Pfizer) 21-27 d: 59% (42-71)<br>(Pfizer) 28-41 d: 66% (52-76)<br>(Pfizer) 42-55 d: 70% (54-81)<br>(Pfizer) 56-280 d: 63% (46-75)<br><br>(AstraZeneca) 28-41 d: 85% (16-97)<br>(AstraZeneca) 42-55 d: 32% (-87-75)<br>(AstraZeneca) 56-249 d: 9% (-87-55)	(Pfizer) 14-73 d: 85% (72-92)<br>(Pfizer) 74-133 d: 66% (53-75)<br>(Pfizer) 134-193 d: 68% (54-77)<br>(Pfizer) 194-239 d: 51% (22-69)*<br><br>(AstraZeneca) 74-133 d: 50% (29-65)<br>(AstraZeneca) 134-220 d: 72% (39-87)
S.V. Katikireddi (2022)	AstraZeneca	Symptomatic infection	Not reported	2-3 wk: 67.9% (65.9-69.8)<br>4-5 wk: 67.3% (65.3-69.1)<br>6-7 wk: 63.8% (61.7-65.7)*

				8-9 wk: 63.3% (61.3-65.3)<br>10-11 wk: 59.3% (57.2-61.4)<br>12-13 wk: 55.3% (53-57.5)<br>14-15 wk: 52.9% (50.4-55.2)<br>16-17 wk: 48.7% (45.9-51.4)<br>18-19 wk: 44.6% (41.5-47.6)<br>20-21 wk: 39.1% (35.4-42.6)
		Hospitalization/ Death		2-3 wk: 83.7% (79.7-87)<br>4-5 wk: 86.6% (83.6-89)<br>6-7 wk: 86.8% (84.2-88.9)<br>8-9 wk: 79% (75.9-81.7)*<br>10-11 wk: 79.6% (76.8-82.1)<br>12-13 wk: 77.4% (74.6-80)<br>14-15 wk: 75.9% (72.9-78.6)<br>16-17 wk: 70.5% (67-73.7)<br>18-19 wk: 63.7% (59.6-67.4)<br>20-21 wk: 53.6% (48.4-58.3)
A.S Lauring (2022)	Pfizer and Moderna	Hospitalization	Not reported	14-150 d: B.1.617.2= 88% (86-90)<br>150+ d: B.1.617.2= 81% (78-84)
Lopez Bernal (2021)[B]	Pfizer	Symptomatic infection	(Pfizer) 21-55 d: B.1.1.7= 56.9% (48.6-63.8)<br>(Pfizer) 21-55 d: B.1.617.2= 32.7% (7-51.3)<br>(Pfizer) 56+ d: B.1.1.7= 41.3% (33.4-48.2)<br>(Pfizer) 56+ d: B.1.617.2= 36.7% (21.3-49.1)<br>(AstraZeneca) 21-55 d: B.1.1.7= 49.7% (45.9-53.2)<br>(AstraZeneca) 21-55 d: B.1.617.2= 34.6% (27.7-40.8)	

			(AstraZeneca) 56+ d: B.1.1.7= 45.9% (40.4-51)<br>(AstraZeneca) 56+ d: B.1.617.2= 25.5% (18-32.3)	
H. Maeda (2022)	Pfizer and Moderna	Symptomatic infection	Not reported	1-3 mo: ages 16-64= 91.8% (80.3-96.6)<br>4-6 mo: ages 16-64= 86.5% (56.9-95.7)
McKeigue (2022)	Pfizer Moderna and AstraZeneca	Severe infection<br><br><br>Hospitalization	Not reported	(Pfizer/Moderna) 20 wks: 93% (88-96)<br>(AstraZeneca) 20 wks: 69% (52-80)<br><br>(Pfizer/Moderna) 20 wks: 89% (86-91)<br>(AstraZeneca) 20 wks: 58% (50-64)
C. Menni (2021)	Pfizer	PCR positive infection	21-44 d: 69% (66-72)<br>45-59 d: 72% (63-79)	
P. Nordstrom (2022)	Pfizer and Moderna, AstraZeneca	PCR positive infection		(Pfizer) 15-30 d: 92% (92-93)<br>(Pfizer) 31-60 d: 89% (88-90)*<br>(Pfizer) 61-120 d: 84% (84-85)<br>(Pfizer) 121-180 d: 47% (39-55)<br>(Pfizer) 181-210 d: 29% (15-41)<br>(Pfizer) 210+ d: 23% (-2-41)<br>(Moderna) 15-30 d: 96% (94-97)<br>(Moderna) 31-60 d: 93% (90-94)<br>(Moderna) 61-120 d: 85% (82-88) *<br>(Moderna) 121-180 d: 71% (56-80)<br>(Moderna) 180+ d: 59% (18-79)<br>(AstraZeneca) 15-30 d: 68% (52-79)<br>(AstraZeneca) 31-60 d: 49% (28-64)<br>(AstraZeneca) 61-120 d: 41% (29-51)*<br>(AstraZeneca) 120+ d: -19% (-98-28)
C.R. Oliveira (2022)	Pfizer	PCR positive infection	Not reported	1-4 wks: 91% (33-99)<br>5-8 wks: 90% (67-97)<br>9-12 wks: 95% (79-99)

				13-17 wks: 83% (34-95)
E. Poukka (2022)	Pfizer,<br>Moderna,<br>AstraZeneca	PCR test positive infection<br><br><br><br><br><br><br><br><br><br><br><br><br>Hospitalization	(Pfizer/Moderna) 21-41 d: 48% (39-55)<br>(Pfizer/Moderna) 42+ d: 40% (34-46)<br>(Pfizer/Moderna) 21-41 d: non-B.1.617.2= 40% (25-52)<br>(Pfizer/Moderna) 42+ d: non-B.1.617.2= 38% (23-50)<br>(Pfizer/Moderna) 21-41 d: B.1.617.2= 56% (46-64)<br>(Pfizer/Moderna) 42+ d: B.1.617.2= 45% (37-51)<br>(Pfizer) 21-41 d: 47% (38-55)<br>(Pfizer) 42+ d: 40% (33-46)<br>(Moderna) 21-41 d: 72% (47-85)<br>(Moderna) 42+ d: 61% (45-72)<br>(AstraZeneca) 21-41 d: 7% (-24-30)<br>(AstraZeneca) 42+ d: 22% (-3-42)<br>(AstraZeneca) 21-41 d: non-B.1.617.2= 6% (-26-29)<br>(AstraZeneca) 42+ d: non-B.1.617.2= 15% (-15-37)<br>(AstraZeneca) 21-41 d: B.1.617.2= 100% (N/R)<br>(AstraZeneca) 42+ d: B.1.617.2= 49% (-16-77)<br><br><br><br>(Pfizer/Moderna) 21-41 d: 95% (67-99)<br>(Pfizer/Moderna) 42+ d: 83% (70-91)	(Pfizer/Moderna) 14-90 d: 82% (79-85)<br>(Pfizer/Moderna) 91-180 d: 62% (55-68)*<br>(Pfizer/Moderna) 181+ d: 53% (43-62)<br>(Pfizer/Moderna) 14-90 d: non-B.1.617.2= 77% (71-82)<br>(Pfizer/Moderna) 91-180 d: non-B.1.617.2= 55% (34-69)*<br>(Pfizer/Moderna) 91-180 d: B.1.617.2= 65% (58-71)<br>(Pfizer/Moderna) 14-90 d: B.1.617.2= 85% (81-88)<br>(Pfizer/Moderna) 181+ d: B.1.617.2= 56% (46-56)*<br>(Pfizer) 14-90 d: 83% (80-85)<br>(Pfizer) 91-180 d: 63% (56-69)*<br>(Pfizer) 181+ d: 55% (45-64)<br>(Moderna) 14-90 d: 84% (68-92)<br>(Moderna) 91-180 d: 69% (-124-96)<br>(AstraZeneca) 14-90 d: 89% (73-95)<br>(AstraZeneca) 91-180 d: 63% (-166-95)<br>(AstraZeneca) 14-90 d: non-B.1.617.2= 100% (N/R)<br>(AstraZeneca) 91-180 d: non-B.1.617.2= 100% (N/R)<br>(AstraZeneca) 14-90 d: B.1.617.2= 88% (71-95)<br>(AstraZeneca) 91-180 d: B.1.617.2= 62% (-177-95)<br><br>(Pfizer/Moderna) 14-90 d: 99% (97-100)<br>(Pfizer/Moderna) 91-180 d: 98% (91-99)

			(Pfizer/Moderna) 21-41 d: non-B.1.617.2= 89% (15-98)<br>(Pfizer/Moderna) 42+ d: non-B.1.617.2= 90% (27-99)<br>(Pfizer/Moderna) 21-41 d: B.1.617.2= 100% (N/R)<br>(Pfizer/Moderna) 42+ d: B.1.617.2= 83% (68-91)<br>(Pfizer) 21-41 d: 95% (63-99)<br>(Pfizer) 42+ d: 82% (68-90)<br>(Moderna) 21-41 d: 100% (N/R)<br>(Moderna) 42+ d: 89% (22-98)<br>(AstraZeneca) 21-41 d: -17% (-156-47)<br>(AstraZeneca) 42+ d: 88% (10-98)<br>(AstraZeneca) 21-41 d: non-B.1.617.2= -13% (-149-49)<br>(AstraZeneca) 42+ d: non-B.1.617.2= 100% (N/R)<br>(AstraZeneca) 21-41 d: B.1.617.2= 100% (N/R)<br>(AstraZeneca) 42+ d: B.1.617.2= 42% (-330-92)	(Pfizer/Moderna) 181+ d: 98% (89-100)<br>(Pfizer/Moderna) 14-90 d: non-B.1.617.2= 95% (64-99)<br>(Pfizer/Moderna) 91-180 d: non-B.1.617.2= 100% (N/R)<br>(Pfizer/Moderna) 14-90 d: B.1.617.2= 100% (97-100)<br>(Pfizer/Moderna) 91-180 d: B.1.617.2= 98% (90-99)<br>(Pfizer/Moderna) 181+ d: B.1.617.2= 98% (88-100)<br>(Pfizer) 14-90 d: 99% (97-100)<br>(Pfizer) 91-180 d: 98% (91-99)<br>(Pfizer) 181+ d: 98% (89-100)<br>(Moderna) 14-90 d: 100% (N/R)<br>(Moderna) 91-180 d: 100% (N/R)<br>(Moderna) 181+ d: 100% (N/R)<br>(AstraZeneca) 14-90 d: 100% (N/R)<br>(AstraZeneca) 91-180 d: 88% (48-97)<br>(AstraZeneca) 181+ d: 100% (N/R)<br>(AstraZeneca) 14-90 d: non-B.1.617.2= 100% (N/R)<br>(AstraZeneca) 91-180 d: B.1.617.2= 81% (9-96)<br>(AstraZeneca) 14-90 d: B.1.617.2= 100% (N/R)<br>(AstraZeneca) 181+ d: B.1.617.2= 100% (N/R)
K.B. Pouwels (2021) UK	Pfizer and AstraZeneca	PCR test positive infection (18-64 years old, B.1.617.2)	Not reported	Pfizer) 14 d: 85% (79-90)<br>(Pfizer) 30 d: 83% (78-88)<br>(Pfizer) 60 d: 80% (76-83)<br>(Pfizer) 90 d: 75% (70-80)<br>(AstraZeneca) 14 d: 68% (61-73)<br>(AstraZeneca) 30 d: 66% (61-71)

		Symptomatic infection		(AstraZeneca) 60 d: 64% (58-69)<br>(AstraZeneca) 90 d: 61% (53-68)<br><br>Pfizer) 14 d: 93% (89-96)<br>(Pfizer) 30 d: 92% (87-95)<br>(Pfizer) 60 d: 86% (82-90)<br>(Pfizer) 90 d: 78% (72-82)*<br>(AstraZeneca) 14 d: 72% (64-78)<br>(AstraZeneca) 30 d: 70% (64-76)<br>(AstraZeneca) 60 d: 67% (60-72)<br>(AstraZeneca) 90 d: 63% (53-71)
A.M. Price (2022)	Pfizer	Hospitalization	Not reported	2-22 wks: B.1.617.2= 93% (89-95)<br>23-44 wks: B.1.617.2= 92% (80-97)<br>2-22 wks: B.1.1.529= 43% (-1-68)*<br>23-44 wks: B.1.1.529= 38% (-3-62)
D.M. Skowronski (2021)	Pfizer and Moderna	PCR test positive	14-20 d: 43% (30-53)<br>21-27 d: 57% (47-66)<br>28-34 d: 69% (59-77)<br>35-41 d: 75% (63-83)<br>42+ d: 63% (48-74)<br>14-20 d: B.1.1.7= 48% (30-61)<br>21-27 d: B.1.1.7= 55% (37-67)<br>28-34 d: B.1.1.7= 76% (62-85)<br>35-41 d: B.1.1.7= 81% (64-90)<br>42+ d: B.1.1.7= 67% (44-81)<br>14-20 d: P.1= 24% (-7-46)<br>21-27 d: P.1= 52% (26-69)<br>28-34 d: P.1= 66% (42-80)<br>35-41 d: P.1= 68% (35-85)<br>42+ d: P.1= 53% (12-75)<br>(Pfizer) 14-20 d: 42% (28-52)	Not reported

			(Pfizer) 21-27 d: 54% (41-63)<br>(Pfizer) 28-34 d: 69% (58-77)<br>(Pfizer) 35-41 d: 75% (61-84)<br>(Pfizer) 42+ d: 66% (51-77)<br>(Moderna) 14-20 d: 49% (19-68)<br>(Moderna) 21-27 d: 78% (55-89)<br>(Moderna) 28-34 d: 68% (34-85)<br>(Moderna) 35-41 d: 75% (39-90)<br>(Moderna) 42+ d: 42% (-28-74)	
J.L. Suah[B] (2022)	Pfizer	PCR positive infection<br><br><br>ICU Admission<br><br><br>Death	Not reported	14 d-2 mo: 90.8% (89.4-92.1)<br>3-5 mo: 79.3% (76.1-82.1)*<br><br>14 d-2 mo: 86% (82.8-88.6)<br>3-5 mo: 77.5% (71.7-82.1)*<br><br>14 d-2 mo: 91.5% (89.8-92.9)<br>3-5 mo: 91.2% (88.6-93.1)
S.Y. Tartof [A] (2021)	Pfizer	PCR test positive infection	Not reported	7-36 d: 88% (86-89)<br>37-66 d: 84% (83-86)<br>67-96 d: 78% (76-79)*<br>97-126 d: 68% (65-70)<br>127-156 d: 61% (58-64)<br>157+ d: 47% (43-51)<br>7-36 d: B.1.617.2= 93% (85-97)<br>37-66 d: B.1.617.2= 88% (81-92)<br>67-96 d: B.1.617.2= 78% (70-83)*<br>97-126 d: B.1.617.2= 60% (48-69)<br>127+ d: B.1.617.2= 53% (39-65)<br>7-36 d: non-B.1.617.2= 97% (95-99)<br>37-66 d: non-B.1.617.2= 91% (87-94)*<br>67-96 d: non-B.1.617.2= 88% (81-92)

				97-126 d: non-B.1.617.2= 80% (69-87)<br>127+ d: non-B.1.617.2= 67% (45-80)<br>7-36 d: failed sequence= 83% (77-87)<br>37-66 d: failed sequence= 72% (65-77)<br>67-96 d: failed sequence= 72% (64-78)<br>97-126 d: failed sequence= 64% (54-73)*<br>127+ d: failed sequence= 47% (30-59)
		Hospitalization		7-36 d: 87% (82-91)<br>37-66 d: 88% (83-92)<br>67-96 d: 92% (89-95)<br>97-126 d: 93% (89-95)<br>127-156 d: 91% (87-93)<br>157+ d: 88% (82-92)
S.Y Tartof [B] (2022)	Pfizer	Hospitalization	Not reported	7 d-3 mo: B.1.617.2= 78% (55-89)<br>7 d-3 mo: B.1.1.529= 68% (48-80)<br>3-6 mo: B.1.617.2= 87% (76-93)<br>3-6 mo: B.1.1.529= 68% (53-78)<br>6-9 mo: B.1.617.2= 72% (60-81)<br>6-9 mo: B.1.1.529= 72% (63-79)<br>9+ mo: B.1.617.2= 73% (58-83)<br>9+ mo: B.1.1.529= 41% (21-55)
		Emergency department admission		7 d-3 mo: B.1.617.2= 78% (69-85)<br>7 d-3 mo: B.1.1.529= 64% (51-73)<br>3-6 mo: B.1.617.2= 59% (48-68)*<br>3-6 mo: B.1.1.529= 47% (34-57)<br>6-9 mo: B.1.617.2= 59% (51-66)<br>6-9 mo: B.1.1.529= 51% (43-59)<br>9+ mo: B.1.617.2= 57% (45-66)<br>9+ mo: B.1.1.529= 31% (16-43)

S.J. Thomas (2021)	Pfizer	PCR test positive infection	Not reported	7d- 2mo: 96.2% (93.3-98.1)<br>2mo-4mo: 90.1% (86.6-92.9)*<br>4mo-6mo: 83.7% (74.7-89.9)
H.F. Tseng (2022)	Pfizer	PCR test positive infection	Not reported	14-90 d: B.1.617.2= 80.2% (68.2-87.7)<br>91-180 d: B.1.617.2= 68.9% (60.1-75.8)<br>181-270 d: B.1.617.2= 63.7% (59.8-67.2)*<br>270+ d: B.1.617.2= 61.3% (55-66.7)<br>14-90 d: B.1.1.529= 44% (35.1-51.6)<br>91-180 d: B.1.1.529= 23.5% (16.4-30)<br>181-270 d: B.1.1.529= 13.8% (10.2-17.3)*<br>270+ d: B.1.1.529= 5.9% (0.4-11)
E. Vasileiou, (2021)	Pfizer, AstraZeneca	Hospitalization	(Pfizer) 14-20 d: 69% (62-75)<br>(Pfizer) 21-27 d:78% (71-83)<br>(Pfizer) 28-34 d: 91% (85-94)<br>(Pfizer) 35-41 d:78% (69-85)<br>(Pfizer) 42+ d: 77% (68-83)<br>(AstraZeneca) 14-20 d: 73% (66-79)<br>(AstraZeneca) 21-27 d:81% (72-87)<br>(AstraZeneca) 28-34 d: 88% (75-94)<br>(AstraZeneca) 35-41 d: 97% (63-100)	Not reported
B.J. Wright (2022)	Pfizer and Moderna	Hospitalization	Not reported	(Moderna) 50-100 d: 97.3% (96-98.2)<br>(Moderna) 200-250 d: 87.6% (84.5-90.1)*<br>(Pfizer) 50-100 d: 94.9% (93.2-96.2)<br>(Pfizer) 200-250 d: 74.1% (69.6-77.9)*
